# The Influence of 5′*R* and 5′*S* cdA and cdG on the Activity of BsmAI and SspI Restriction Enzymes

**DOI:** 10.3390/molecules26123750

**Published:** 2021-06-19

**Authors:** Michał Szewczuk, Karolina Boguszewska, Julia Kaźmierczak-Barańska, Bolesław T. Karwowski

**Affiliations:** DNA Damage Laboratory of Food Science Department, Faculty of Pharmacy, Medical University of Lodz, ul. Muszynskiego 1, 90-151 Lodz, Poland; michal.szewczuk@umed.lodz.pl (M.S.); karolina.boguszewska@umed.lodz.pl (K.B.); julia.kazmierczak-baranska@umed.lodz.pl (J.K.-B.)

**Keywords:** 5′,8-cyclo-2′-deoxyadenosine (cdA), 5′,8-cyclo-2′-deoxyguanosine (cdG), tandem lesions, clustered lesions, base excision repair (BER), BsmAI, SspI, restriction endonucleases

## Abstract

Restriction endonucleases (REs) are intra-bacterial scissors that are considered tools in the fight against foreign genetic material. SspI and BsmAI, examined in this study, cleave dsDNA at their site of recognition or within a short distance of it. Both enzymes are representatives of type II REs, which have played an extremely important role in research on the genetics of organisms and molecular biology. Therefore, the study of agents affecting their activity has become highly important. Ionizing radiation may damage basic cellular mechanisms by inducing lesions in the genome, with 5′,8-cyclo-2′-deoxypurines (cdPus) as a model example. Since cdPus may become components of clustered DNA lesions (CDLs), which are unfavorable for DNA repair pathways, their impact on other cellular mechanisms is worthy of attention. This study investigated the influence of cdPus on the elements of the bacterial restriction–modification system. In this study, it was shown that cdPus present in DNA affect the activity of REs. SspI was blocked by any cdPu lesion present at the enzyme’s recognition site. When lesions were placed near the recognition sequence, the SspI was inhibited up to 46%. Moreover, (5′*S*)-5′,8-cyclo-2′-deoxyadenosine (ScdA) present in the oligonucleotide sequence lowered BsmAI activity more than (5′*R*)-5′,8-cyclo-2′-deoxyadenosine (RcdA). Interestingly, in the case of 5′,8-cyclo-2′-deoxyguanosine (cdG), both 5′*S* and 5′*R* diastereomers inhibited BsmAI activity (up to 55% more than cdA). The inhibition was weaker when cdG was present at the recognition site rather than the cleavage site.

## 1. Introduction

Cellular survival and development depend on the stability of genetic information and the cell’s ability to duplicate it. DNA is affected by numerous factors, which may induce up to a million DNA lesions per day, per cell [1,2]. Prokaryotic and eukaryotic organisms have DNA repair systems that continuously correct errors and/or lesions in the genome. Some lesions, e.g., 5′,8-cyclo-2′-deoxypurines (cdPus), are challenging to detect and/or remove [3,4]. They may generate mutations and subsequently lead to pathologies, e.g., carcinogenesis [5,6]. This type of DNA damage has received growing interest, as it is present in various organisms [7]. Numerous studies have assigned the level of cdPus in human genetic material as follows: 0.01 (RcdA), 1 (ScdA), 2 (RcdG), and 10 (ScdG) lesions per 10^6^ nucleosides; for details, please see reference [5].

CdPus are formed as a result of •OH action on the H5′-atom of the sugar moiety of 2′-deoxyadenosine or 2′-deoxyguanosine [8,9]. 5′*R* and 5′*S* diastereomers of cdPus may change the spatial structure of the DNA helix, especially 5′ to the lesion [8]. CdPus may be a part of clustered DNA lesions (CDLs) that perturb DNA helix twist and base-pair stacking [9,10]. CDLs are defined as the occurrence of at least 2 lesions per 1–2 DNA helix turns. Furthermore, cdPus gained attention due to their potential medical usefulness in cancer diagnosis and evaluation of treatment effectiveness [11,12]. When unrepaired, they can inhibit or block gene expression, replication, and the activity of proteins involved in DNA repair and trigger subsequent mutagenesis [7,9,13,14].

Restriction endonucleases (REs) require specific nucleotide sequences to recognize, bind, and cleave DNA [15,16]. Based on their properties and specificity, different REs may cleave one or both DNA strands within or next to the recognition site and create sticky or blunt ends [17,18]. Type II REs, which require highly specific sites for DNA cleavage, are of particular importance in molecular biology and are widely used as a tool for DNA modification (e.g., cloning and purification of specific DNA fragments) [18,19]. What is more, the main biological significance of REs is that they are a part of the bacterial restriction-modification system, which protects the cell from foreign DNA (e.g., viral DNA) [17,20]. CdPus may potentially lower the activity of REs, thus impairing the ability of prokaryotes to defend against external genetic material [17,20]. CdPus may also lower the ability of eukaryotic cells to manage DNA lesions by inhibition of proteins responsible for genome repair [13,14,21].

**Figure 1:**
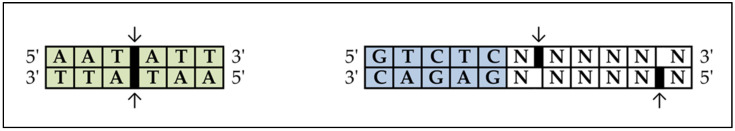
The sequences of recognition and cleavage sites of SspI and BsmAI.

SspI recognizes palindromic sequences of 5′-AATATT-3′ and cleaves both DNA strands between T3 and A4 of the sequence, generating blunt ends (Figure 1). It is widely used as a tool for molecular biology, e.g., differentiation of IBDV (infectious bursal disease virus) strains and animal species [22,23,24].

BsmAI, a type of IIS endonuclease, recognizes asymmetric DNA sequences and cleaves the strand downstream (within 1–2 helical turns). This distance coincides with the distance covered by CDLs, which may indicate a possible impact of CDLs on enzyme activity. BsmAI recognizes and cleaves both strands of the dsDNA N1/N5 downstream from the specific sequence of 5′-GTCTC-3′ using two separate catalytic sites within the protein structure [18]. After DNA incision, it generates sticky ends with the extension of four bases in the 5′-end direction [19,25,26]. BsmAI also exists as a recombinant dsDNA-nicking protein, which was engineered to cleave only one strand of DNA. This may indicate that with one catalytic site inactivated, the enzyme may still nick DNA using its second catalytic site [26].

REs can cut DNA molecules in regions with a well-defined sequence [15]. Owing to this ability, they are used to introduce isolated genes into vectors, which are most often bacterial plasmids. Vectors containing foreign DNA can be duplicated, which is why REs are widely used in genetic engineering and applied biotechnology [27,28,29]. Therefore, knowledge of the ways to increase or inhibit RE activity may be important for genetic engineering techniques. Damage occurs constantly in cells and may be a factor limiting the proper functioning of, for example, RE tools.

The present study examined the influence of the cdPus (5′*S*)-5′,8-cyclo-2′-deoxyadenosine (ScdA), (5′*R*)-5′,8-cyclo-2′-deoxyadenosine (RcdA), (5′*S*)-5′,8-cyclo-2′-deoxyguanosine (ScdG), and (5′*R*)-5′,8-cyclo-2′-deoxyguanosine (RcdG) on the activity of BsmAI and SspI. This study used dsDNA with cdPus in one or both DNA strands located within or next to the recognition sites of REs.

## 2. Materials and Methods

### 2.1. Substrate Oligonucleotide Synthesis and Purification

The oligonucleotides were synthesized and purified in the Bioorganic Chemistry Department, Polish Academy of Science, Lodz, Poland, using a Geneworld synthesizer (K & A Laborgeraete GbR, Schaafheim, Germany) and nucleotide phosphoramidites (ChemGenes Corporation, Wilmington, MA, USA). The phosphoramidite derivatives of (5′*R*)/(5′*S*) cdA and cdG were synthesized according to Romieu et al. [30]. The crude oligonucleotides were purified by HPLC using Varian analytics with UV detection at wavelengths of λ = 260 nm and a Phenomenex C-18 column (Synergi 4 µm Fusion-RP 80Å, 250 × 4.6 mm). The complete sequences of substrate ss oligonucleotides are available in the Supplementary Materials (Table S1).

### 2.2. Mass Spectroscopy of Oligonucleotides

Oligonucleotides were analyzed in the negative-ion mode on a Waters Synapt G2-Si HDMS quadrupole time-of-flight hybrid mass spectrometer (Waters, Manchester, UK). Samples were dissolved in 10 mM ammonium acetate with 50% acetonitrile to obtain a final concentration of 0.1 OD/mL. Samples were injected into the source of the mass spectrometer by a syringe pump with a flow rate of 10 μL/min. Other parameters of the analysis were as follows: capillary voltage 2.6 kV, cone voltage 40 V, source temperature 120 °C, desolvation temperature 400 °C, cone gas 30 L/h, and desolvation gas 600 L/h. The data were collected in full-scan negative-ion mode (mass range of 50–2000 *m*/*z*) and the data processing was performed with Waters MassLynx 4.1 software (deconvolution with MaxEnt1 function, Waters Corporation, Milford, MA, USA). The mass spectra and obtained and found masses of analyzed oligonucleotides are available in the Supplementary Materials (Table S2, Figures S77–S92).

### 2.3. Preparation of 5′-End-Labeled Oligonucleotides

The 40-mer ss oligonucleotides (230 pmol) were 5′-end-labeled using 5 U of polynucleotide kinase T4 (New England BioLabs, Ipswich, MA, USA) with 2 μCi (0.2 μL) [γ-^32^P]ATP (Hartmann Analytic GmbH, Braunschweig, Germany) in 20 μL of buffer (70 mM Tris-HCl, 10 mM MgCl_2_, 5 mM DTT, pH 7.6 at 25 °C) for 30 min at 37 °C. The protein denaturation was obtained by heating the samples to 95 °C for 5 min. Radiograms showing the efficiency of oligo labeling are available in the Supplementary Materials (Figures S1 and S2).

### 2.4. Oligonucleotide Hybridization

The radiolabeled oligonucleotides were hybridized with a 1.2-fold excess of the purified, non-radiolabeled complementary strand in pure H_2_O. After 10 min at 90 °C, the samples were cooled to room temperature for 3–4 h. Obtained duplexes were precipitated with ice-cold ethanol (250 µL) for 30 min and centrifuged (13,000 rpm, 4 °C, 30 min). After ethanol removal, samples were dried under reduced pressure at room temperature. The hybridization efficiency and the purity of dsDNA were examined on 15% denaturing polyacrylamide gel. Radiograms showing the efficiency of oligo labeling and hybridization are available in the Supplementary Materials (Figures S1 and S2).

**Figure 2:**
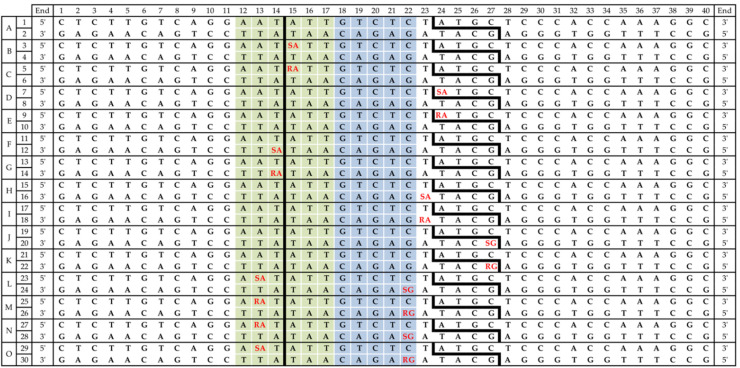
The sequences of substrate oligonucleotides containing 5′,8-cyclo-2′-deoxypurines (cdPus). SX—(5′S)-5′8-cyclo-2′-deoxyadenosine; RX—(5′R)-5′8-cyclo-2′-deoxyadenosine; SY—(5′S)-5′8-cyclo-2′-deoxyguanosine; RY—(5′R)-5′8-cyclo-2′-deoxyguanosine.

Each dsDNA fragment was prepared two times to obtain two different samples with opposing strands radiolabeled. A list of 15 duplexes (A–O) with indicated radiolabeled strands (1–30) is available in Figure 2.

### 2.5. BsmAI and SspI Cleavage Assay

BsmAI and SspI were purchased from NEB (New England BioLabs, Ipswich, MA, USA). The radiolabeled ds oligos (2.3 pmol) were incubated in 5 µL of reaction buffer (50 mM potassium acetate, 20 mM Tris-acetate, 10 mM magnesium acetate, 100 µg/mL BSA) with 0.5 U BsmAI at 55 °C or 1.5 U SspI at 37 °C for each reaction time (0, 1, 5, 15, 30, 45, and 60 min.). The cleavage of each strand was observed. To ensure no incisions of ssDNA were made by REs, control cleavage assays for both enzymes were also performed. All 30 radiolabeled ds oligonucleotides (Figure 2) were treated with SspI and BsmAI separately for 0 and 60 min (Figures S3–S6). Native control assays for strands 1 and 2 (with no lesions) were performed for the following reaction times: 0, 1, 5, 15, 30, and 60 min (Figures S7–S11).

### 2.6. PAGE Electrophoresis

The reactions were stopped by placing the samples in an ice/water bath and adding 7 µL of denaturing loading dye (95% formamide, 2 mM EDTA, 0.025% bromophenol blue, 0.025% xylene cyanol). The samples were analyzed for 120 min at 45 W, on 15% denaturing polyacrylamide gel containing 8 M urea in 1x TBE (89 mM Tris-HCl, 89 mM boric acid, 2 mM EDTA). The results were visualized by autoradiography and quantified using Quantity One 1-D analysis software (Bio-Rad). All experiments were performed three times to ensure repetitive and consistent data. To obtain a percentage value of DNA cleavage, the intensity of each band was calculated as a percentage of the total intensity of all bands within one lane.

### 2.7. Data Presentation

In the case of each strand and enzyme, values of DNA cleavage for 60 min were taken into account, unless otherwise indicated. If the strand cleavage value is given in relation to the control, it means that the final cleavage values (after 60 min) for both strands are compared. Complete raw numerical data is shown in Tables S3 and S4. A summary figure containing the complete sequences of oligonucleotides and cleavage values after 60 min is available in Supplementary Materials (Figure S93).

## 3. Results and Discussion

In the present study, the influence of single- and bi-stranded CDLs on the activity of BsmAI and SspI were examined. The radiolabeled ds oligonucleotides containing cdPus (ScdA, RcdA, ScdG, or RcdG) were treated with both enzymes separately (see Materials and Methods). Lesions in substrate ds oligos were located within or outside restriction and/or cleavage sites. Double-stranded DNA fragments were designed to show the cleavage efficiency of each strand (duplexes A–O, Figure 2). The designated numbers from 1 to 30 indicate which strand of each duplex was examined. The leading strands are marked with odd numbers, while even numbers indicate complementary strands. Duplex A (strands 1 and 2) was used as a native control without any lesions in its sequence.

As a result of dsDNA cleavage by REs, the cleavage sites were observed as a set of additional bands on a radiogram, the intact strand (40-mer) and cleaved strand, which differed in length because of different incision sites. For SspI, 14-mer and 26-mer products were obtained after incision of the leading and complementary strands, respectively. BsmAI created 23-mer and 13-mer products after incision of the leading and complementary strands, respectively. Control assays for BsmAI showed 99% cleavage (strand 1) and 98% cleavage (strand 2) after 60 min; the cleavage efficiency for SspI was 86% for strand 1 and 82% for strand 2 (Figures S7–S11). The individual radiograms and graphs for the discussed assays are presented in the Supplementary Materials (Figures S12–S76).

### 3.1. Influence of 5′S and 5′R cdPus on SspI Activity

The SspI incision site is located within its recognition site. First, the impact of lesions located within the recognition site was considered. The distance in bp between the incision site of the enzyme and the lesion varied and were as follows: +1 toward the 3′-end of the leading strand (strands 3 and 5), −1 toward the 3′-end of the complementary strand (strands 12 and 14), and –2 toward the 5′-end of the leading strand (strands 23, 25, 27, and 29). For all substrates, only one row of bands representing intact dsDNA was observed (Figures S17, S27, S37, S47, S57 and S67). Therefore, it can be concluded that the presence of examined lesions within the recognition site fully inhibits the activity of SspI.

**Figure 3:**
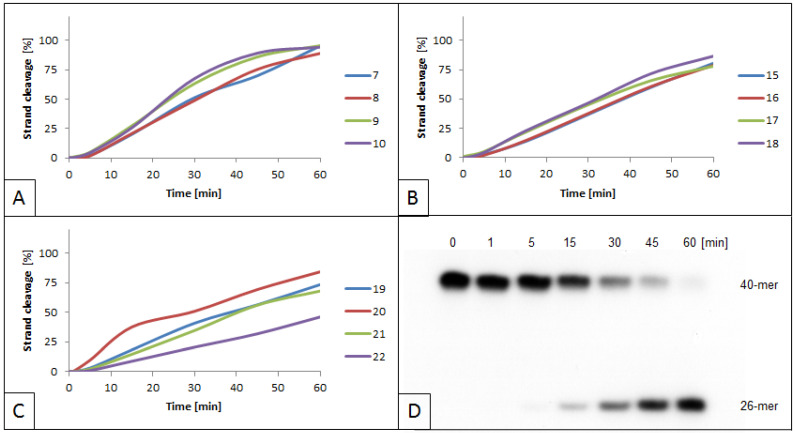
Cleavage of dsDNA containing cdPus outside the SspI recognition site by 1.5 U SspI. (**A**) dsDNA containing cdA on the leading strands (quantity increase of cleaved strands 7–10); (**B**) dsDNA containing cdA on complementary strands (quantity increase of cleaved strands 15–18); (**C**) dsDNA containing cdG on complementary strands (quantity increase of cleaved strands 19–22); (**D**) representative autoradiogram of cleaved strand 8 containing no lesions. Strand 8 is the complementary strand of duplex D, which contains ScdA on the leading strand (strand 7).

Lesions located outside the recognition site were also evaluated. The distance between the incision site of the enzyme and the lesion varied and were as follows: +10 toward the 3′-end of the leading strand (strands 7 and 9, Figures S17, S20, S27 and S30), +9 toward the 5′-end of the complementary strand (strands 16 and 18, Figures S37, S41, S47 and S51), and +13 toward the 5′-end of the complementary strand (strands 20 and 22, Figures S67–S69). For all investigated oligonucleotides, the activity of the SspI was higher than that of the control (duplex A, Figures S7, S10 and S11). For example, 50% of cleavage was obtained after 20–30 min for strands 7–10, compared with 30–35 min for the control (Figures S7–S9). Nevertheless, in most cases, the inhibition of the enzyme’s activity was unrelated to the lesion type (Figure 3). Comparing two pairs of duplexes (D vs. E and H vs. I) there was no notable change in the maximum cleavage values (from 89 to 99% for strands 7–10 and from 78 to 86% for strands 15–18). The only exception was strand 22 of duplex K, containing RcdG in the complementary strand. In that case, the enzyme’s activity was 38% lower than that in duplex J, containing ScdG at the same position (strand 20). The change in the lesion position on the enzyme’s activity was also evaluated. Duplexes with ScdA or RcdA at position 24 of the leading strand (strands 7–10, Figure 3A) were completely cleaved (89–96% vs. 82–86% for control), while for strands 15–18 (ScdA/RcdA at position 23 of the complementary strand, Figure 3B) and 19–22 (ScdG/RcdG at position 27 of the complementary strand, Figure 3C), the efficiency of cleavage decreased (78–86% and 46–84%, respectively). RcdG distanced 10 bp from the restriction site toward the 5′-end of the complementary strands inhibited the enzyme’s ability to cleave the DNA to 68% for strand 21 and to 46% for strand 22.

### 3.2. Influence of 5′S and 5′R cdA on BsmAI activity

**Figure 4:**
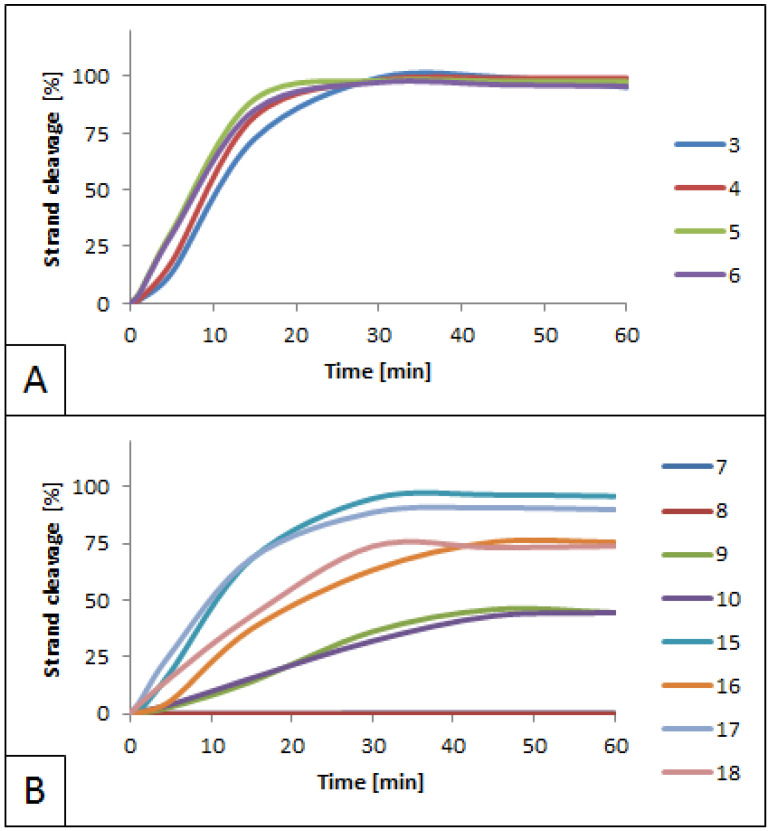
Cleavage of dsDNA containing ScdA and RcdA by 0.5 U BsmAI. (**A**) Quantity increase of cleaved strands 3–6. These strands are arranged in duplexes B and C, which contain cdA lesions located within the recognition site of SspI (Figure 2). (**B**) Quantity increase of cleaved strands 7–18. These strands are arranged in duplexes D–E and H–I, which contain cdA lesions located within the cleavage site of BsmAI (Figure 2).

BsmAI activity was evaluated in the context of cdA presence in the dsDNA. The enzyme’s cleavage site is located outside the recognition site, and the cleavage of both strands occurs in different positions. This allows the investigation of BsmAI activity in detail using dsDNA fragments containing lesions within both DNA strands. When cdA was located outside the recognition and incision sites, the enzyme activity for strands 3–6 and 11–14 was similar (50% of cleavage in less than 15 min) when compared with native strands (50% of cleavage achieved in approximately 10 min for duplex A). Moreover, these results were comparable for 5′*S* and 5′*R* diastereomers (Figure 4A, strands 3–6 and 11–14; Figures S32–S34 and S42–S44). This indicated that cdA, regardless of its diastereomeric form, located 3–4 bp away from the recognition site, did not affect the activity of BsmAI, as expected.

In the case of lesions adjacent to the cleavage site of the enzyme (ScdA in position 24 toward the 3′ of the leading strands, strands 7–8, Figures S12, S15 and S16) the activity of BsmAI was blocked and lowered to 45% for strands 9–10 containing RcdA (Figure 4B, Figures S22, S25 and S26). Interestingly, this is in opposition to the trend observed in other studies on cdPus, where RcdA lowered incision efficiency of hAPE1 to a greater extent than ScdA [13]. The 1 nucleotide shift in the lesion’s position (position 23 toward the 5′-end of the complementary strand, strands 15–18, Figures S32, S35, S36, S42, S45 and S46) abolished the enzyme’s inhibition. However, in the case of strands 15–18, complementary strands containing a lesion (16 and 18) were cleaved slower than leading native strands (15 and 17). This is in agreement with the fact that cdPus affects the spatial structure of dsDNA and, therefore, may impede proper DNA-enzyme interactions, leading to its slower action. Moreover, the oligonucleotide containing RcdA (strand 18) was cleaved faster than the one containing ScdA (strand 16). The maximum cleavage for strand 18 reached 74% after 30 min, while for strand 16 it took 45 min to reach the same level (Figure 4B).

### 3.3. Influence of 5′S and 5′R cdG on BsmAI Activity

**Figure 5:**
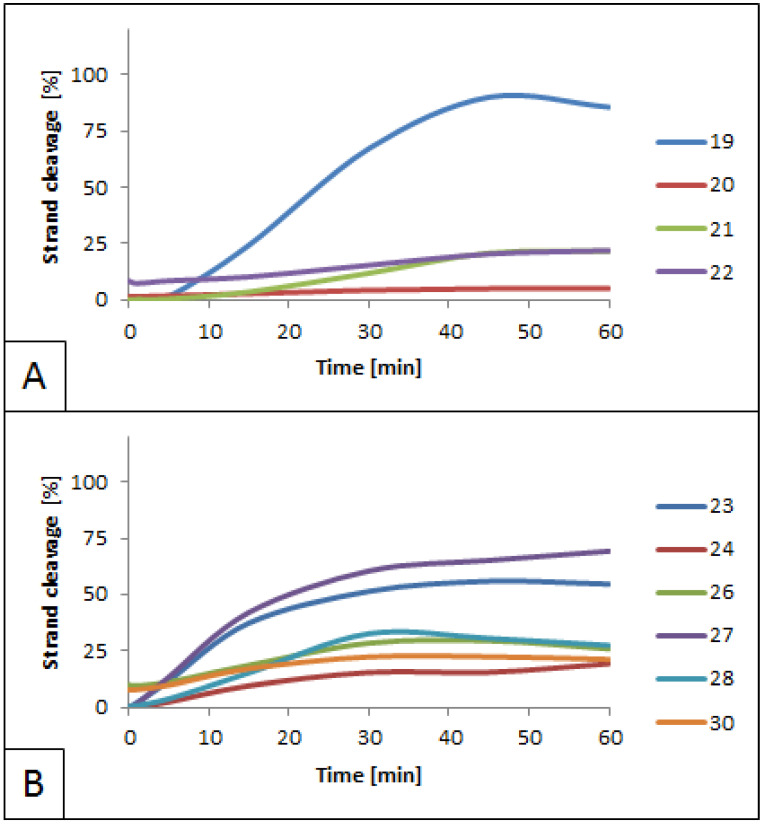
Cleavage of dsDNA containing ScdG and RcdG by 0.5 U BsmAI. (**A**) Quantity increase of cleaved strands 19–22. These strands are arranged in duplexes J and K, which contain cdG lesions located within the cleavage site of BsmAI (Figure 2). (**B**) Quantity increase of cleaved strands 23–24, 26–28, and 30. These strands are arranged in duplexes L–O, which contain double-stranded lesions—cdPus are located on both strands, within the recognition sites of both enzymes (Figure 2).

The influence of cdG on BsmAI activity was also evaluated (Figures S62–S64 and S72–S74). Cleavage of the strand with no lesions (strand 19) was slightly lower when compared with the control (85% cleavage after 60 min). At the same time, the enzyme’s activity was blocked for the strand containing the lesion (strand 20). In the case of RcdG, the enzyme’s activity was 76% lower for strands 21 and 22 than the control (Figure 5A). This indicates that in the case of cdG, the 5′*S* diastereomer blocks the activity of BsmAI more than 5′*R*, which is in agreement with the trend observed for cdA. The ability of the enzyme to cleave the leading undamaged strand of duplex J (strand 19) may appear due to the greater distance between the lesion (ScdG) and the recognition site of the enzyme. In the case of duplex D containing ScdA, the distance was shorter, and the enzyme remained inactive.

In the case of dsDNA containing lesions in both strands, ScdG and RcdG showed a lower impact on the activity of BsmAI when present within the recognition site (20–28% cleavage for strands 24, 26, 28, and 30, Figure 5B, Figures S52–S56, S62, S65 and S66) comparing with the incision site (5–22% cleavage for strands 20 and 22, Figure 5A). Taking into consideration that corresponding duplexes for strands 20 and 22 (duplexes J and K) have no additional lesions on the opposite strands, it might be assumed that additional lesions in duplexes L–O have no impact on BsmAI activity. Moreover, the difference between the lowest and the highest values of the strand cleavage for strands 24, 26, 28, and 30 was 8% after 60 min. This indicates that there is no notable connection between the type of lesion and its ability to inhibit BsmAI if the lesion is located within the enzyme’s recognition site.

## 4. Conclusions

Numerous studies have indicated the biological significance of CDLs and cdPus [5,31,32,33]. However, there were no prior findings concerning their influence on the mechanism of RE action on the DNA. In this study, for the first time, the influence of cdPus on the activity of SspI and BsmAI was investigated. These two enzymes were chosen as representatives of type II REs, which are the most commonly used molecular biology tools. The obtained results led to the following conclusions:

- The activity of SspI was blocked by cdPus located within the enzyme’s recognition site.
- The inhibition of SspI by lesions placed outside the enzyme’s recognition site was inversely proportional to the distance between the lesions and the recognition site.
- The BsmAI activity was similar for all cdPus placed outside the enzyme’s recognition and incision sites—in most cases, the enzyme’s activity was slightly lower or similar to that in the control.
- The inhibition of BsmAI was lower for RcdA than for ScdA when lesions were located adjacent to the incision site of the enzyme—the results were similar for lesions located in different positions (position 24 toward the 3′-end of the leading strand and position 23 toward the 5′-end of the complementary strand).
- The presence of cdG (both diastereomers) next to the incision site of BsmAI lowered its activity more than cdA (both diastereomers).
- CdG showed a lower impact on the activity of BsmAI when it was located within the recognition site than next to the incision site.

Restriction endonucleases play an extremely important role in bacterial defense against foreign genetic material. Therefore, agents affecting their activity have become a point of interest. This study showed the potential of cdPus to diminish RE activity. Since it is known that cdPus may affect eukaryotic DNA repair [11,13,14], it is advisable to investigate the impact of cdPus on the maintenance of the bacterial genome in more detail. Moreover, current findings on the influence of cdPus on eukaryotic DNA repair are based on research performed in vitro or using pure enzymes [13,14]. Studies on bacterial models may lead to a further in vivo approach to unveil the mechanism of cdPus action [34,35,36,37]. Finally, REs play a crucial role in bacterial genome maintenance and have become widely used as a tool for genetic engineering [27,38]. Thus, in the context of this study, defining their mechanism of action and recruitment to the recognition sites may help to uncover the details of impairments created by cdPus.

## Data Availability

Not applicable.

## Supplementary Materials

The following are available online, Table S1: Complete sequences of applied oligonucleotides, Table S2: Calculated and found molecular masses of applied oligonucleotides, Figure S1: Efficiency of oligo labeling and hybridization for duplexes A-I (strands 1-18), Figure S2: Efficiency of oligo labeling and hybridization for duplexes J-O (strands 19-30), Figure S3: Control cleavage assay for BsmAI–strands 1–18, Figure S4: Control cleavage assays for SspI–strands 1–18, Figure S5: Control cleavage assays for BsmAI–strands 19–30, Figure S6: Control cleavage assays for SspI–strands 19-30, Figure S7: Autoradiograms of native control assays for BsmAI and SspI, Figures S8–S9: Cleavage of native control strands by BsmAI, Figures S10–S11: Cleavage of native control strands by SspI, Figures S12–S76: Autoradiograms and graphs of assays for BsmAI and SspI, Figures S77–S92: Mass spectra of applied oligonucleotides, Table S3: The influence of cdPus on BsmAI activity–raw numerical data, Table S4: The influence of cdPus on SspI activity–raw numerical data, Figure S93: The sequence of substrate oligonucleotides containing cdPus.

## Sample Availability

Samples of the compounds are not available from the authors.
