# Supplementary material for: The Influence of 5′R and 5′S cdA and cdG on the Activity of BsmAI and SspI Restriction Enzymes
Source: Molecules. 2021 Jun 19;26(12):3750. doi: 10.3390/molecules26123750 (PMC8234751; doi:10.3390/molecules26123750)
Supplement: Supplementary file 1 [file molecules-26-03750-s001.zip › molecules-1259708-SI.pdf]

The influence of 5'*R* and 5'*S* cdA and cdG on the activity of BsmAI and SspI restriction enzymes

Supplementary Materials

Table of contents

Page	Item	Description
2	Table S1	Complete sequences of applied oligonucleotides
2	Table S2	Calculated and found molecular masses of applied oligonucleotides
3	Figure S1	Efficiency of oligo labeling and hybridization for duplexes A-I (strands 1-18)
3	Figure S2	Efficiency of oligo labeling and hybridization for duplexes J-O (strands 19-30)
4	Figure S3	Control cleavage assay for BsmAI – strands 1-18
4	Figure S4	Control cleavage assays for SspI – strands 1-18
5	Figure S5	Control cleavage assays for BsmAI – strands 19-30
5	Figure S6	Control cleavage assays for SspI – strands 19-30
6	Figures S7	Autoradiograms of native control assays for BsmAI and SspI
7	Figures S8-S9	Cleavage of native control strands by BsmAI
8	Figures S10-S11	Cleavage of native control strands by SspI
9-47	Figures S12-S76	Autoradiograms and graphs of assays for BsmAI and SspI
48-62	Figures S77-S92	Mass spectra of applied oligonucleotides
63	Table S3	The influence of cdPus on BsmAI activity – raw numerical data
67	Table S4	The influence of cdPus on SspI activity – raw numerical data
71	Figure S93	The sequence of substrate oligonucleotides containing cdPus.

Table S1. Complete sequences of applied oligonucleotides. Abbreviations mean the following: SX - (5'S)-5'8-cyclo-2'-deoxyadenosine; RX - (5'R)-5'8-cyclo-2'-deoxyadenosine; SY - (5'S)-5'8-cyclo-2'-deoxyguanosine; RY - (5'R)-5'8-cyclo-2'-deoxyguanosine.

Oligo	End		End
Matrix 5'	5'	CTCTTGTCAGGAATATTGTCTCTATGCTCCCACCAAAGGC	3'
Matrix 3'	3'	GAGAACAGTCCTTATAACAGAGATACGAGGGTGGTTTCCG	5'
Sequence 1	5'	CTCTTGTCAGGAAT**SX**TTGTCTCTATGCTCCCACCAAAGGC	3'
Sequence 2	5'	CTCTTGTCAGGAAT**RX**TTGTCTCTATGCTCCCACCAAAGGC	3'
Sequence 3	5'	CTCTTGTCAGGAATATTGTCTCT**SX**TGCTCCCACCAAAGGC	3'
Sequence 4	5'	CTCTTGTCAGGAATATTGTCTCT**RX**TGCTCCCACCAAAGGC	3'
Sequence 5	3'	GAGAACAGTCCTT**SX**TAACAGAGATACGAGGGTGGTTTCCG	5'
Sequence 6	3'	GAGAACAGTCCTT**RX**TAACAGAGATACGAGGGTGGTTTCCG	5'
Sequence 7	3'	GAGAACAGTCCTTATAACAGAG**SX**TACGAGGGTGGTTTCCG	5'
Sequence 8	3'	GAGAACAGTCCTTATAACAGAG**RX**TACGAGGGTGGTTTCCG	5'
Sequence 9	3'	GAGAACAGTCCTTATAACAGAGATAC**SY**AGGGTGGTTTCCG	5'
Sequence 10	3'	GAGAACAGTCCTTATAACAGAGATAC**RY**AGGGTGGTTTCCG	5'
Sequence 11	5'	CTCTTGTCAGGA**SX**TATTGTCTCTATGCTCCCACCAAAGGC	3'
Sequence 12	3'	GAGAACAGTCCTTATAACAGA**SY**ATACGAGGGTGGTTTCCG	5'
Sequence 13	5'	CTCTTGTCAGGARXTATTGTCTCTATGCTCCCACCAAAGGC	3'
Sequence 14	3'	GAGAACAGTCCTTATAACAGA**RY**ATACGAGGGTGGTTTCCG	5'

Table S2. Calculated and found molecular masses [Da] of applied oligonucleotides.

Oligonucleotide	Calculated mass	Found mass
Matrix 5'	12182.00	12182.42
Matrix 3'	12409.10	12409.82
Sequence 1	12181.00	12180.10
Sequence 2	12181.00	12180.20
Sequence 3	12181.00	12180.10
Sequence 4	12181.00	12180.20
Sequence 5	12408.10	12407.30
Sequence 6	12408.10	12407.30
Sequence 7	12408.10	12407.30
Sequence 8	12408.10	12407.30
Sequence 9	12408.10	12407.30
Sequence 10	12408.10	12407.50
Sequence 11	12182.00	12180.20
Sequence 12	12408.10	12407.20
Sequence 13	12182.00	12180.10
Sequence 14	12408.10	12407.40

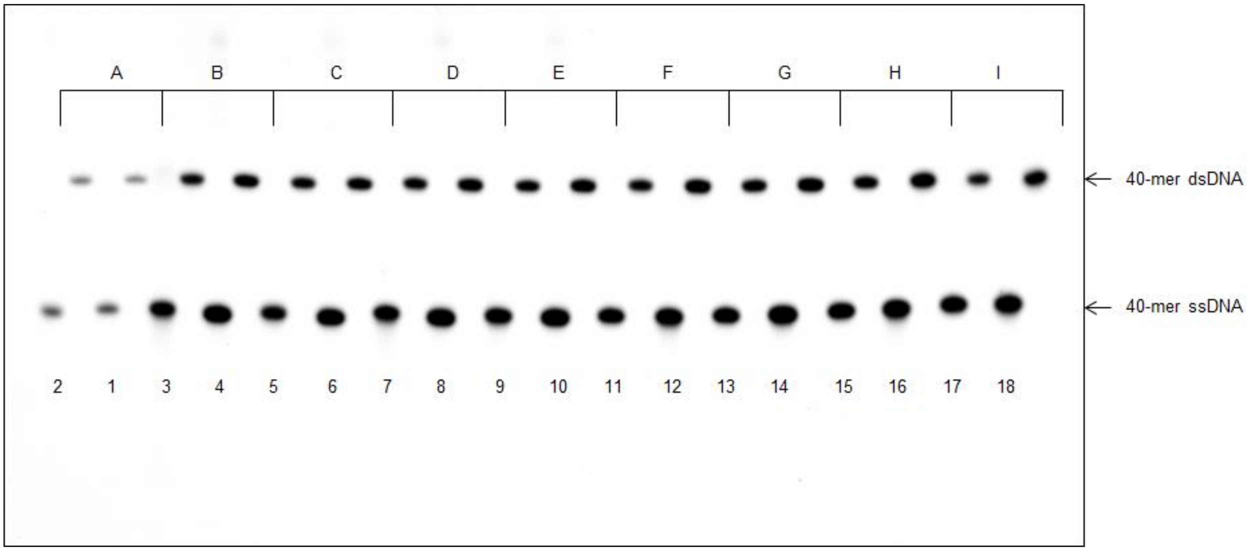

Figure S1. Visualization of efficient labeling (ssDNA) and hybridization (dsDNA) of oligonucleotides. Labeled strands 1-18 and obtained duplexes A-I are shown.

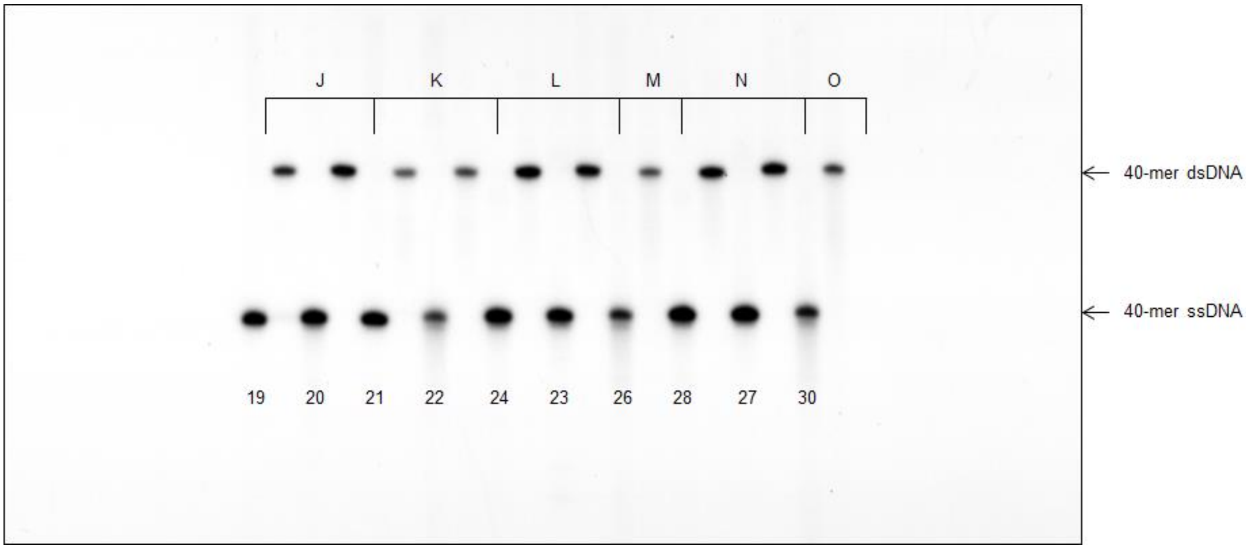

Figure S2. Visualization of efficient labeling (ssDNA) and hybridization (dsDNA) of oligonucleotides. Labeled strands 19-30 and obtained duplexes J-O are shown.

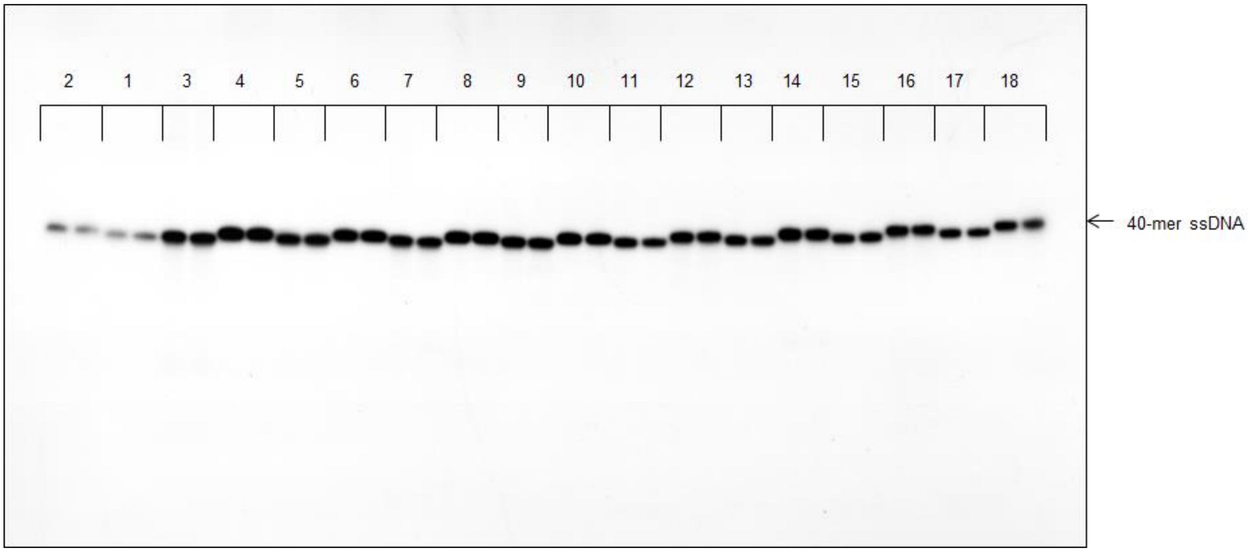

Figure S3. Control cleavage assay: cleavage of ssDNA (strands 1-18) by 0.5 U BsmAI for 0 min (left band) and 60 min (right band).

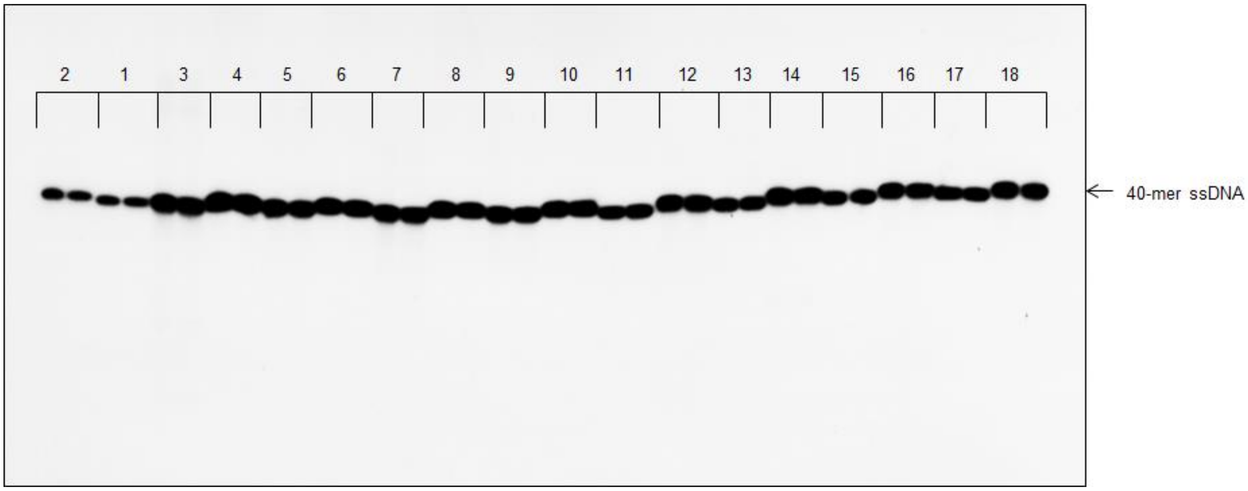

Figure S4. Control cleavage assay: cleavage of ssDNA (strands 1-18) by 1.5 U SspI for 0 min (left band) and 60 min (right band).

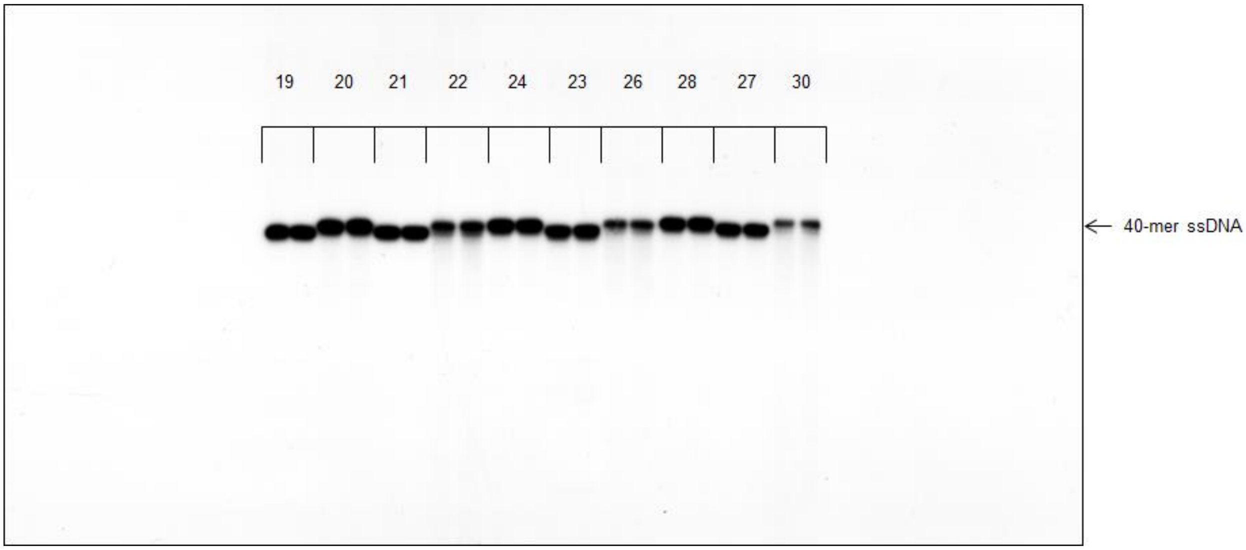

Figure S5. Control cleavage assay: cleavage of ssDNA (strands 19-30) by 0.5 U BsmAI for 0 min (left band) and 60 min (right band).

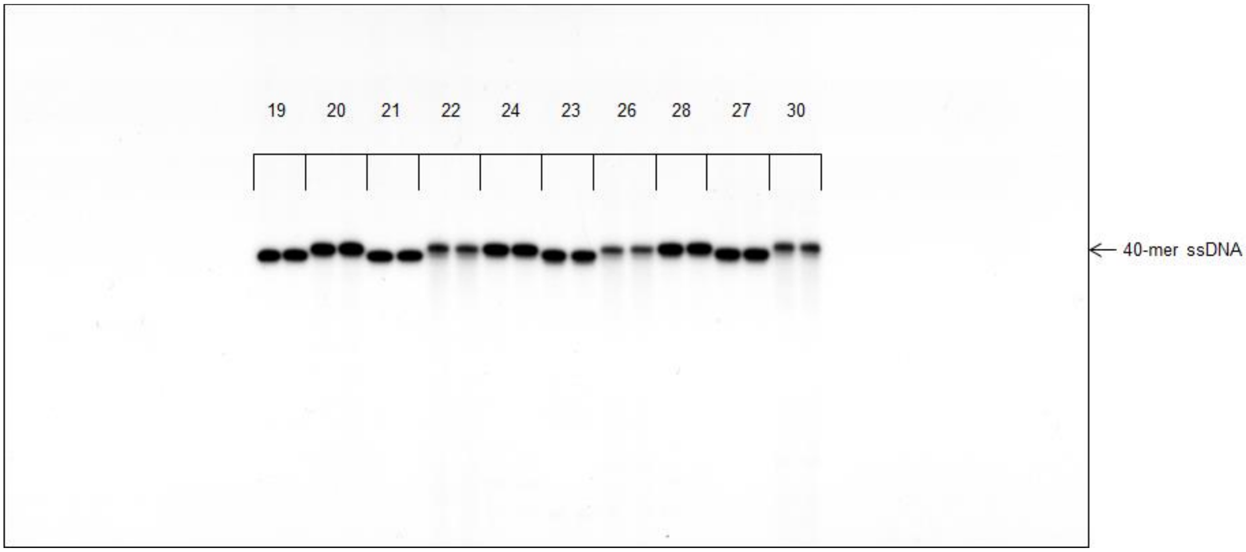

Figure S6. Control cleavage assay: cleavage of ssDNA (strands 19-30) by 1.5 U SspI for 0 min (left band) and 60 min (right band).

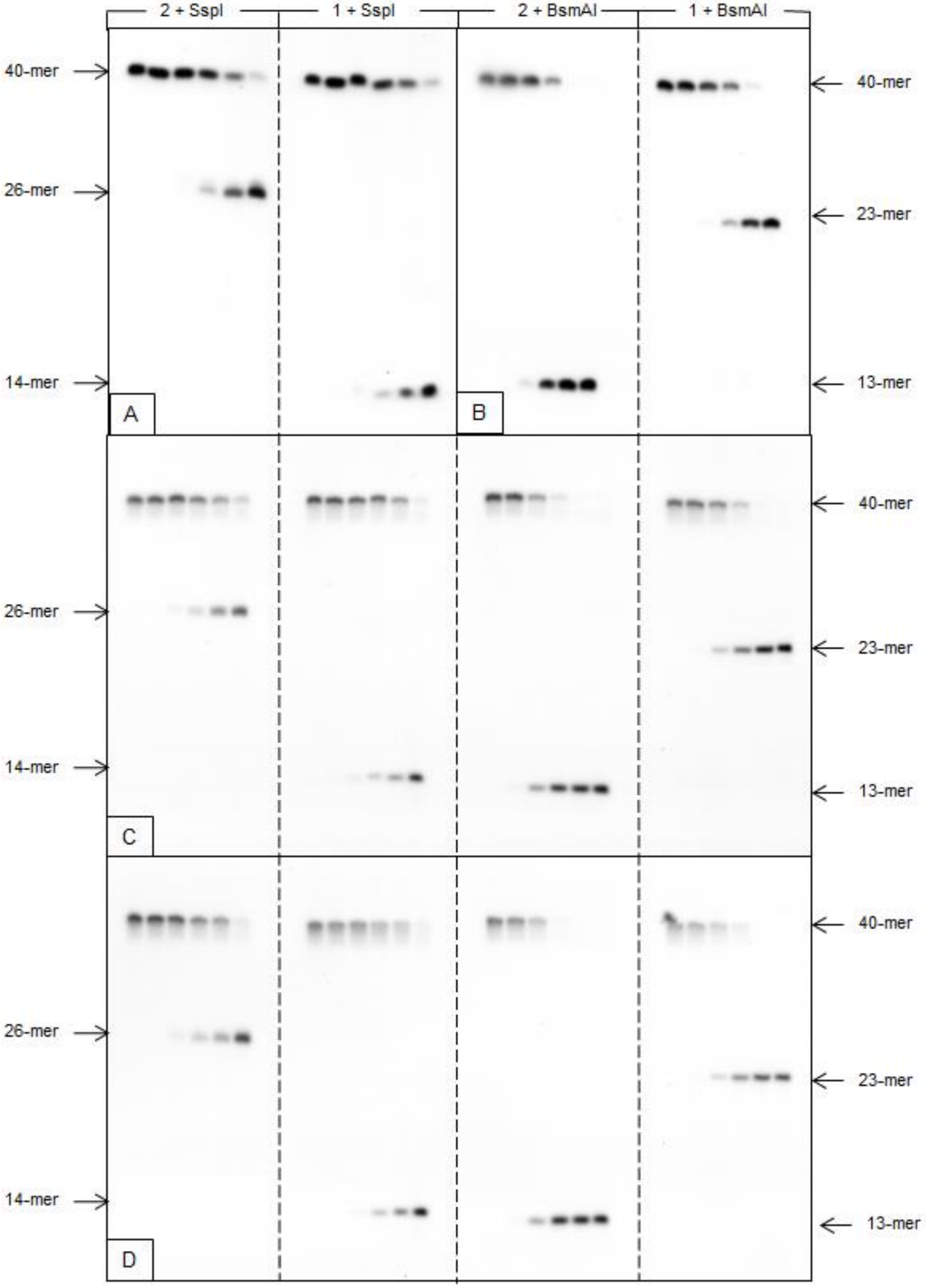

Figure S7. Native control assays for strands 1 and 2. Lanes of bands represent following reaction times: 0, 1, 5, 15, 30 and 60 min starting from the left. (A) autoradiogram of repeat 1 for SspI; (B) autoradiogram of

repeat 1 for BsmAI; (C) autoradiogram of repeat 2 for SspI and BsmaI; (D) autoradiogram of repeat 3 for SspI and BsmaI.

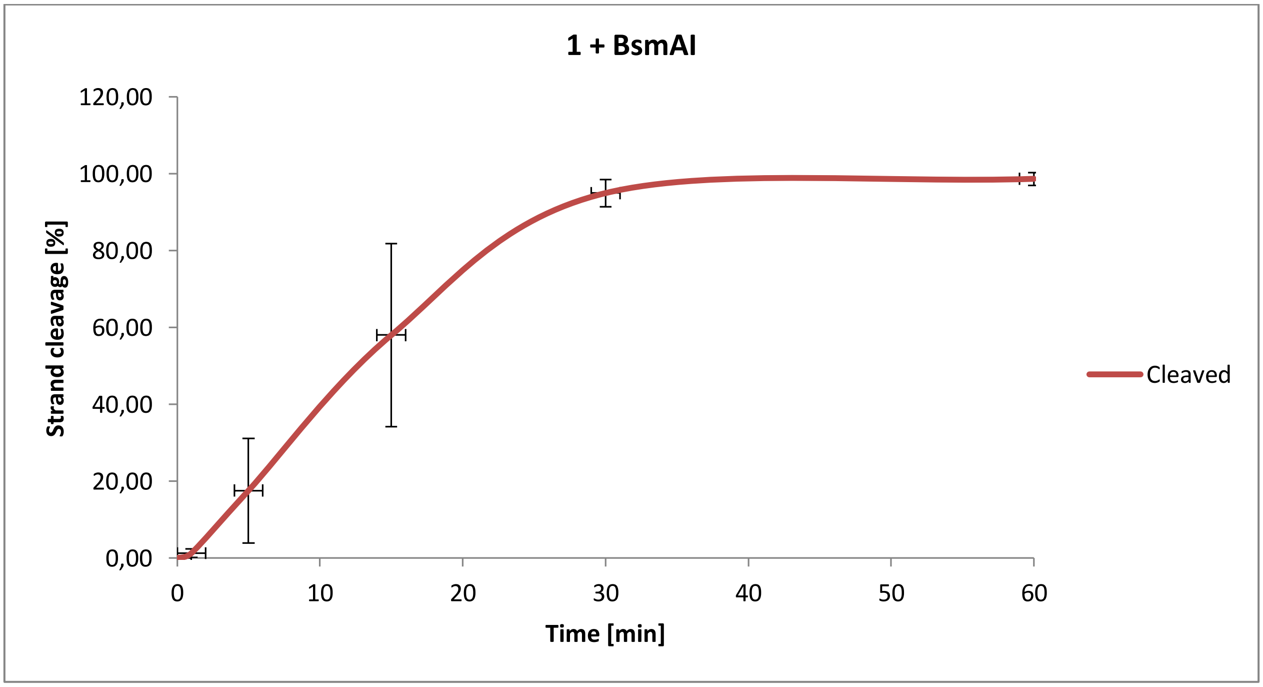

Figure S8. Cleavage of native control dsDNA (duplex A) by 0.5 U BsmAI. Quantity increase of cleaved ssDNA (strand 1) is shown.

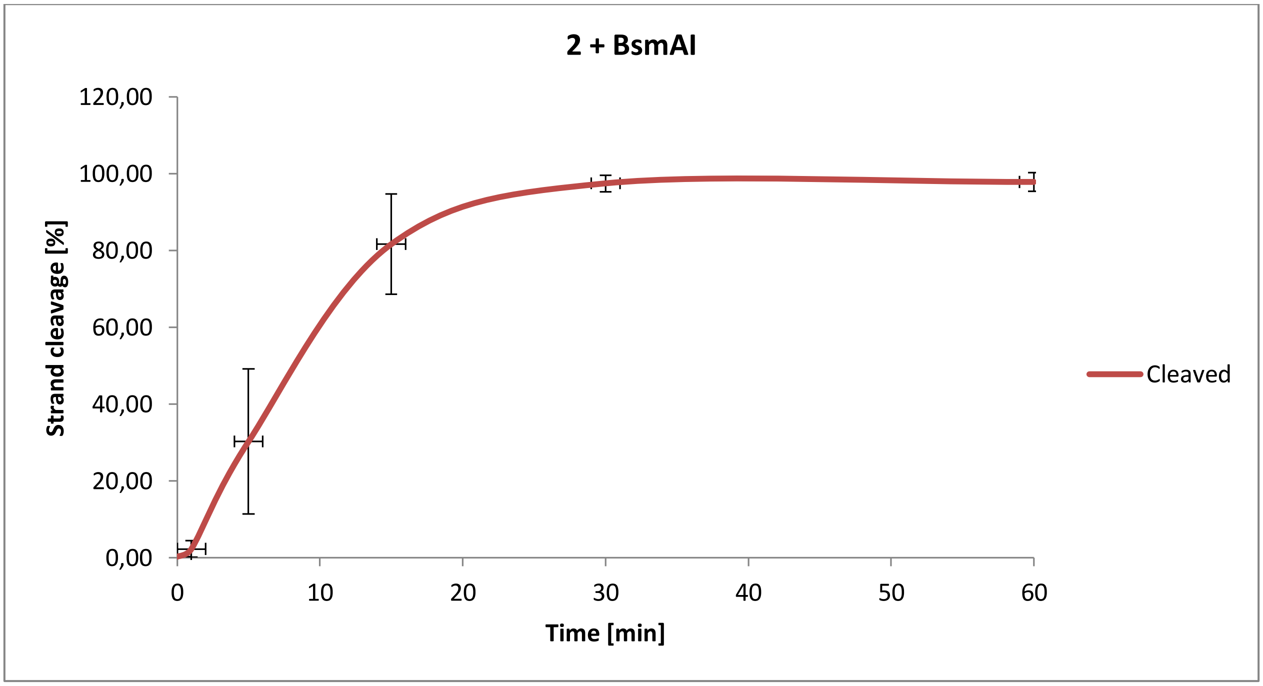

Figure S9. Cleavage of native control dsDNA (duplex A) by 0.5 U BsmAI. Quantity increase of cleaved ssDNA (strand 2) is shown.

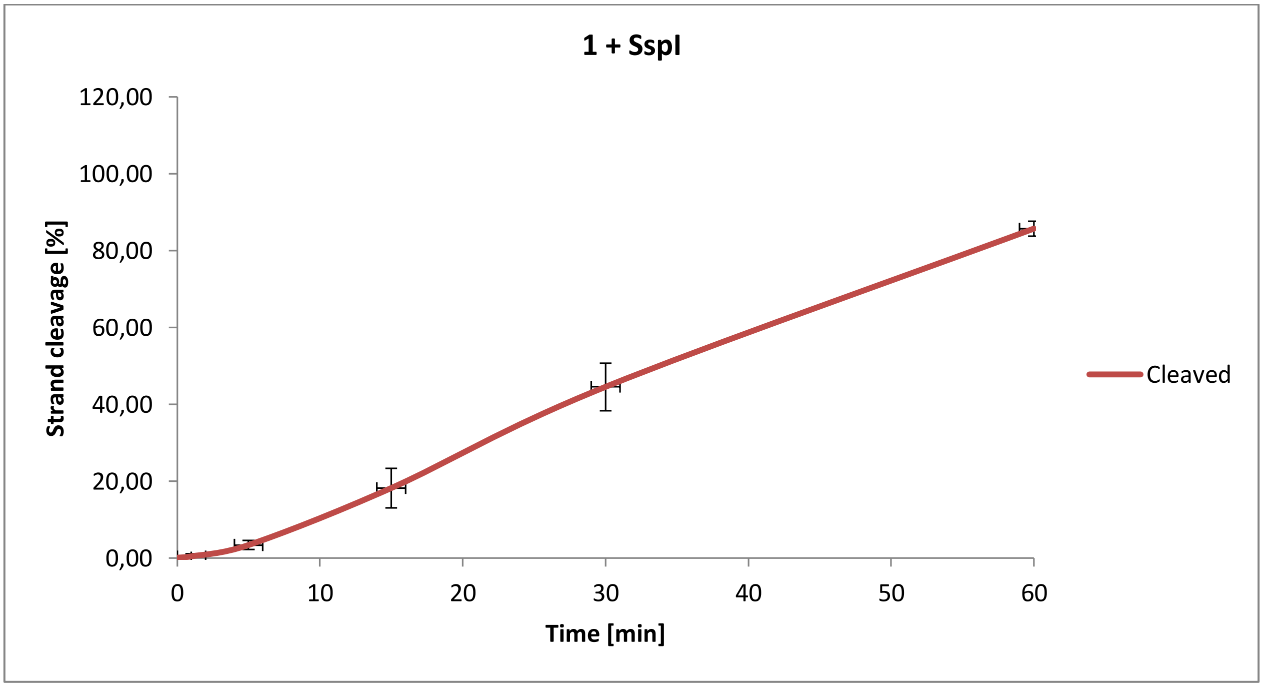

Figure S10. Cleavage of native control dsDNA (duplex A) by 1.5 U SspI. Quantity increase of cleaved ssDNA (strand 1) is shown.

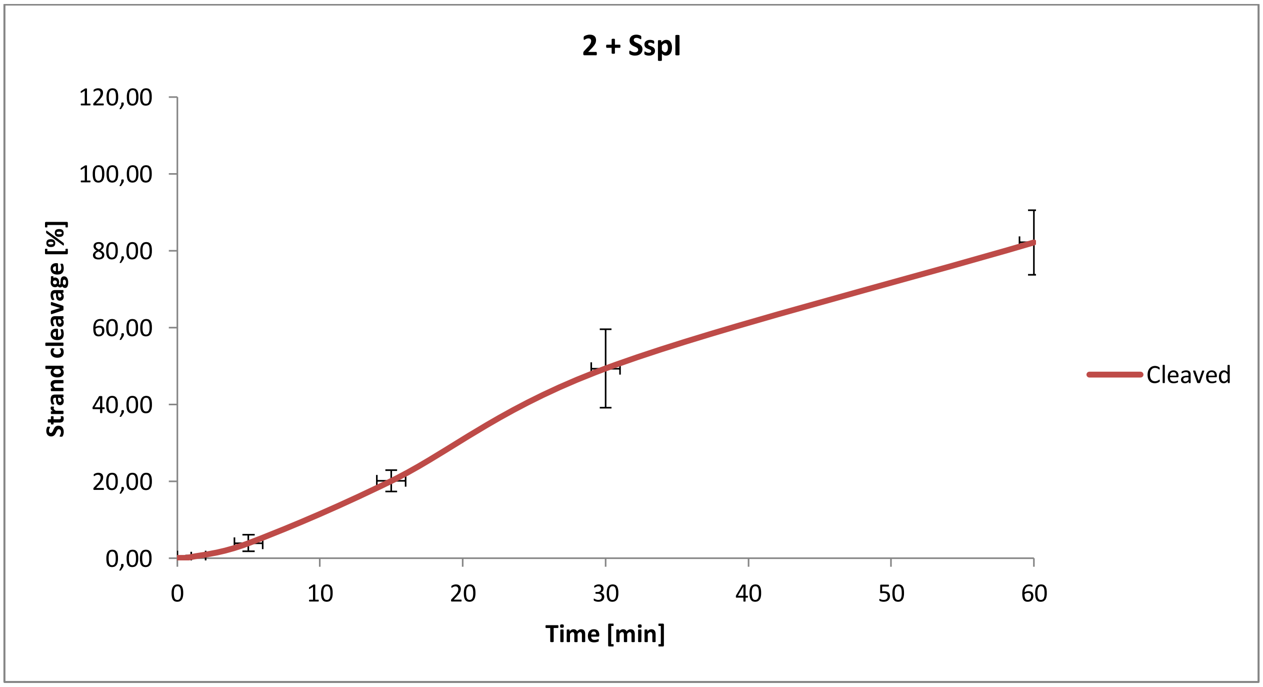

Figure S11. Cleavage of native control dsDNA (duplex A) by 1.5 U SspI. Quantity increase of cleaved ssDNA (strand 2) is shown.

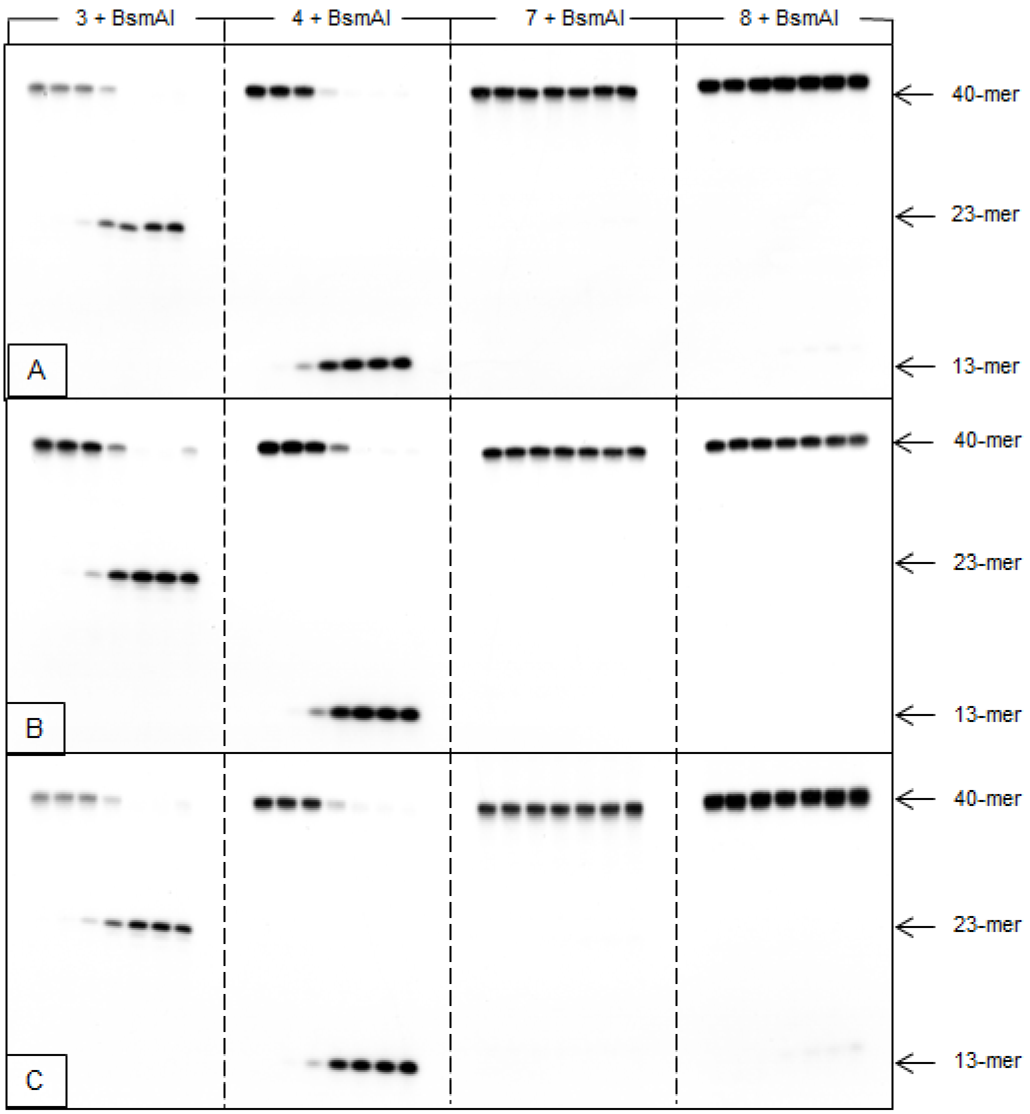

Figure S12. The autoradiograms of denaturing PAGE presenting cleavage of oligonucleotides by 0.5 U BsmAI – (A), (B) and (C) represent three repeats of assays. Lanes of bands represent following reaction times: 0, 1, 5, 15, 30, 45 and 60 min starting from the left.

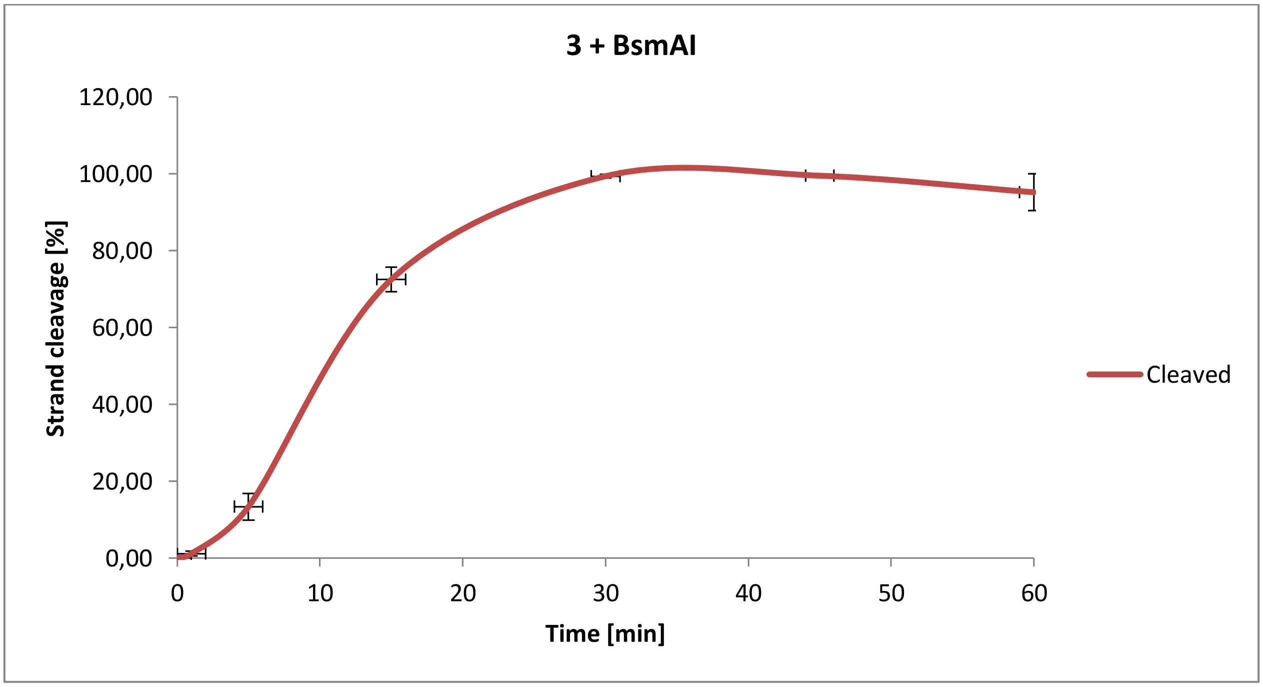

Figure S13. Cleavage of dsDNA (duplex B) by 0.5 U BsmAI. Quantity increase of cleaved ssDNA (strand 3) is shown.

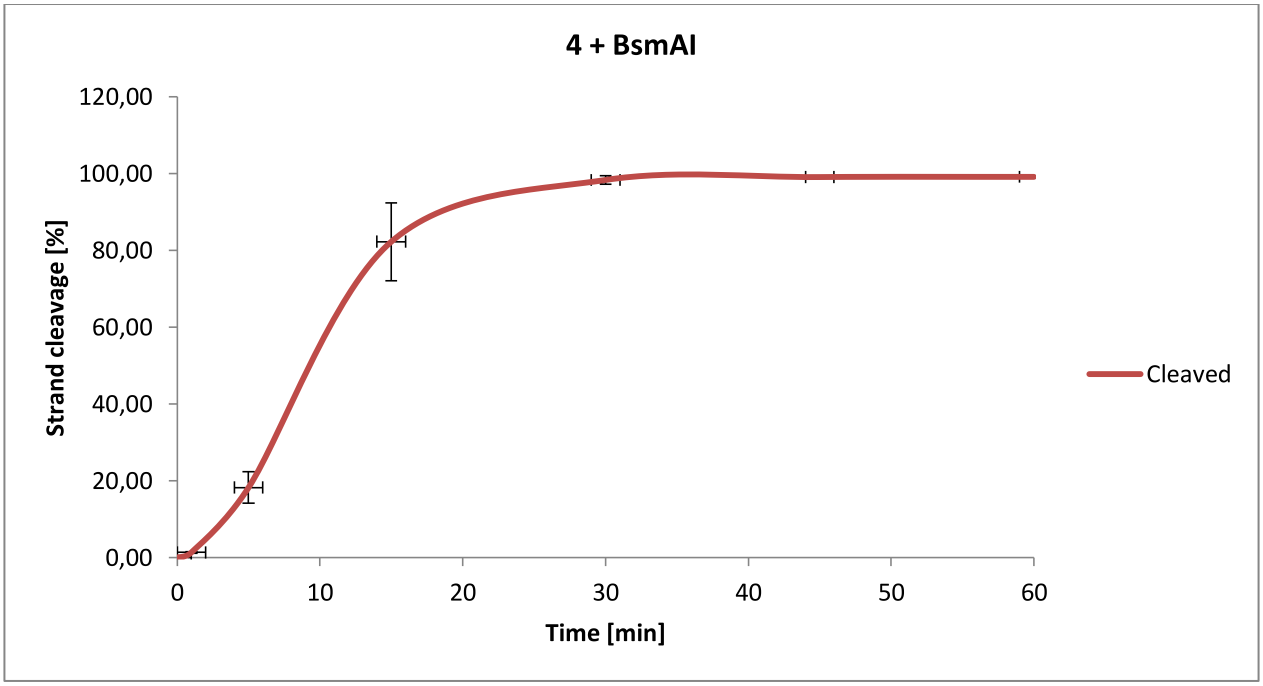

Figure S14. Cleavage of dsDNA (duplex B) by 0.5 U BsmAI. Quantity increase of cleaved ssDNA (strand 4) is shown.

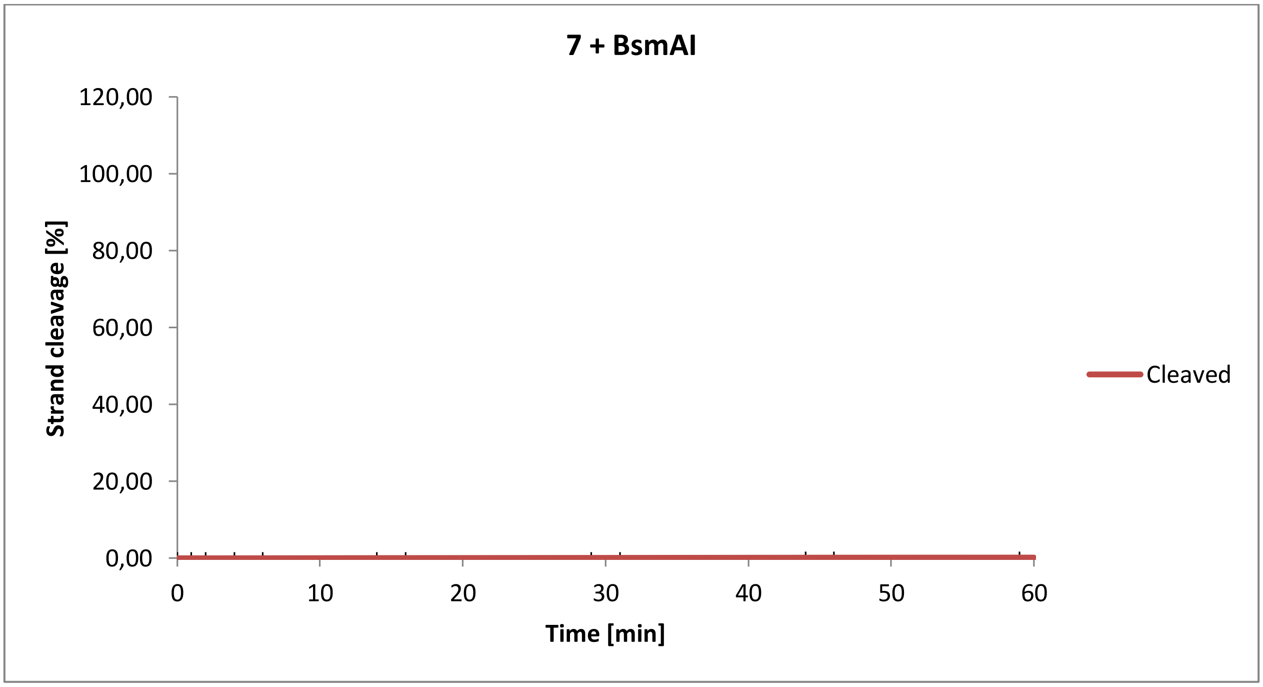

Figure S15. Cleavage of dsDNA (duplex D) by 0.5 U BsmAI. Quantity increase of cleaved ssDNA (strand 7) is shown.

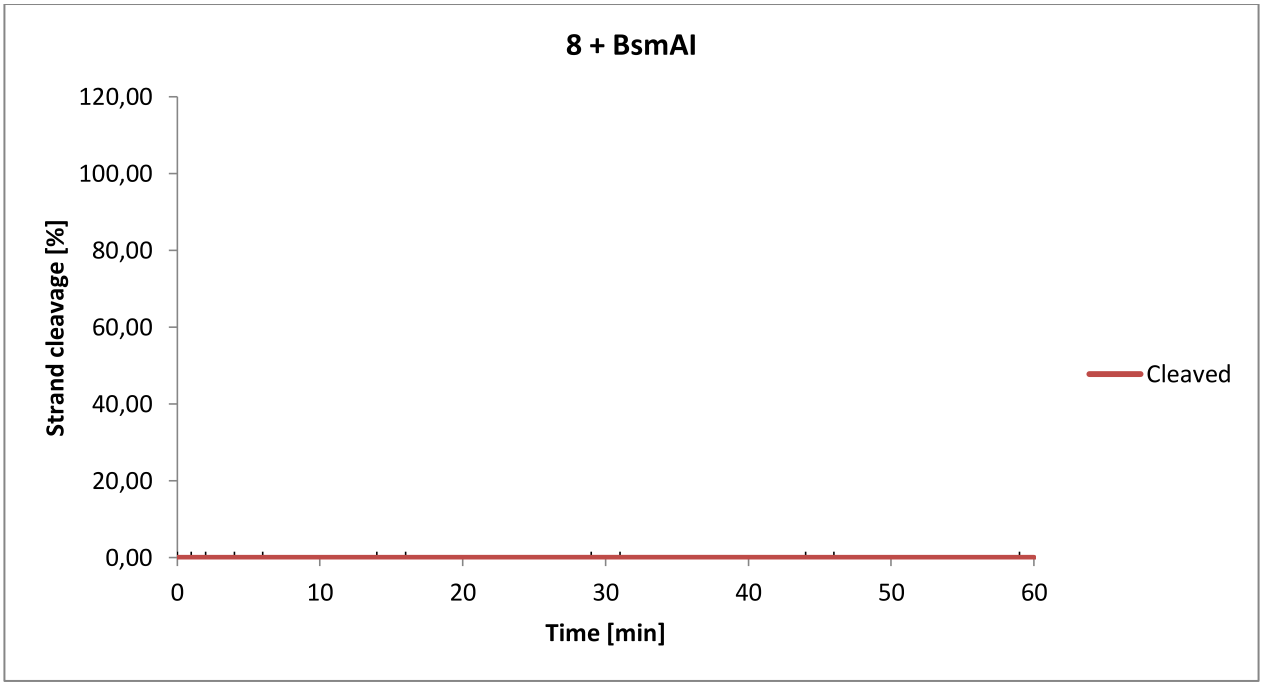

Figure S16. Cleavage of dsDNA (duplex D) by 0.5 U BsmAI. Quantity increase of cleaved ssDNA (strand 8) is shown.

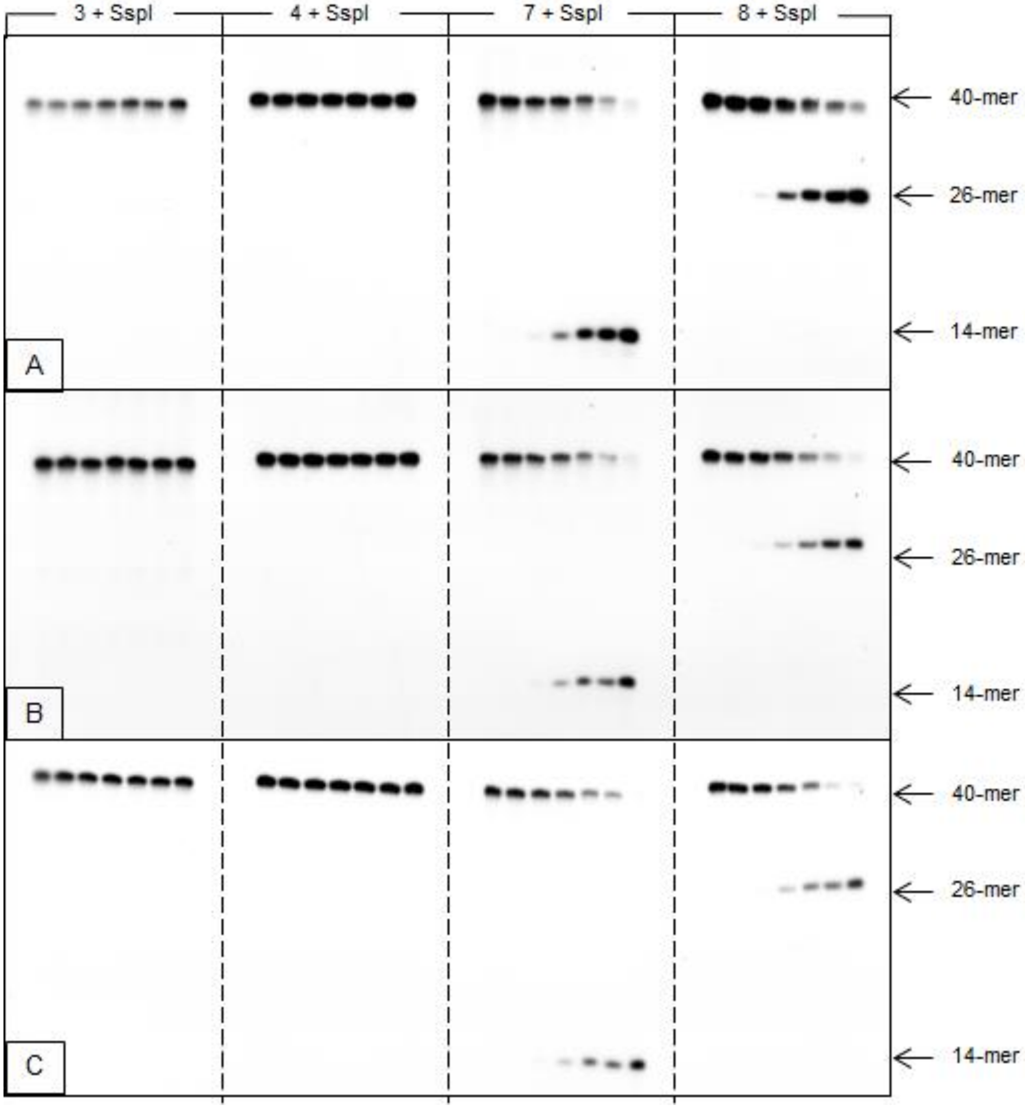

Figure S17. The autoradiograms of denaturing PAGE presenting cleavage of oligonucleotides by 1.5 U SspI – (A), (B) and (C) represent three repeats of assays. Lanes of bands represent following reaction times: 0, 1, 5, 15, 30, 45 and 60 min starting from the left.

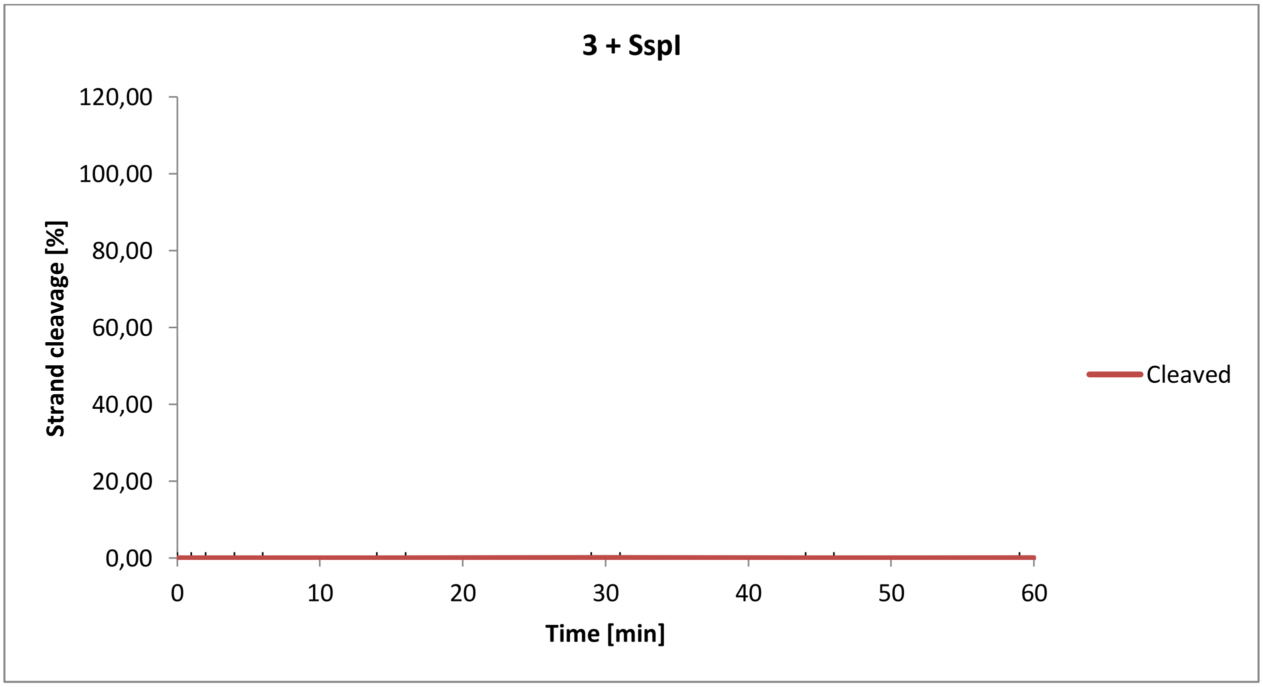

Figure S18. Cleavage of dsDNA (duplex B) by 1.5 U SspI. Quantity increase of cleaved ssDNA (strand 3) is shown.

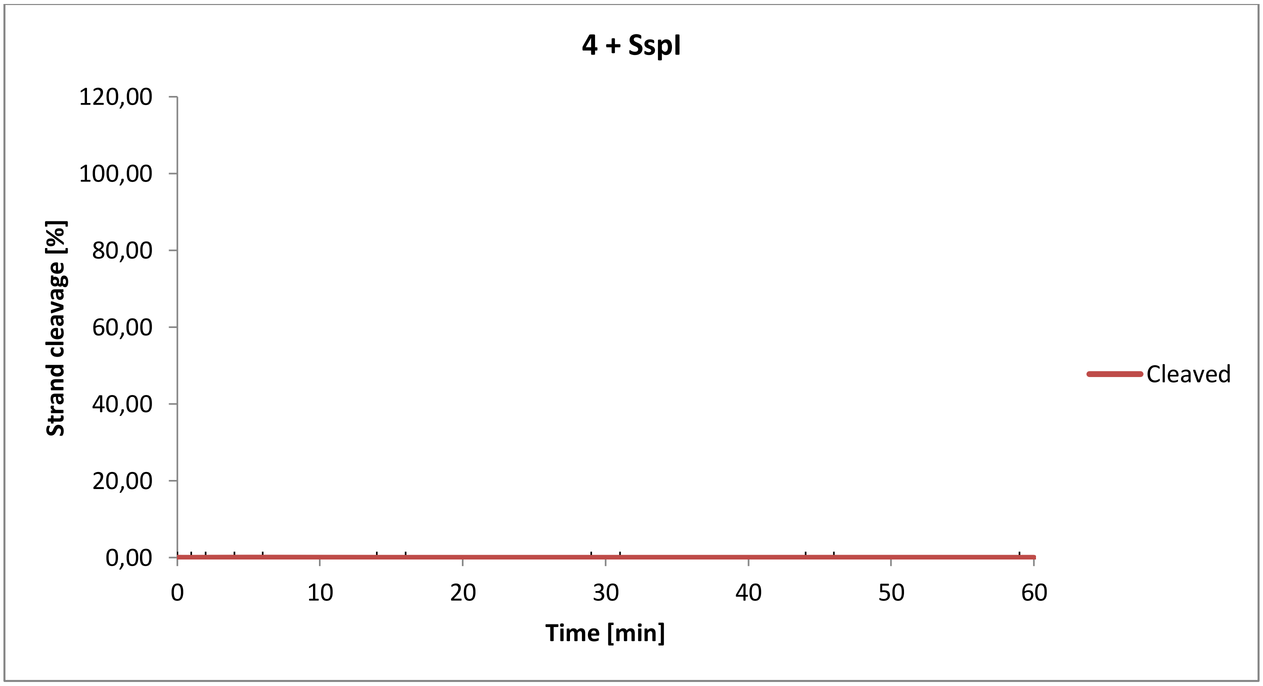

Figure S19. Cleavage of dsDNA (duplex B) by 1.5 U SspI. Quantity increase of cleaved ssDNA (strand 4) is shown.

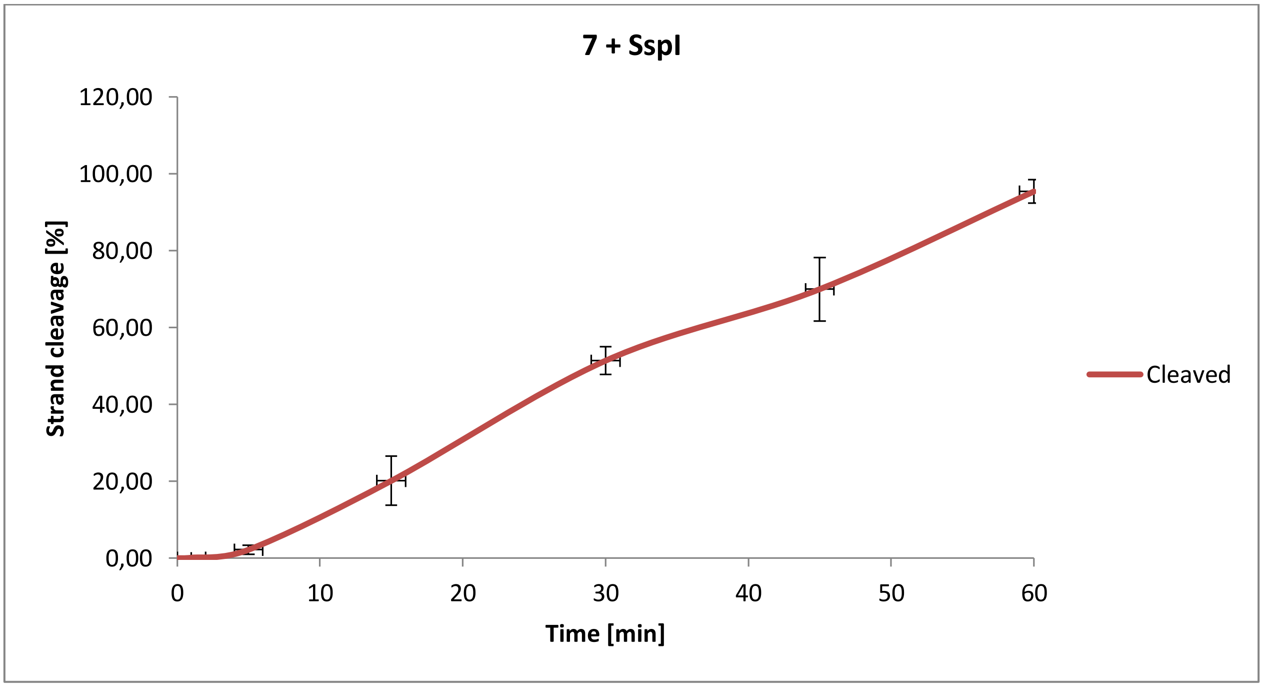

Figure S20. Cleavage of dsDNA (duplex D) by 1.5 U SspI. Quantity increase of cleaved ssDNA (strand 7) is shown.

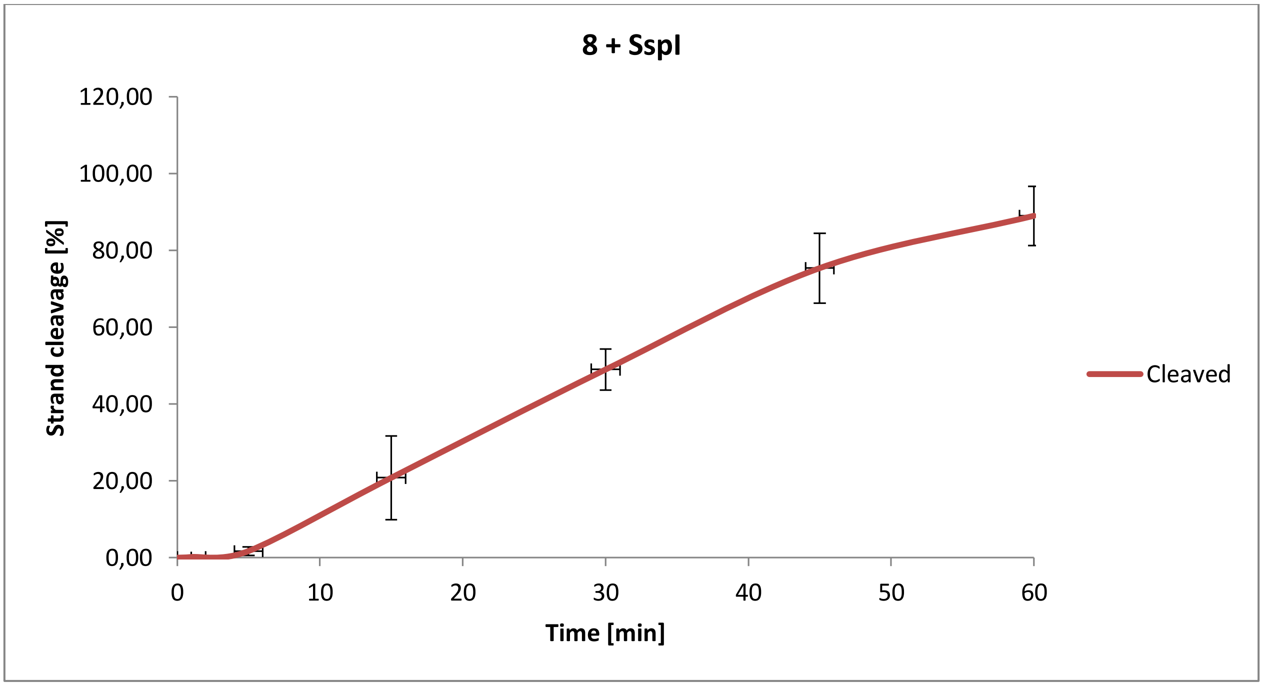

Figure S21. Cleavage of dsDNA (duplex D) by 1.5 U SspI. Quantity increase of cleaved ssDNA (strand 8) is shown.

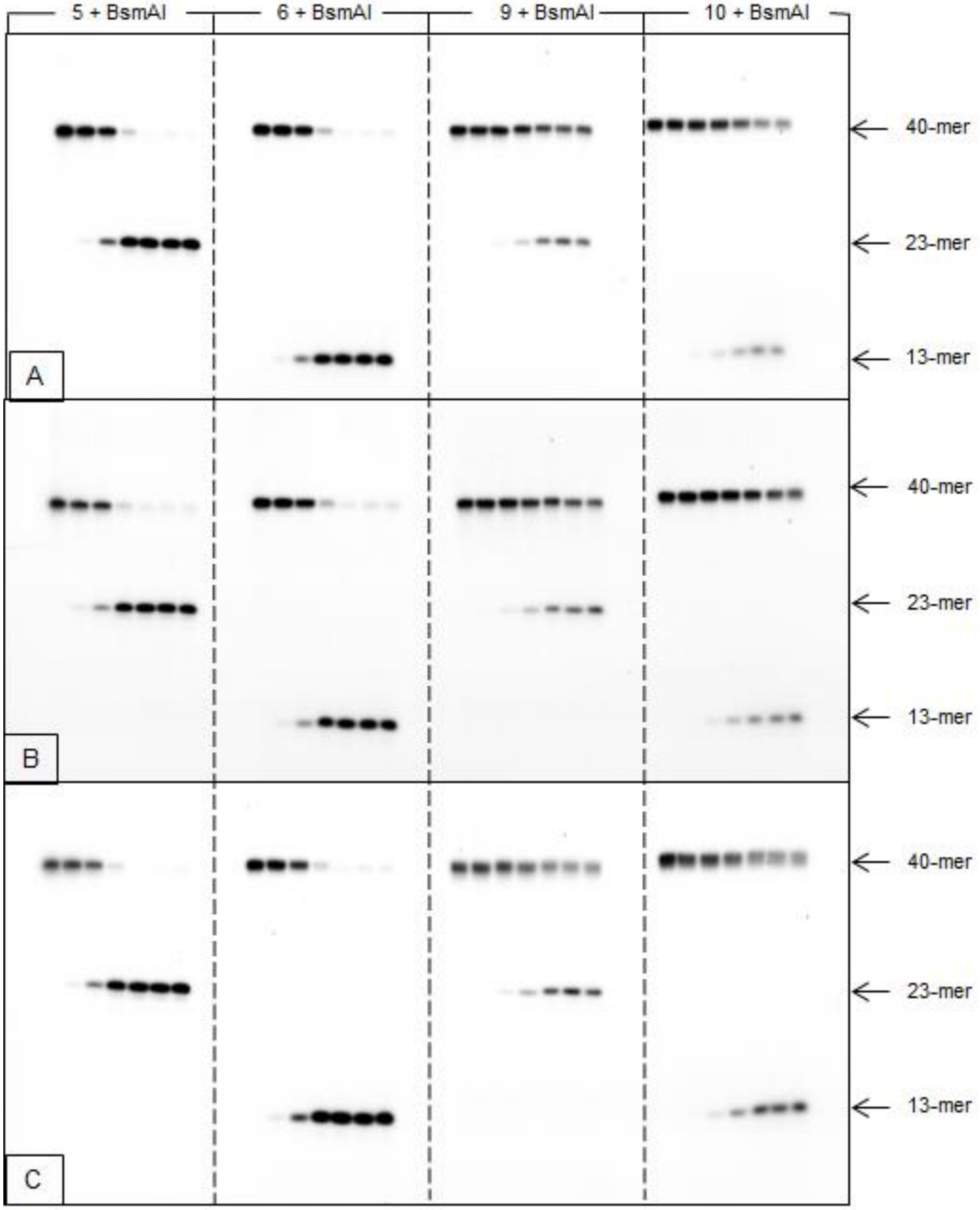

Figure S22. The autoradiograms of denaturing PAGE presenting cleavage of oligonucleotides by 0.5 U BsmAI – (A), (B) and (C) represent three repeats of assays. Lanes of bands represent following reaction times: 0, 1, 5, 15, 30, 45 and 60 min starting from the left.

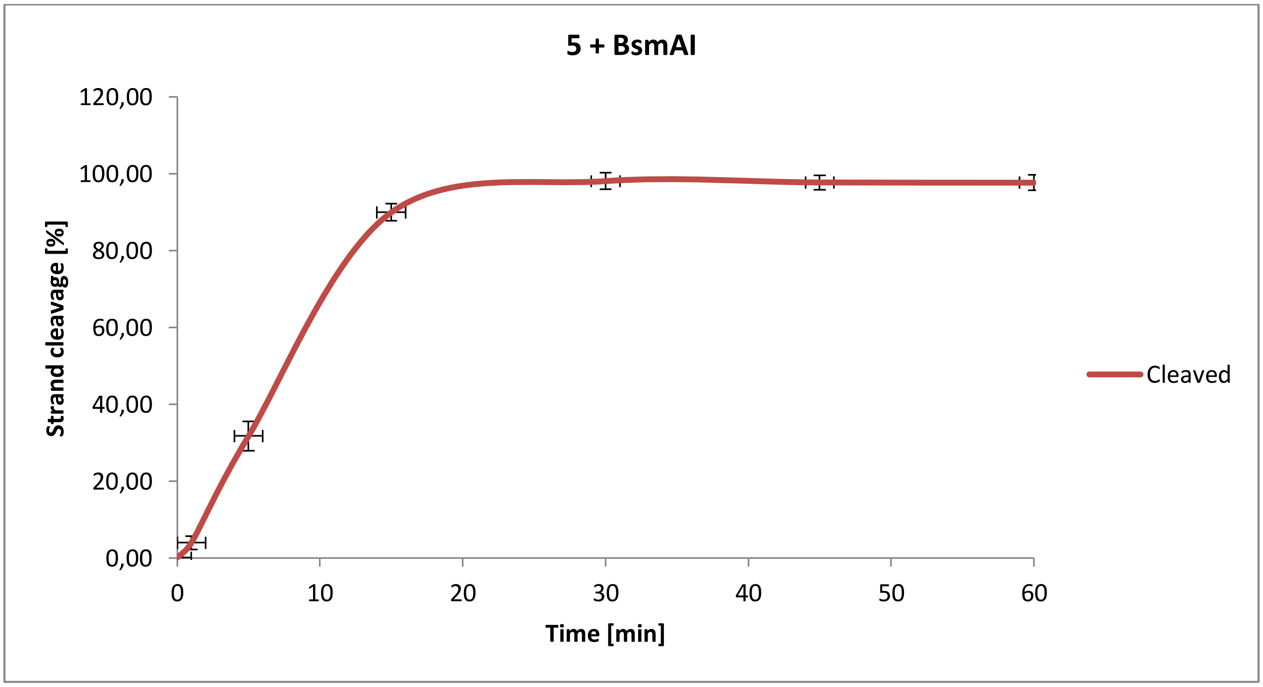

Figure S23. Cleavage of dsDNA (duplex C) by 0.5 U BsmAI. Quantity increase of cleaved ssDNA (strand 5) is shown.

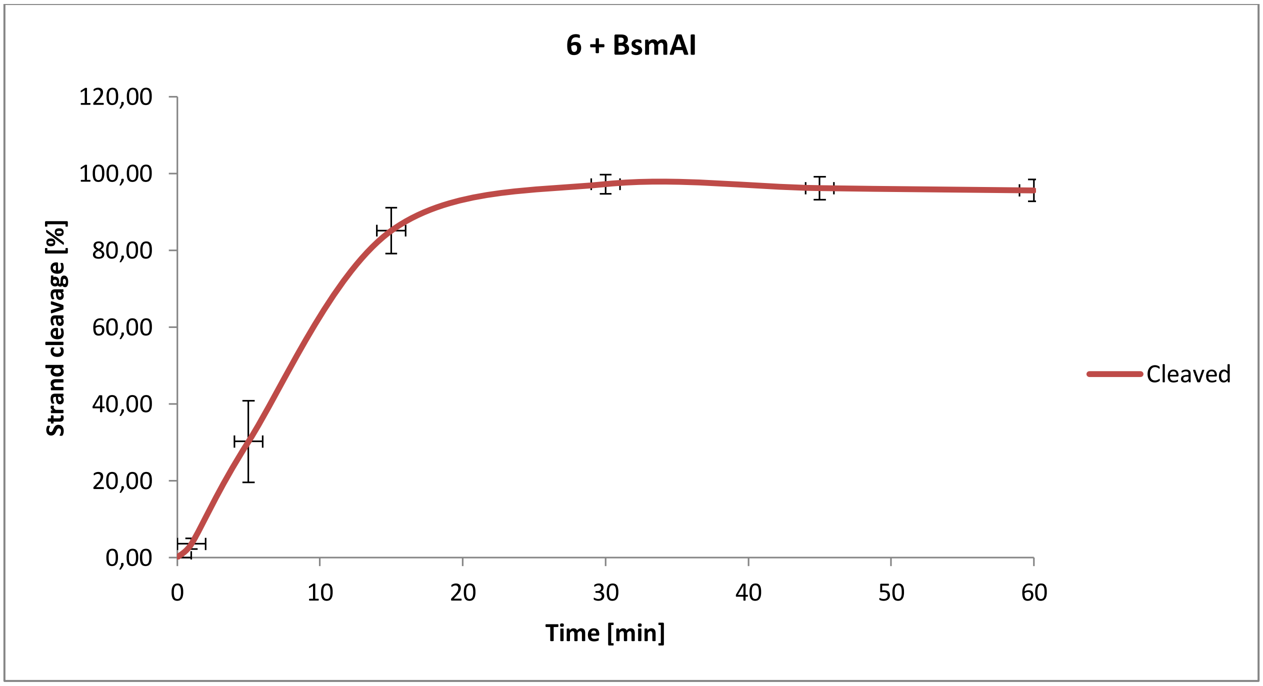

Figure S24. Cleavage of dsDNA (duplex C) by 0.5 U BsmAI. Quantity increase of cleaved ssDNA (strand 6) is shown.

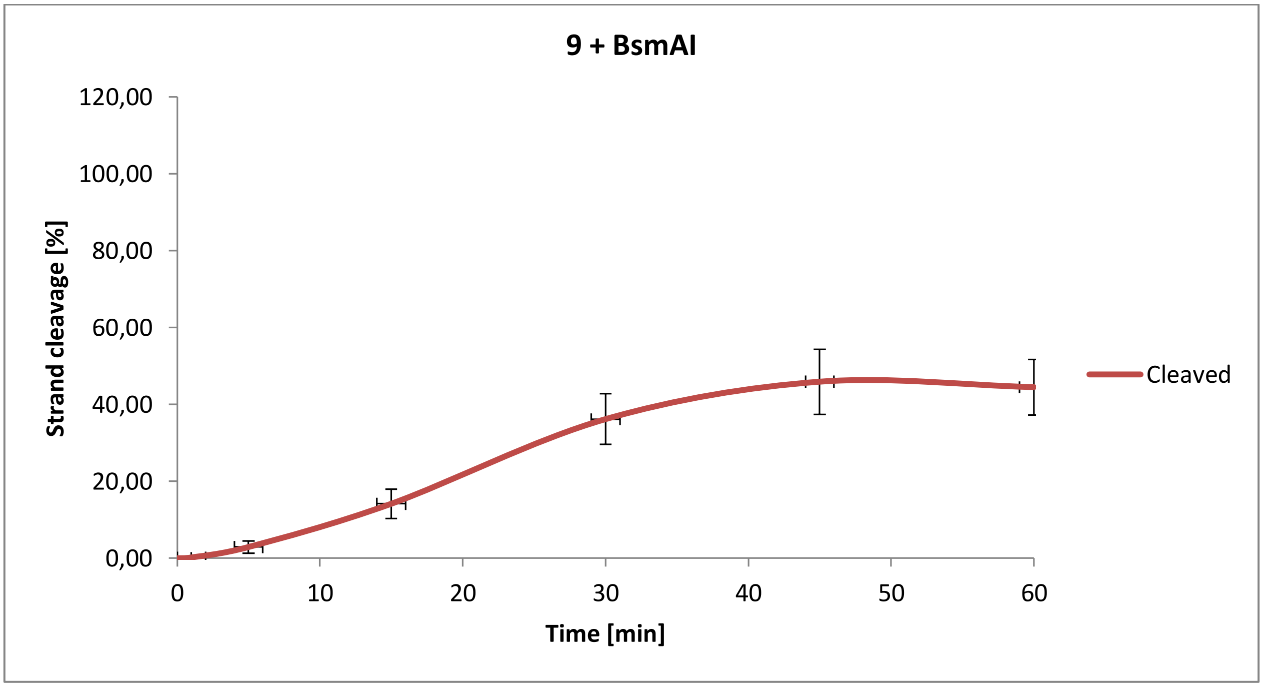

Figure S25. Cleavage of dsDNA (duplex E) by 0.5 U BsmAI. Quantity increase of cleaved ssDNA (strand 9) is shown.

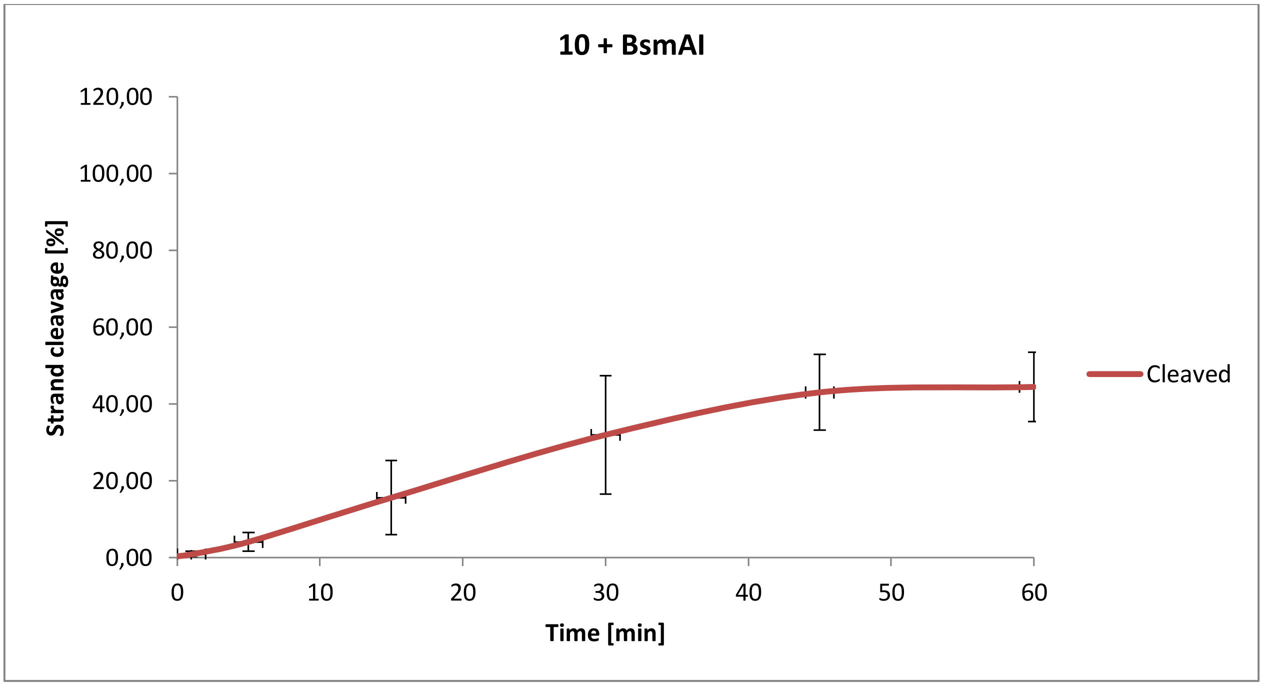

Figure S26. Cleavage of dsDNA (duplex E) by 0.5 U BsmAI. Quantity increase of cleaved ssDNA (strand 10) is shown.

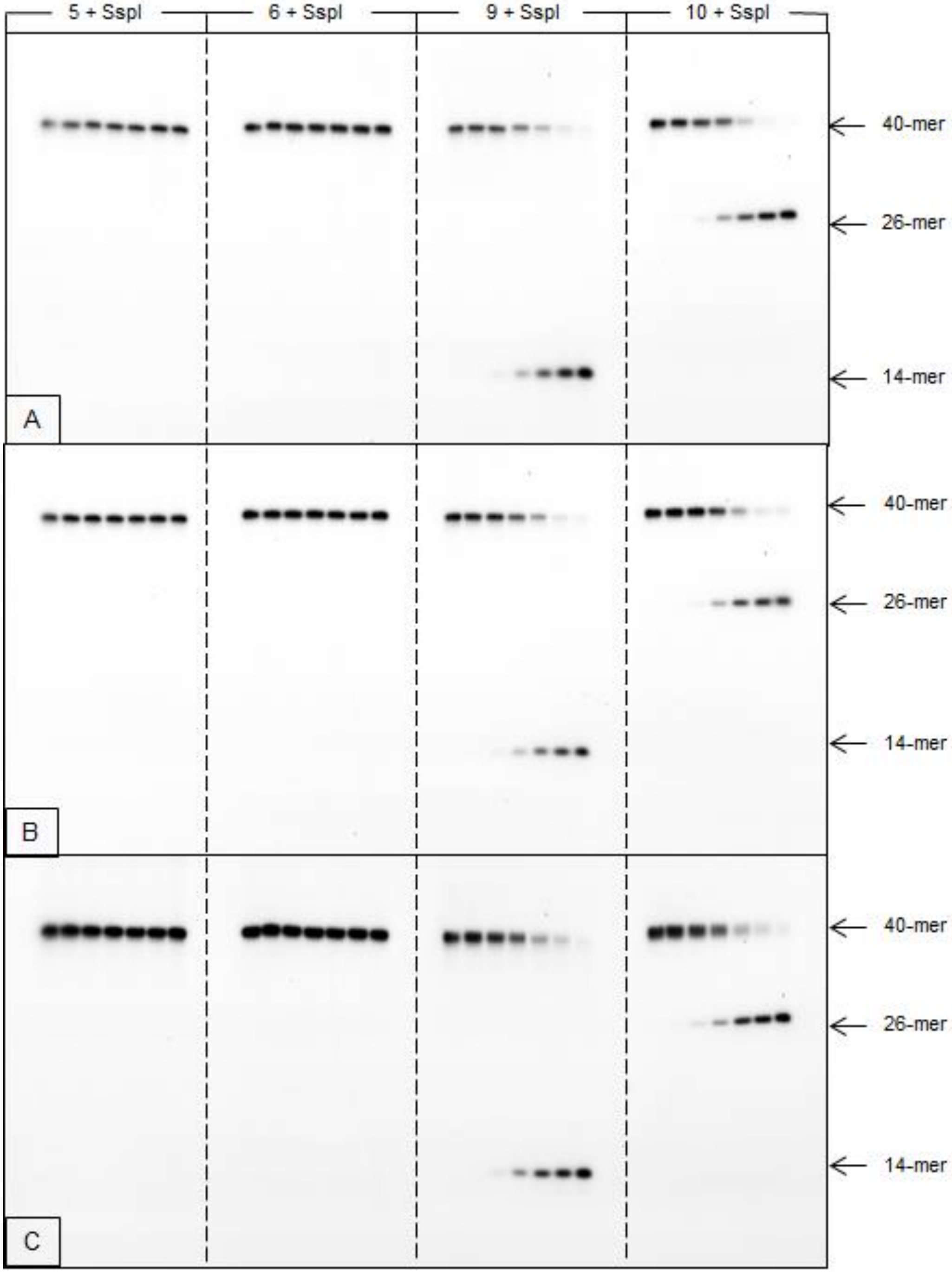

Figure S27. The autoradiograms of denaturing PAGE presenting cleavage of oligonucleotides by 1.5 U SspI – (A), (B) and (C) represent three repeats of assays. Lanes of bands represent following reaction times: 0, 1, 5, 15, 30, 45 and 60 min starting from the left.

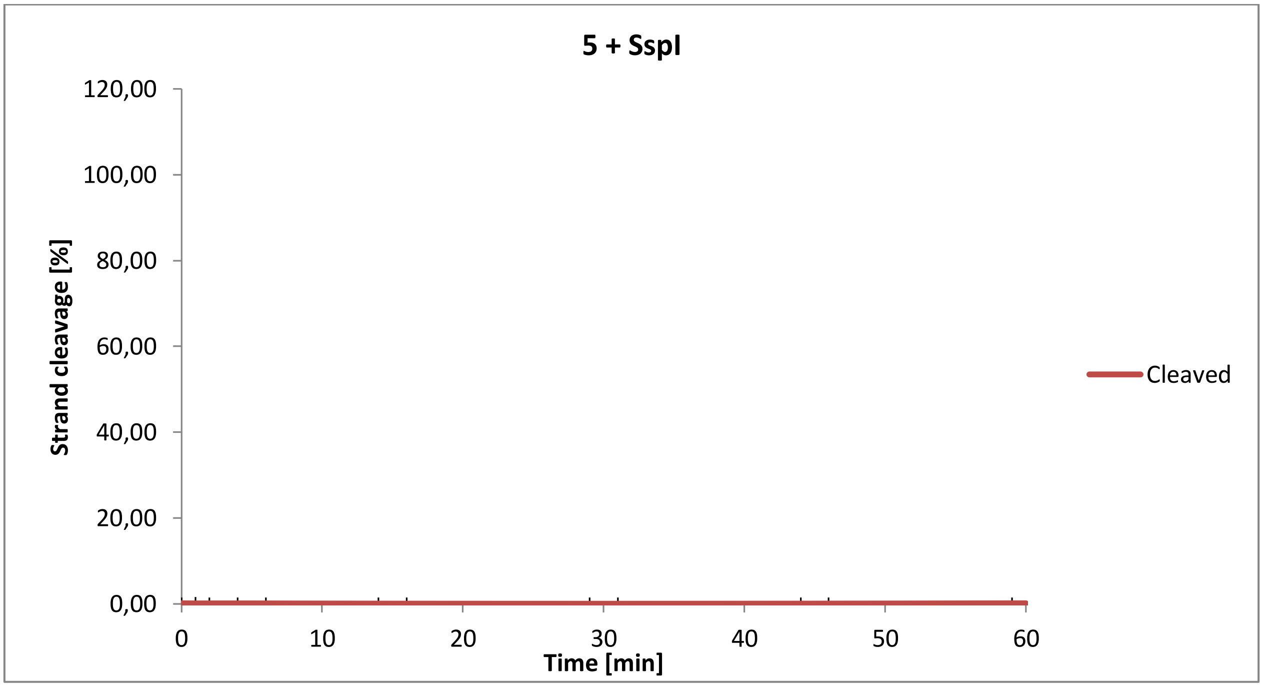

Figure S28. Cleavage of dsDNA (duplex C) by 1.5 U SspI. Quantity increase of cleaved ssDNA (strand 5) is shown.

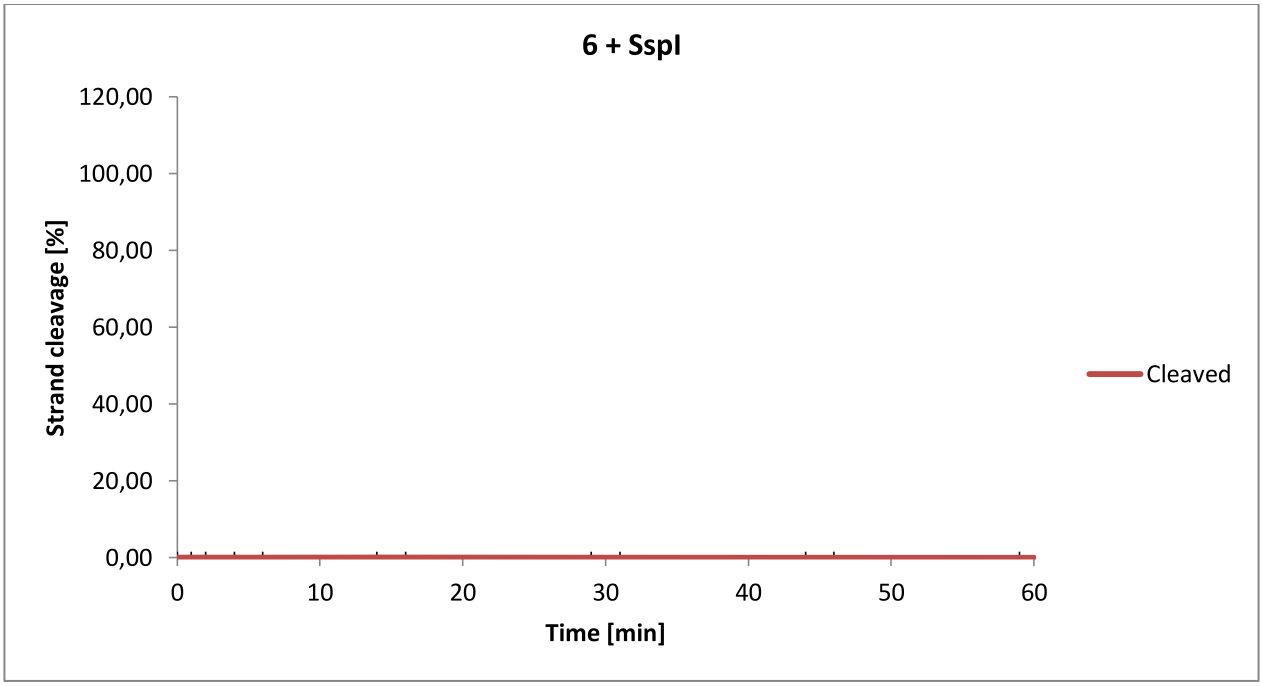

Figure S29. Cleavage of dsDNA (duplex C) by 1.5 U SspI. Quantity increase of cleaved ssDNA (strand 6) is shown.

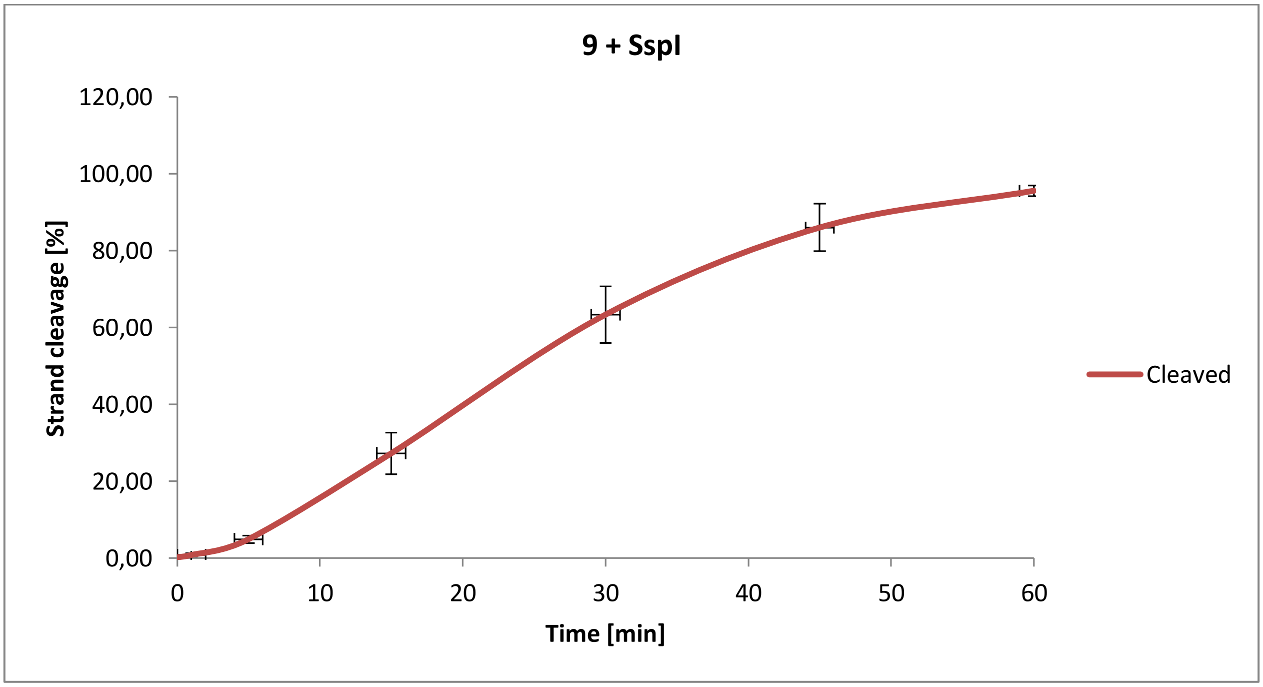

Figure S30. Cleavage of dsDNA (duplex E) by 1.5 U SspI. Quantity increase of cleaved ssDNA (strand 9) is shown.

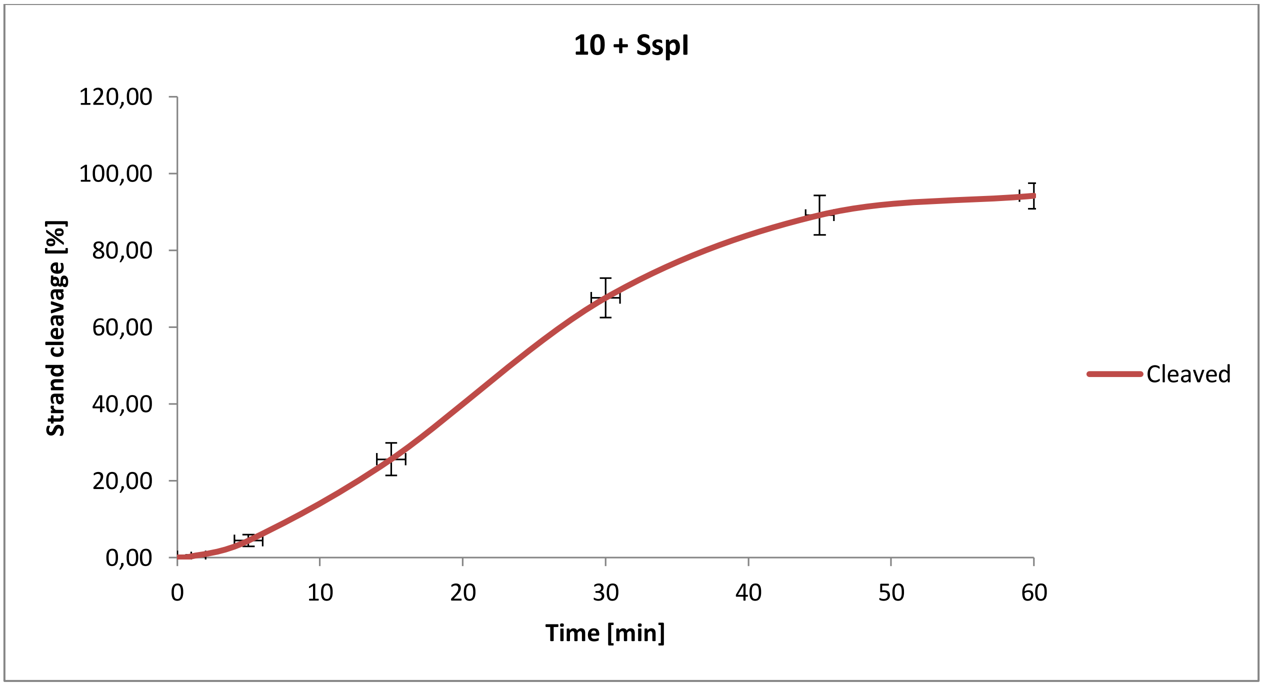

Figure S31. Cleavage of dsDNA (duplex E) by 1.5 U SspI. Quantity increase of cleaved ssDNA (strand 10) is shown.

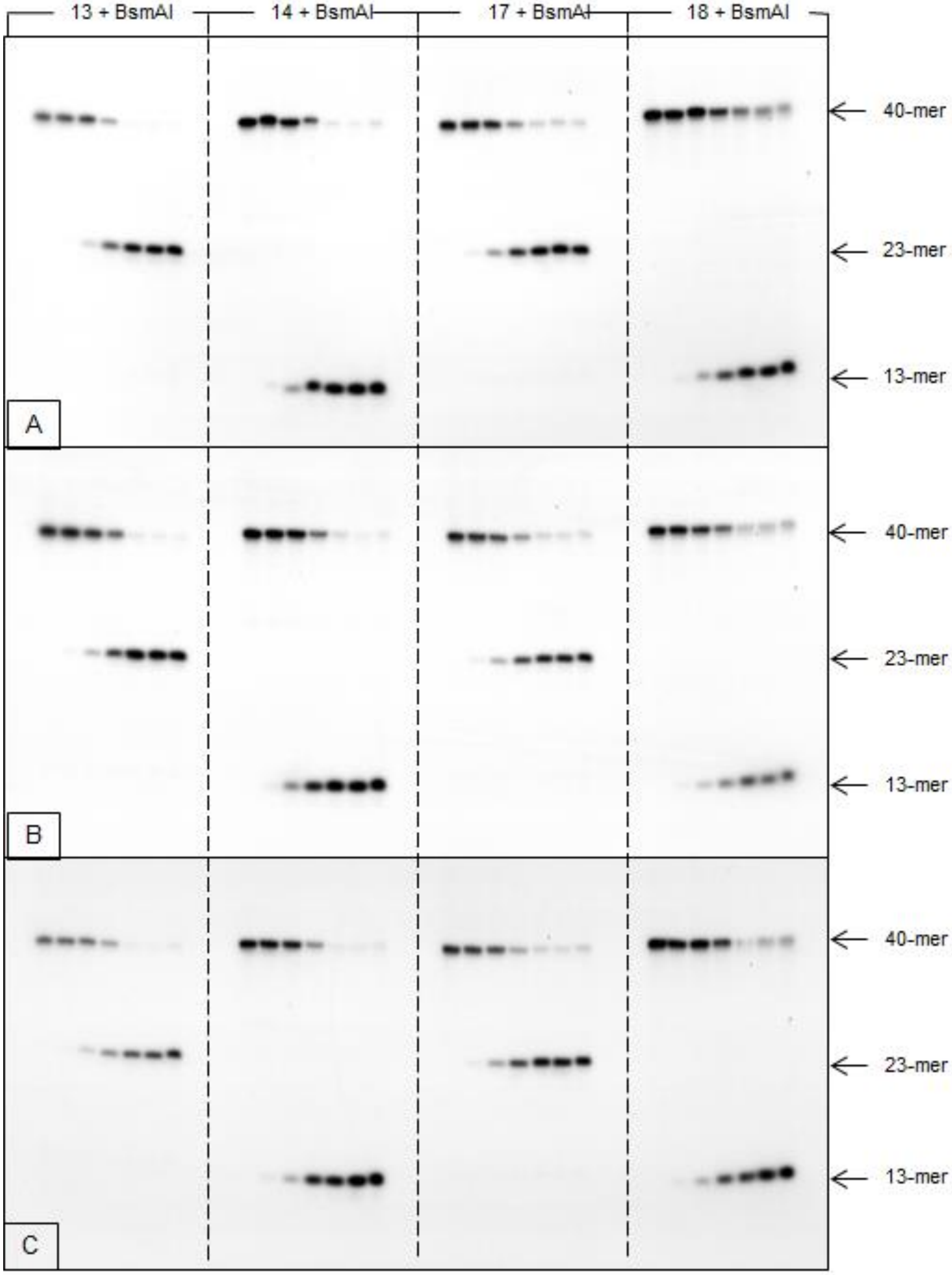

Figure S32. The autoradiograms of denaturing PAGE presenting cleavage of oligonucleotides by 0.5 U BsmAI – (A), (B) and (C) represent three repeats of assays. Lanes of bands represent following reaction times: 0, 1, 5, 15, 30, 45 and 60 min starting from the left.

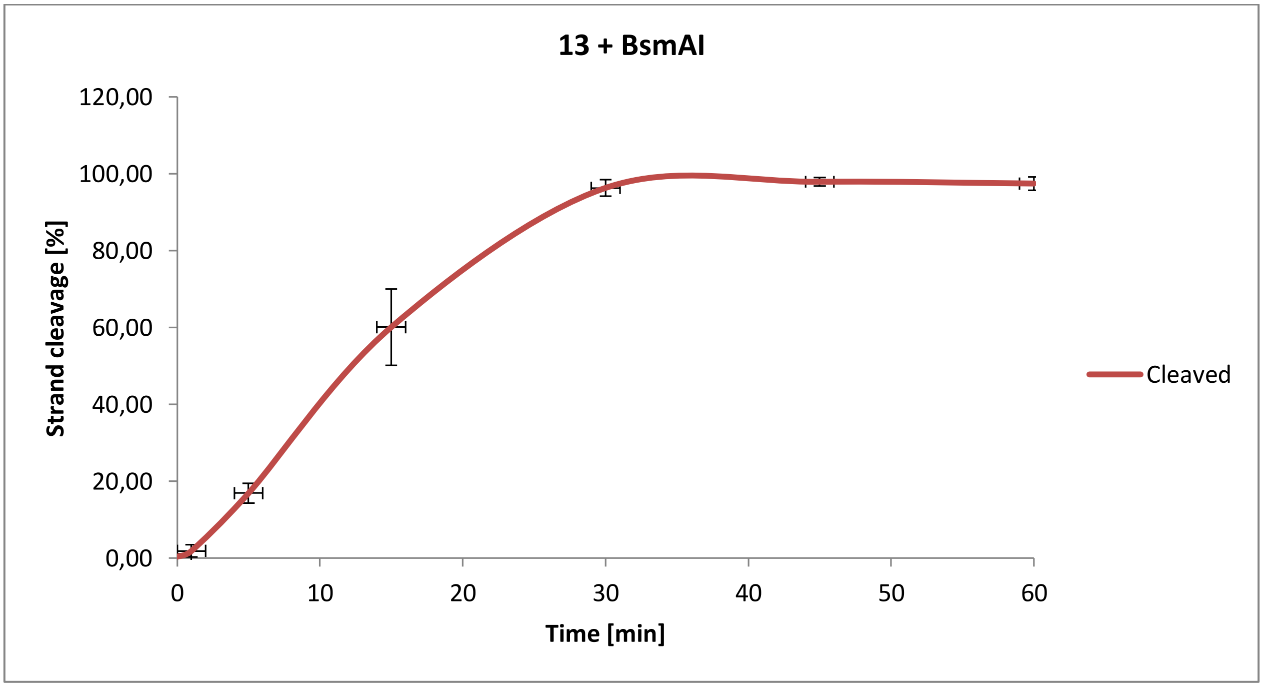

Figure S33. Cleavage of dsDNA (duplex G) by 0.5 U BsmAI. Quantity increase of cleaved ssDNA (strand 13) is shown.

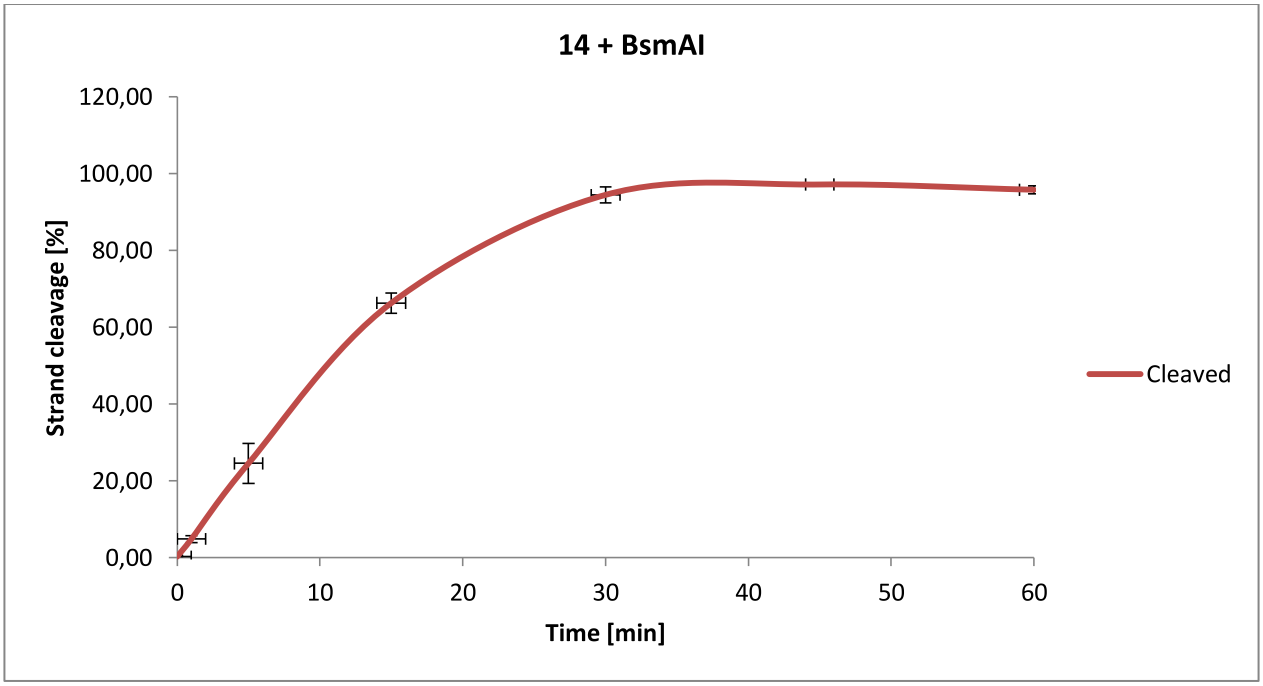

Figure S34. Cleavage of dsDNA (duplex G) by 0.5 U BsmAI. Quantity increase of cleaved ssDNA (strand 14) is shown.

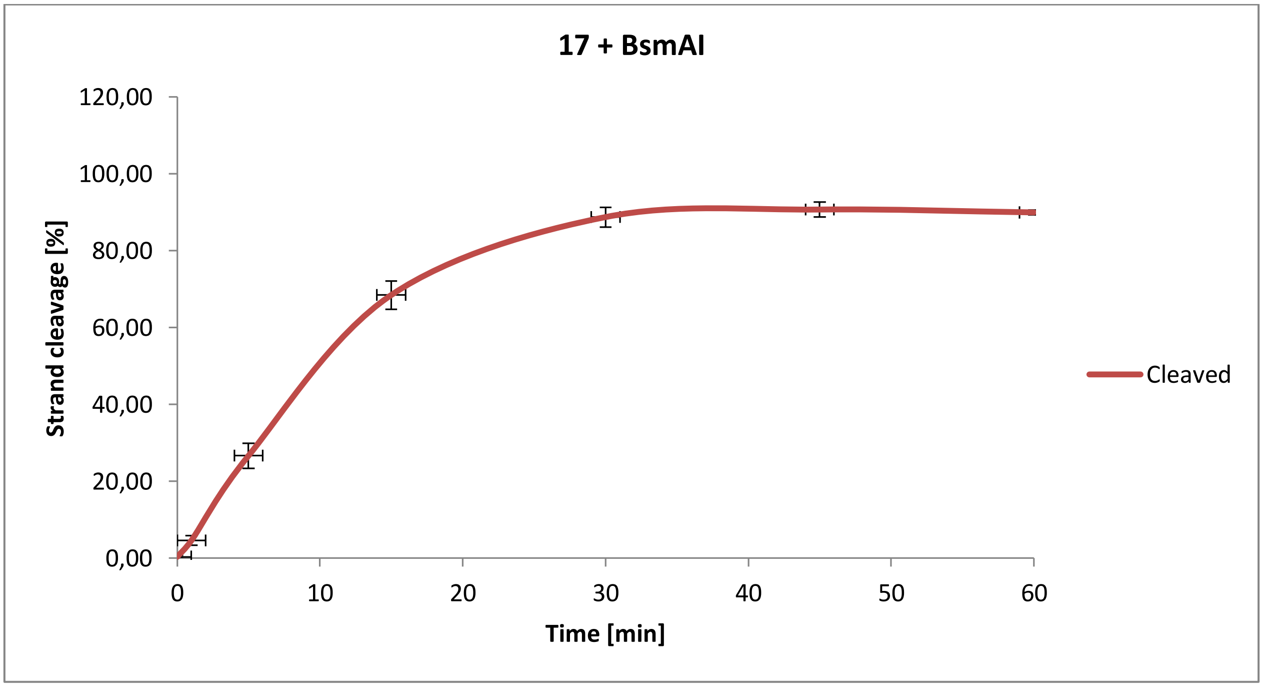

Figure S35. Cleavage of dsDNA (duplex I) by 0.5 U BsmAI. Quantity increase of cleaved ssDNA (strand 17) is shown.

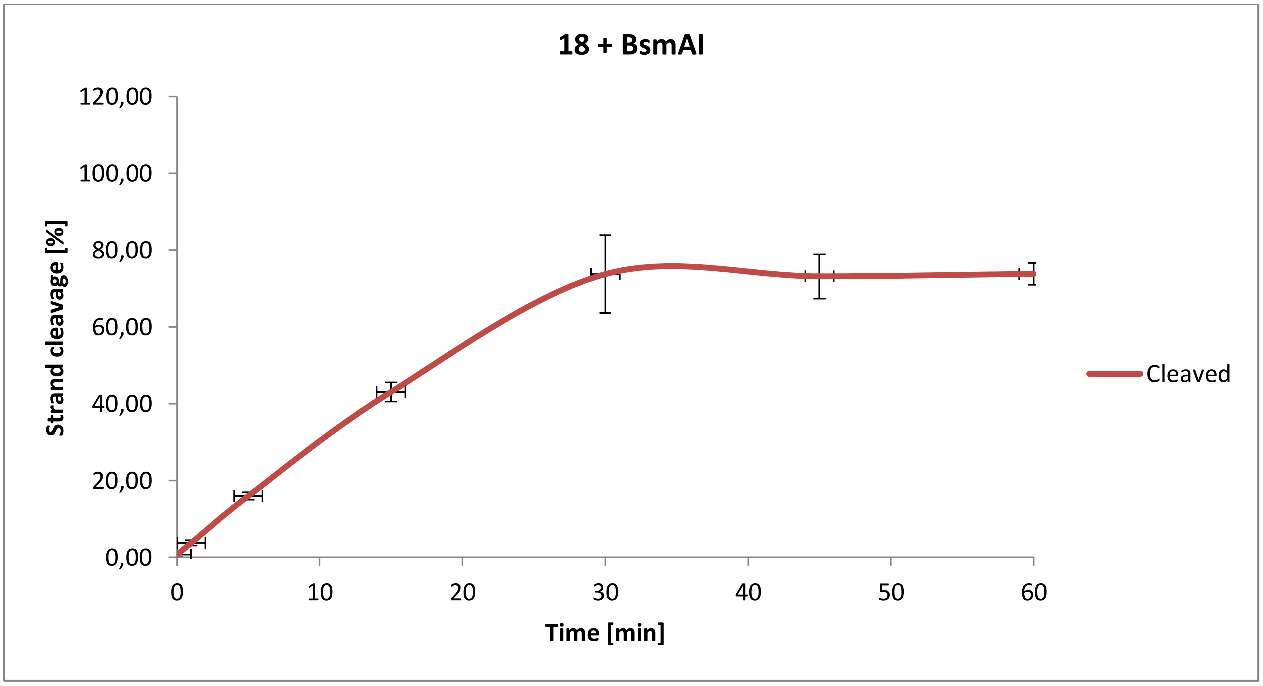

Figure S36. Cleavage of dsDNA (duplex I) by 0.5 U BsmAI. Quantity increase of cleaved ssDNA (strand 18) is shown.

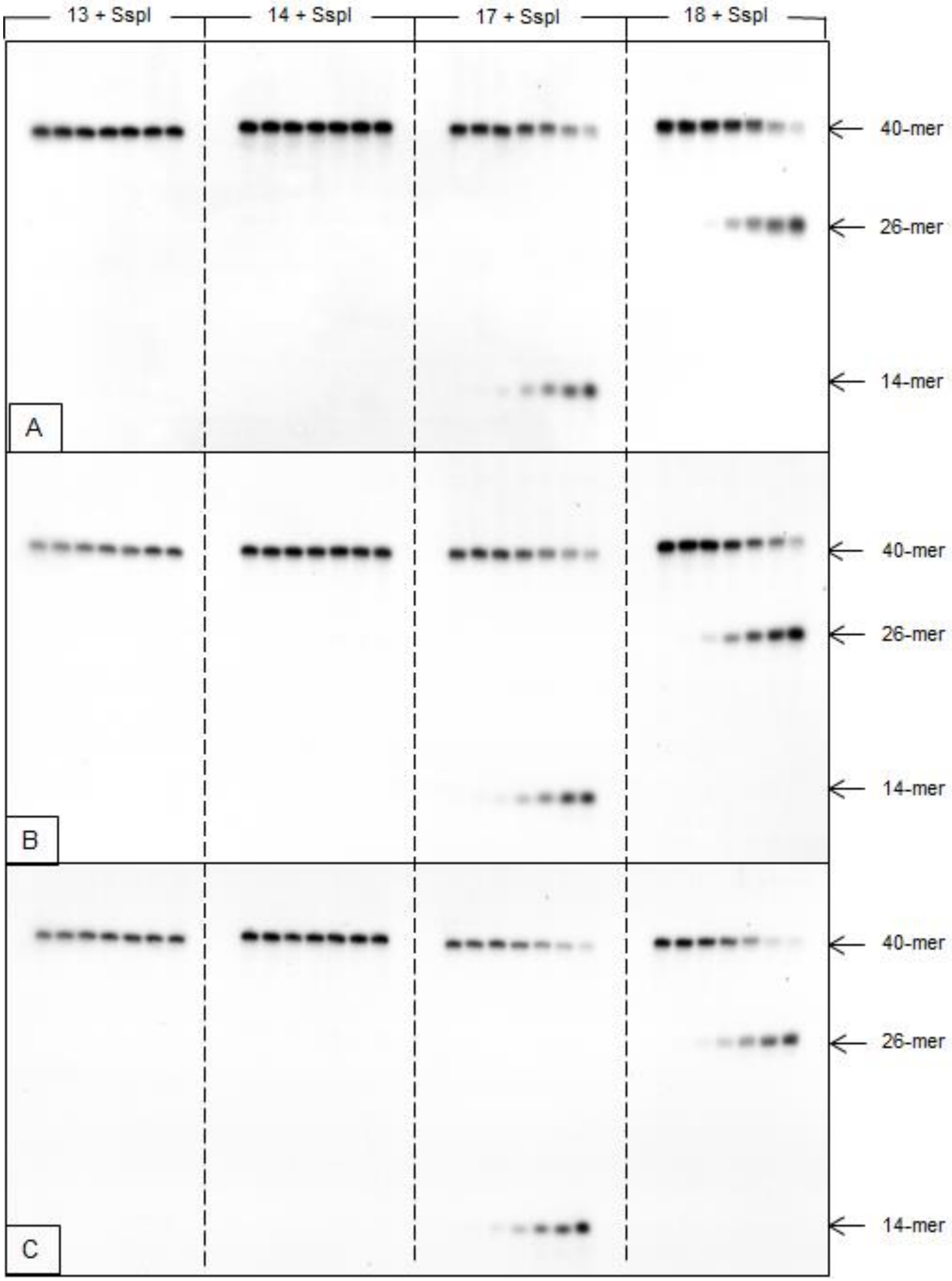

Figure S37. The autoradiograms of denaturing PAGE presenting cleavage of oligonucleotides by 1.5 U SspI – (A), (B) and (C) represent three repeats of assays. Lanes of bands represent following reaction times: 0, 1, 5, 15, 30, 45 and 60 min starting from the left.

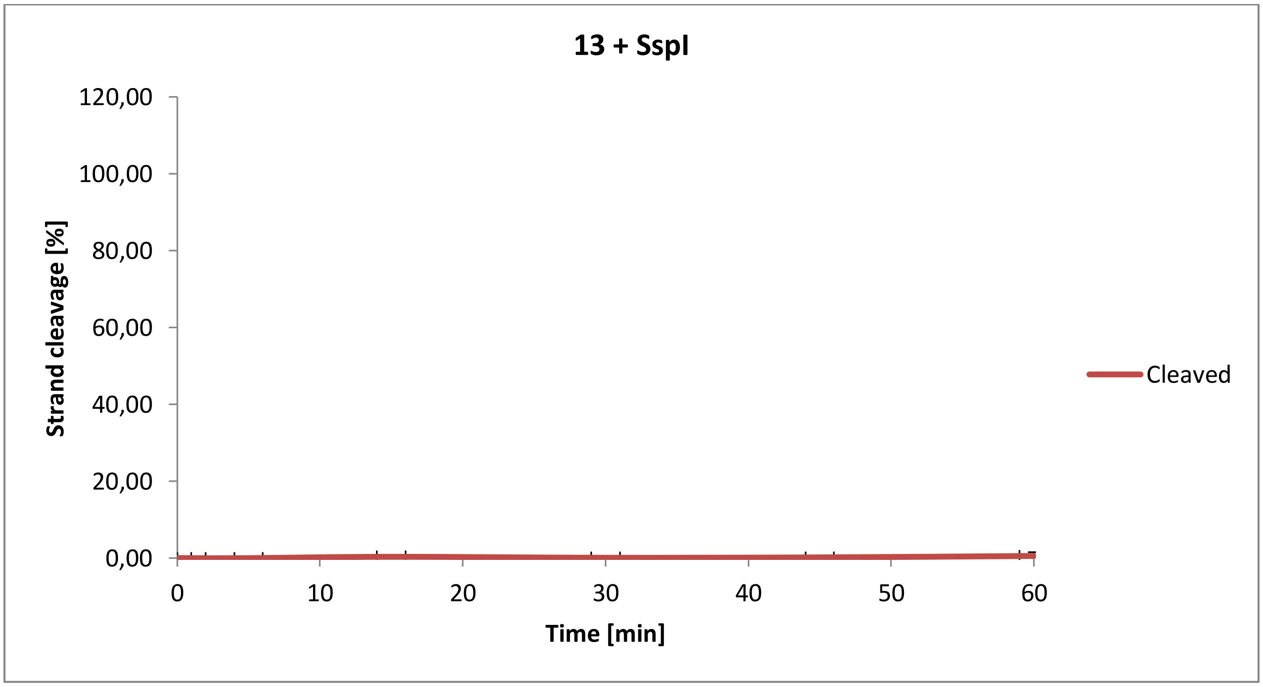

Figure S38. Cleavage of dsDNA (duplex G) by 1.5 U SspI. Quantity increase of cleaved ssDNA (strand 13) is shown.

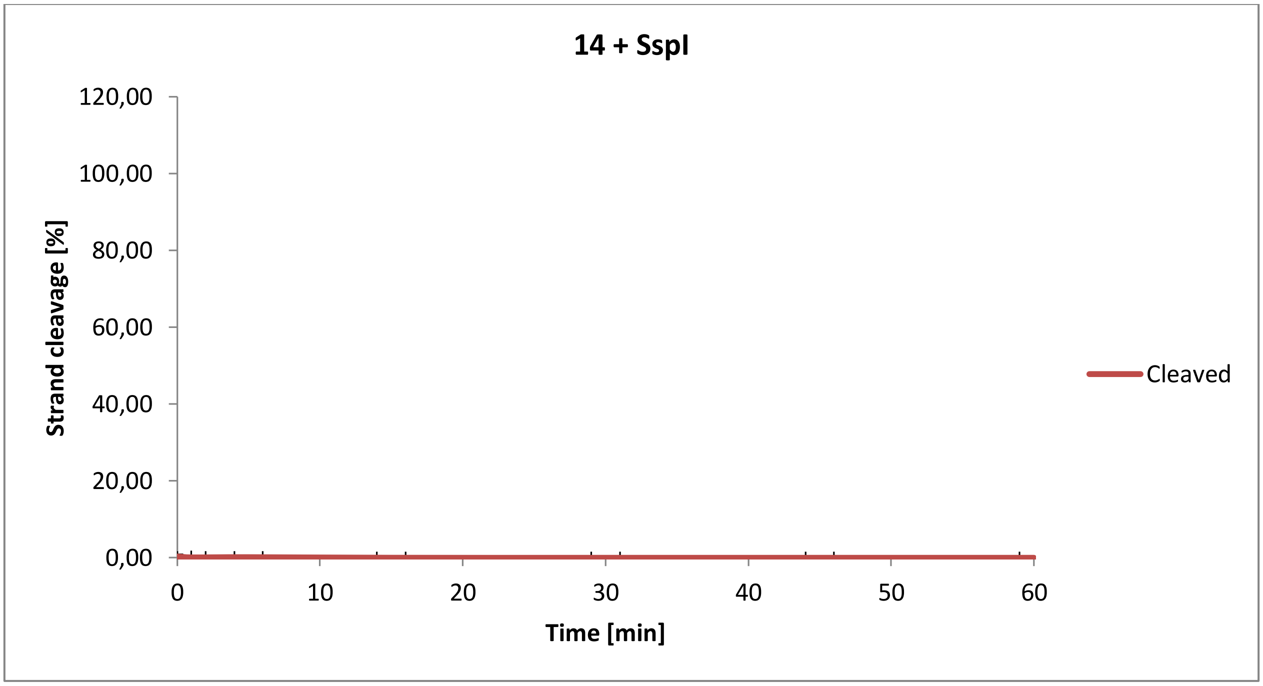

Figure S39. Cleavage of dsDNA (duplex G) by 1.5 U SspI. Quantity increase of cleaved ssDNA (strand 14) is shown.

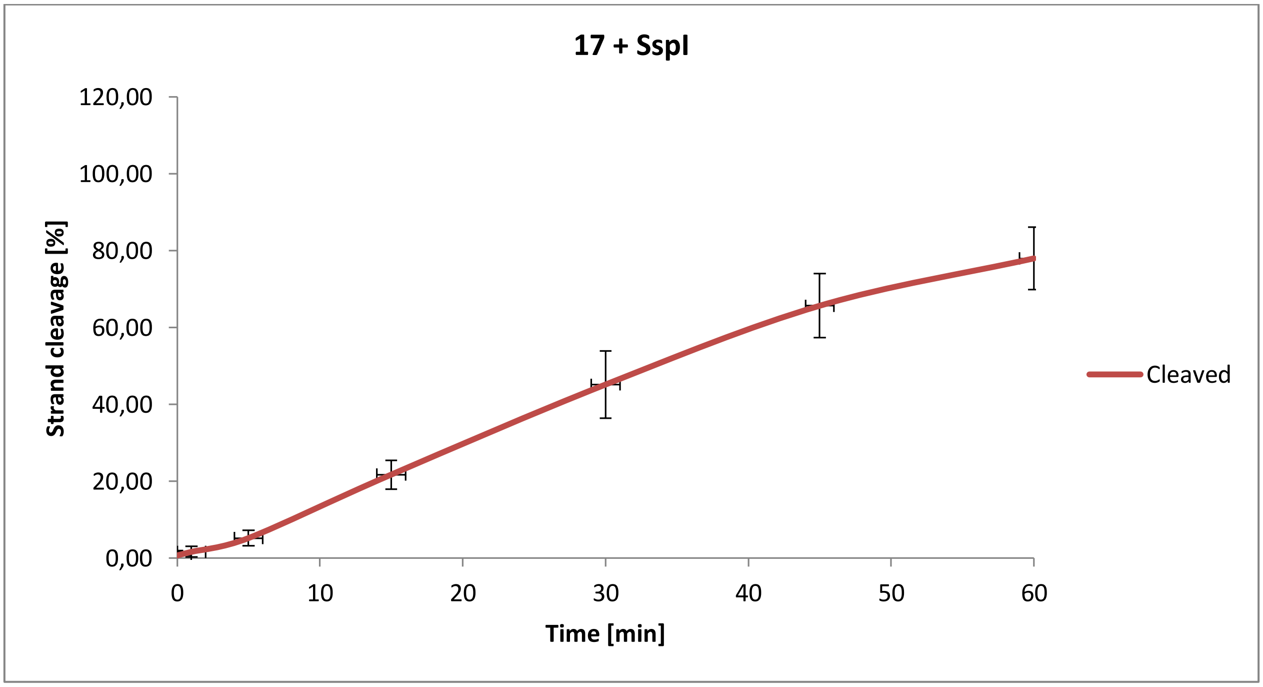

Figure S40. Cleavage of dsDNA (duplex I) by 1.5 U SspI. Quantity increase of cleaved ssDNA (strand 17) is shown.

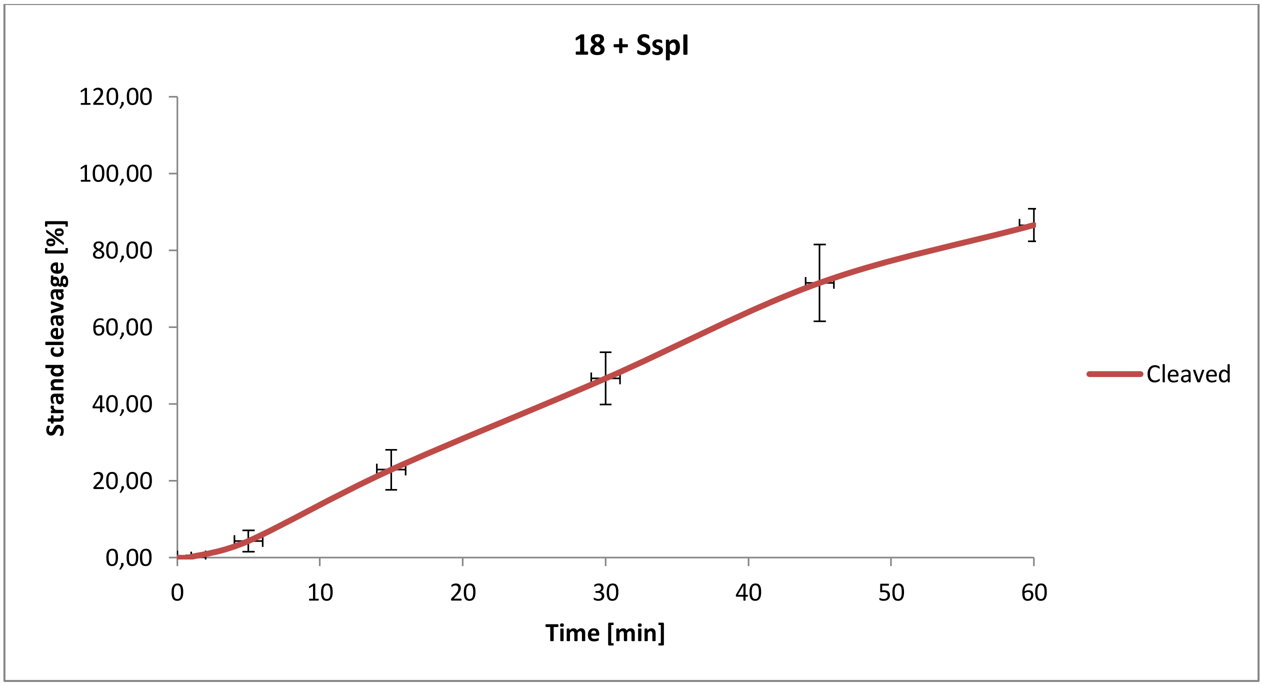

Figure S41. Cleavage of dsDNA (duplex I) by 1.5 U SspI. Quantity increase of cleaved ssDNA (strand 18) is shown.

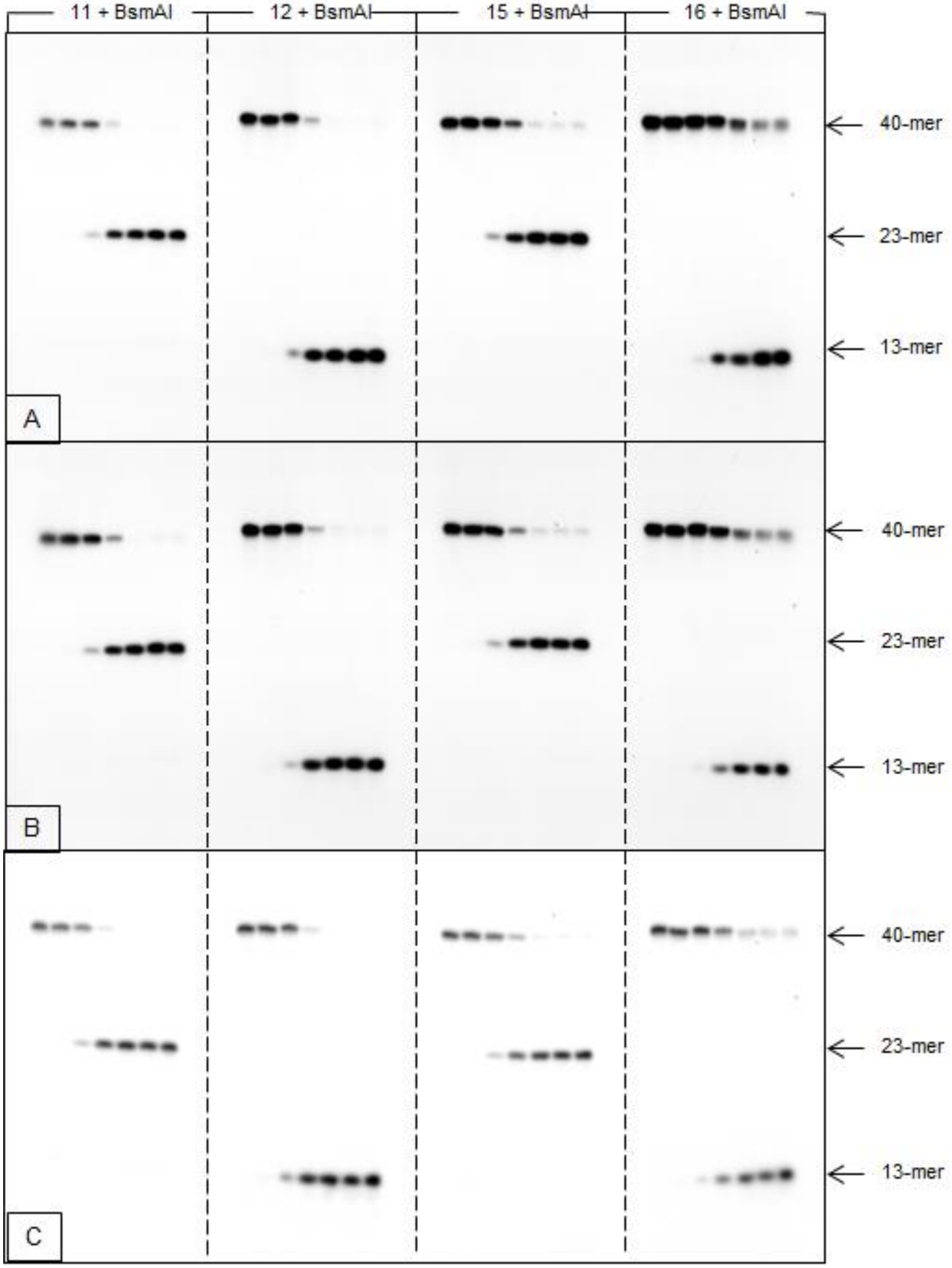

Figure S42. The autoradiograms of denaturing PAGE presenting cleavage of oligonucleotides by 0.5 U BsmAI – (A), (B) and (C) represent three repeats of assays. Lanes of bands represent following reaction times: 0, 1, 5, 15, 30, 45 and 60 min starting from the left.

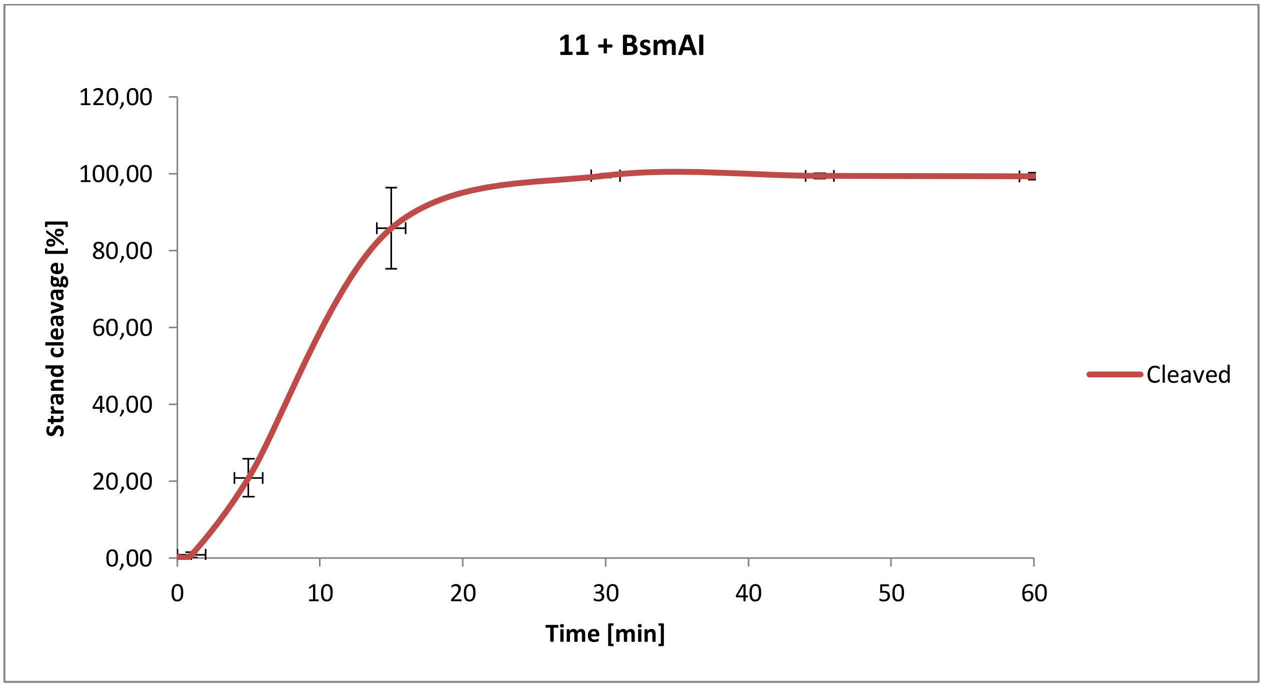

Figure S43. Cleavage of dsDNA (duplex F) by 0.5 U BsmAI. Quantity increase of cleaved ssDNA (strand 11) is shown.

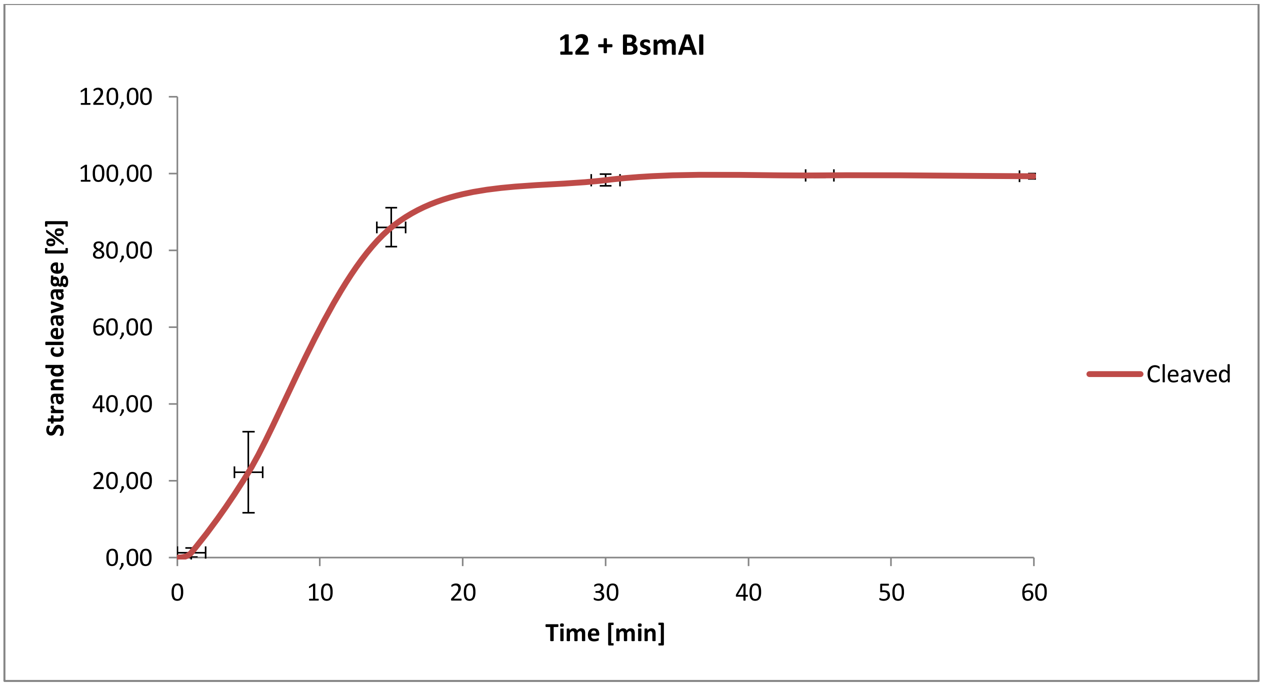

Figure S44. Cleavage of dsDNA (duplex F) by 0.5 U BsmAI. Quantity increase of cleaved ssDNA (strand 12) is shown.

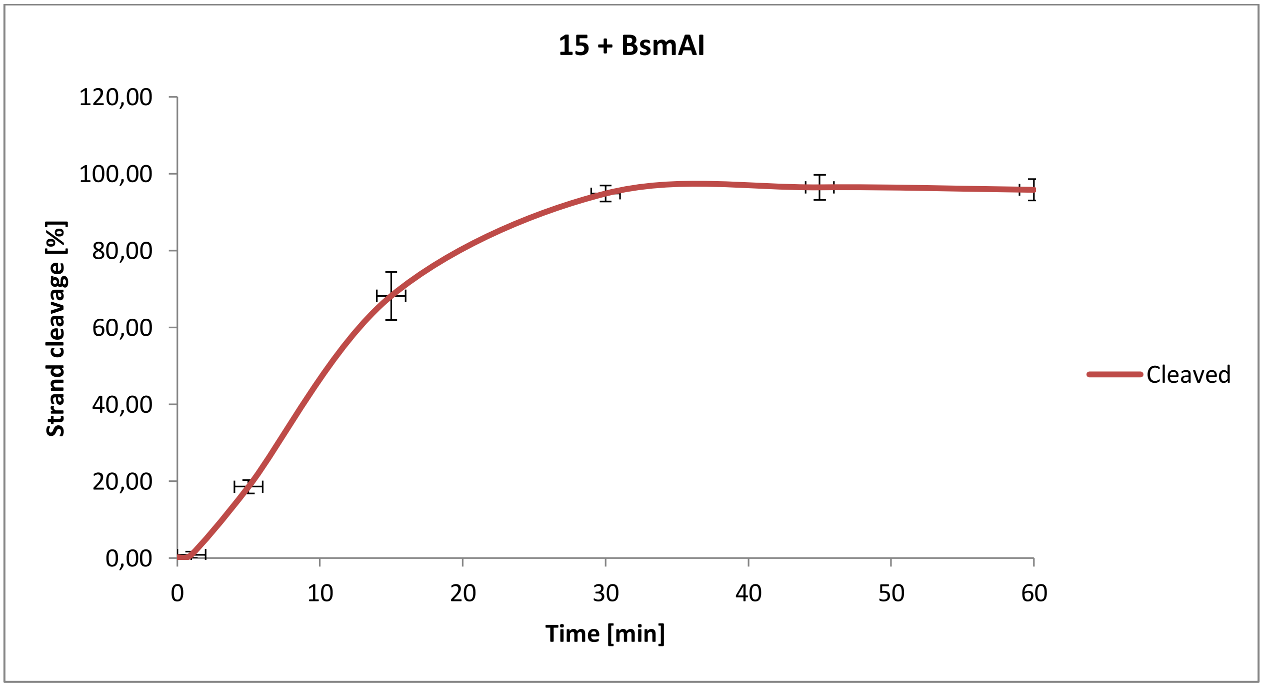

Figure S45. Cleavage of dsDNA (duplex H) by 0.5 U BsmAI. Quantity increase of cleaved ssDNA (strand 15) is shown.

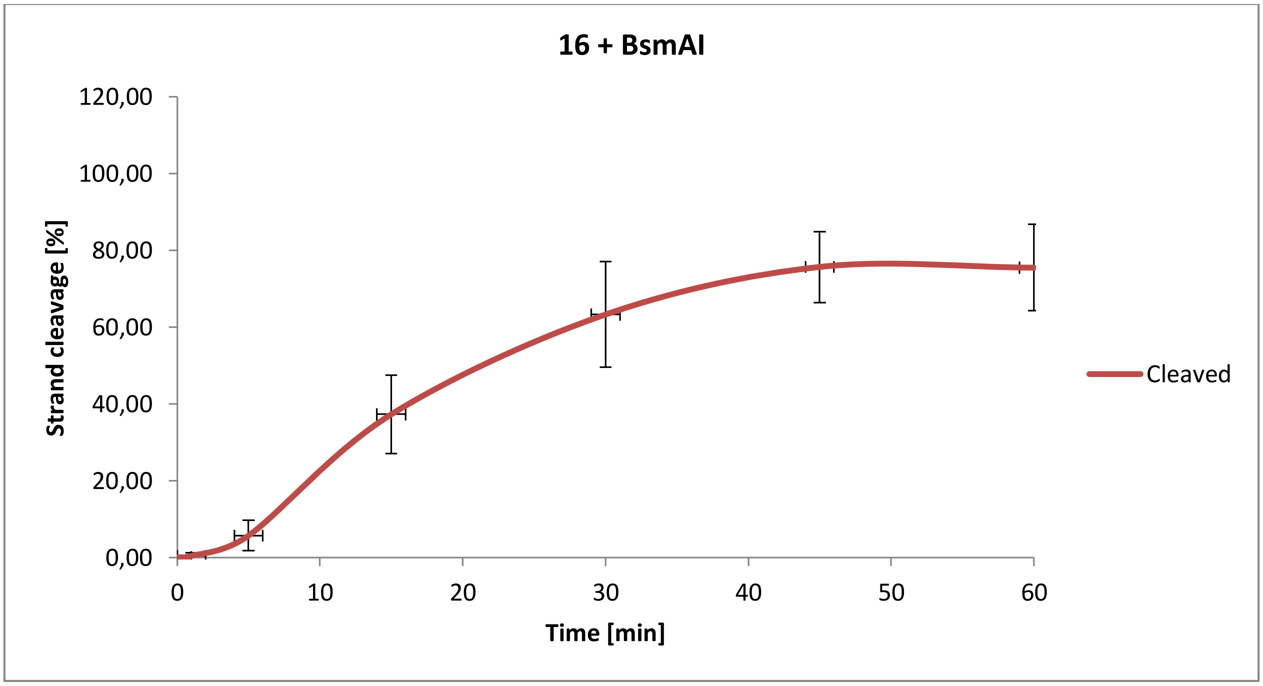

Figure S46. Cleavage of dsDNA (duplex H) by 0.5 U BsmAI. Quantity increase of cleaved ssDNA (strand 16) is shown.

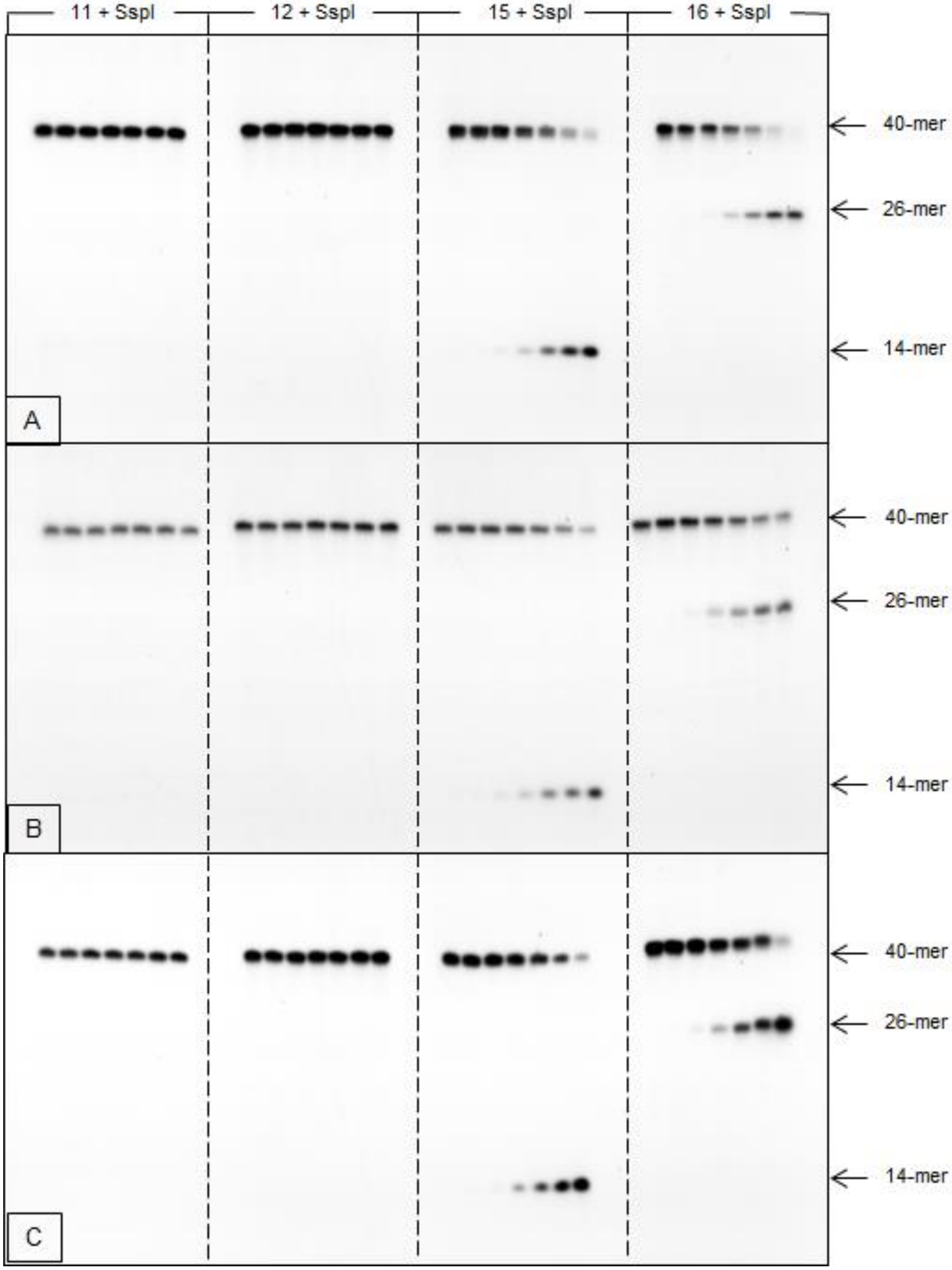

Figure S47. The autoradiograms of denaturing PAGE presenting cleavage of oligonucleotides by 1.5 U SspI – (A), (B) and (C) represent three repeats of assays. Lanes of bands represent following reaction times: 0, 1, 5, 15, 30, 45 and 60 min starting from the left.

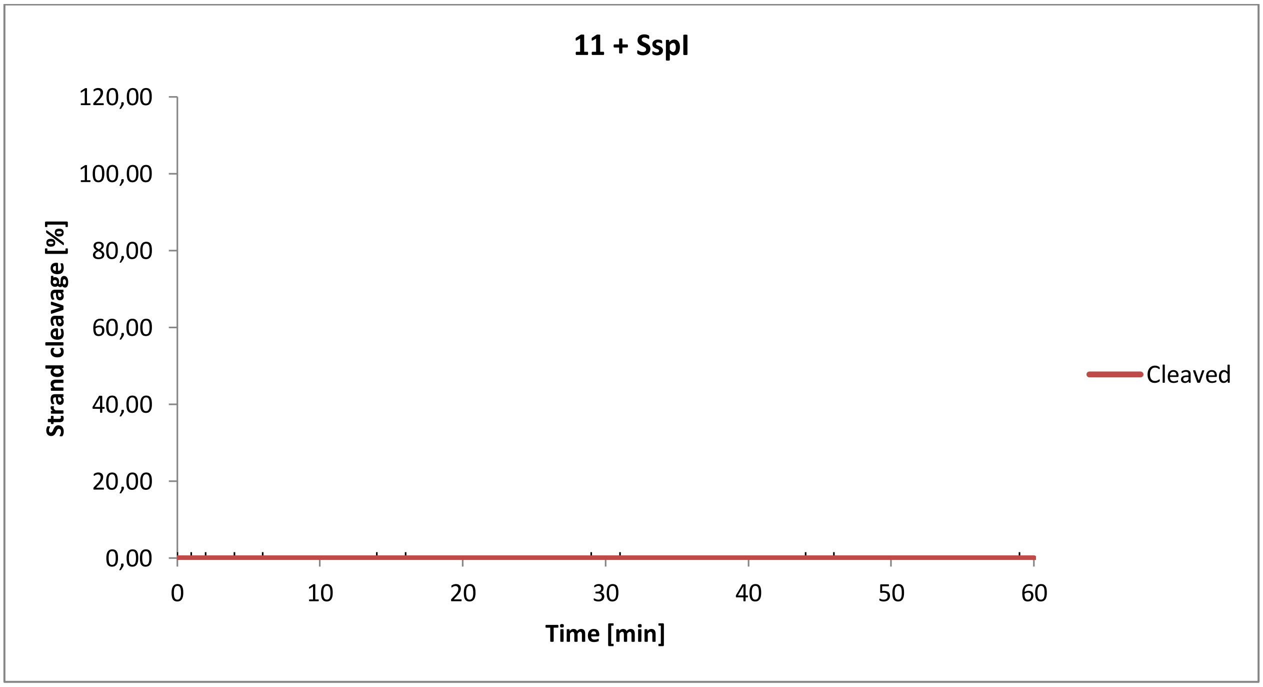

Figure S48. Cleavage of dsDNA (duplex F) by 1.5 U SspI. Quantity increase of cleaved ssDNA (strand 11) is shown.

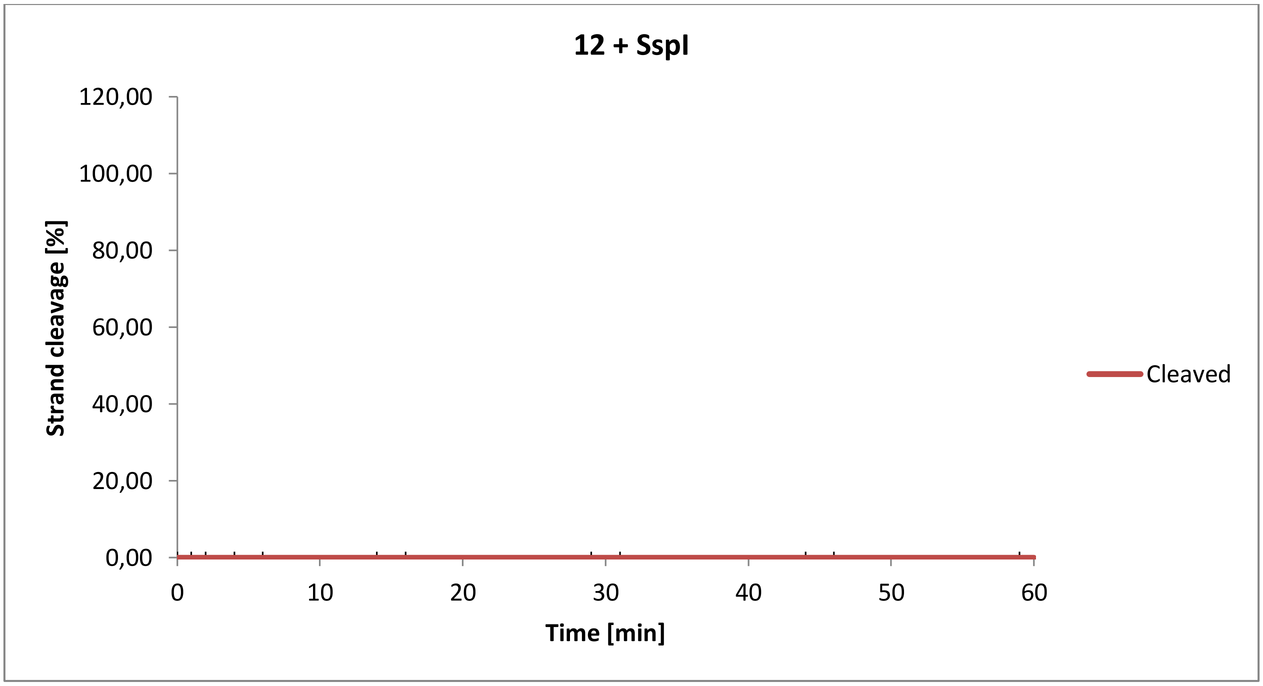

Figure S49. Cleavage of dsDNA (duplex F) by 1.5 U SspI. Quantity increase of cleaved ssDNA (strand 12) is shown.

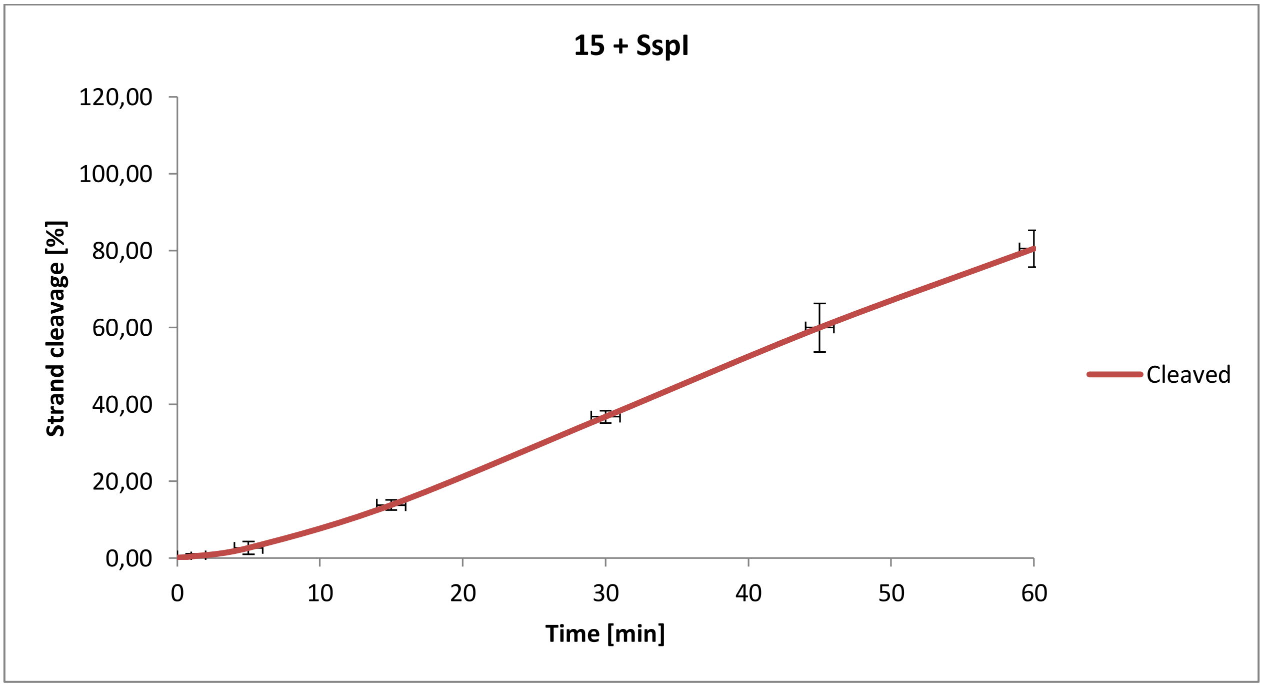

Figure S50. Cleavage of dsDNA (duplex H) by 1.5 U SspI. Quantity increase of cleaved ssDNA (strand 15) is shown.

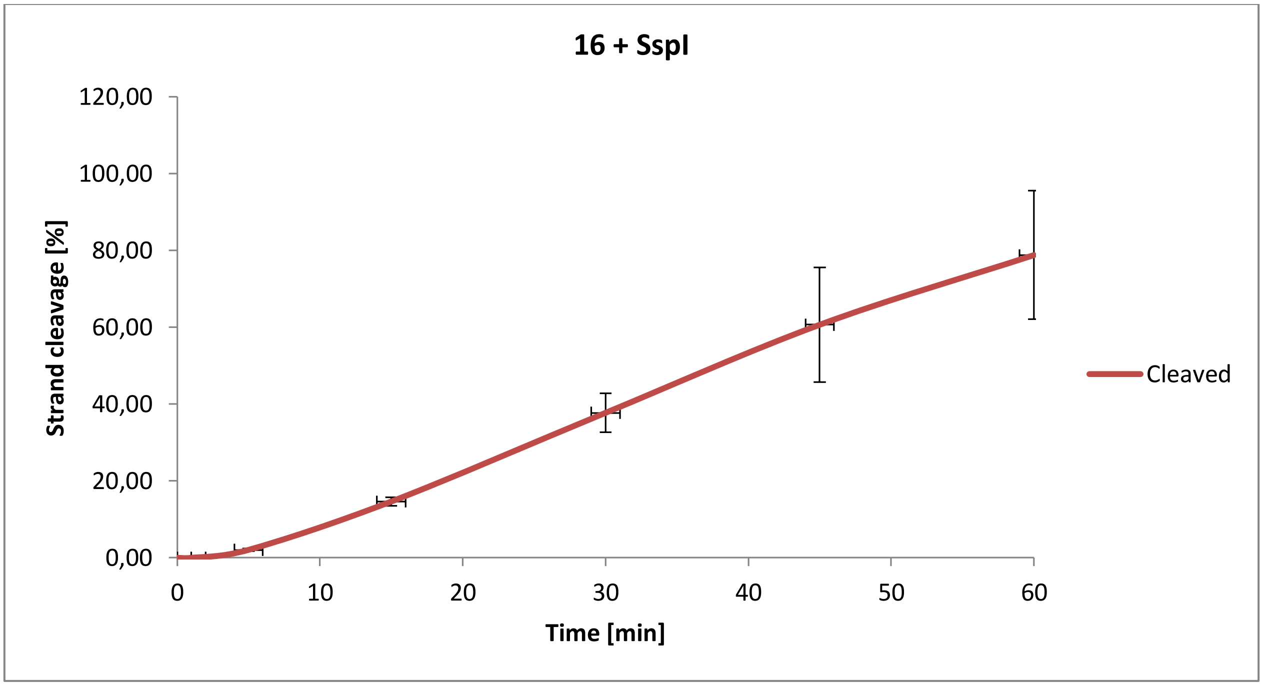

Figure S51. Cleavage of dsDNA (duplex H) by 1.5 U SspI. Quantity increase of cleaved ssDNA (strand 16) is shown.

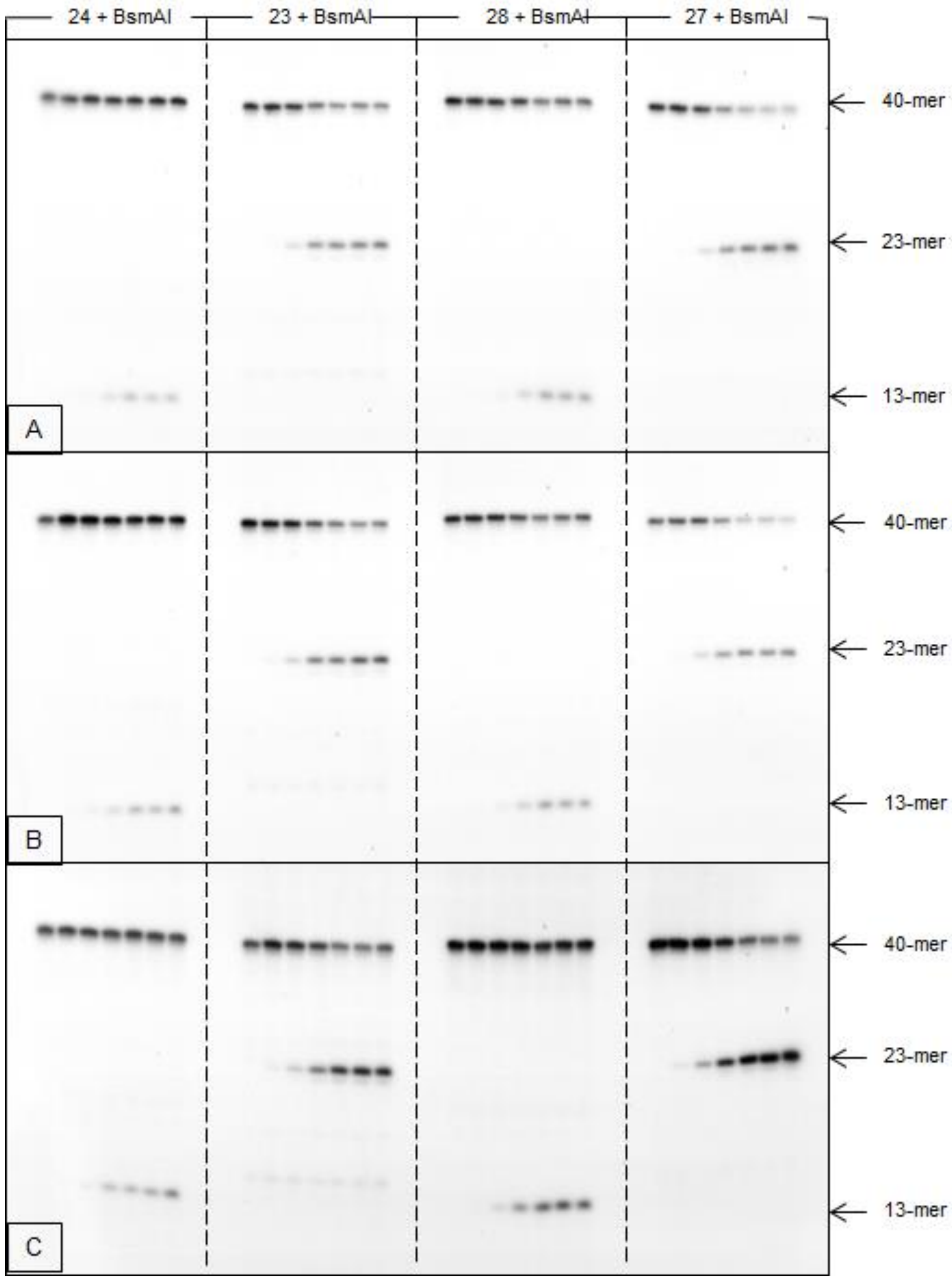

Figure S52. The autoradiograms of denaturing PAGE presenting cleavage of oligonucleotides by 0.5 U BsmAI – (A), (B) and (C) represent three repeats of assays. Lanes of bands represent following reaction times: 0, 1, 5, 15, 30, 45 and 60 min starting from the left.

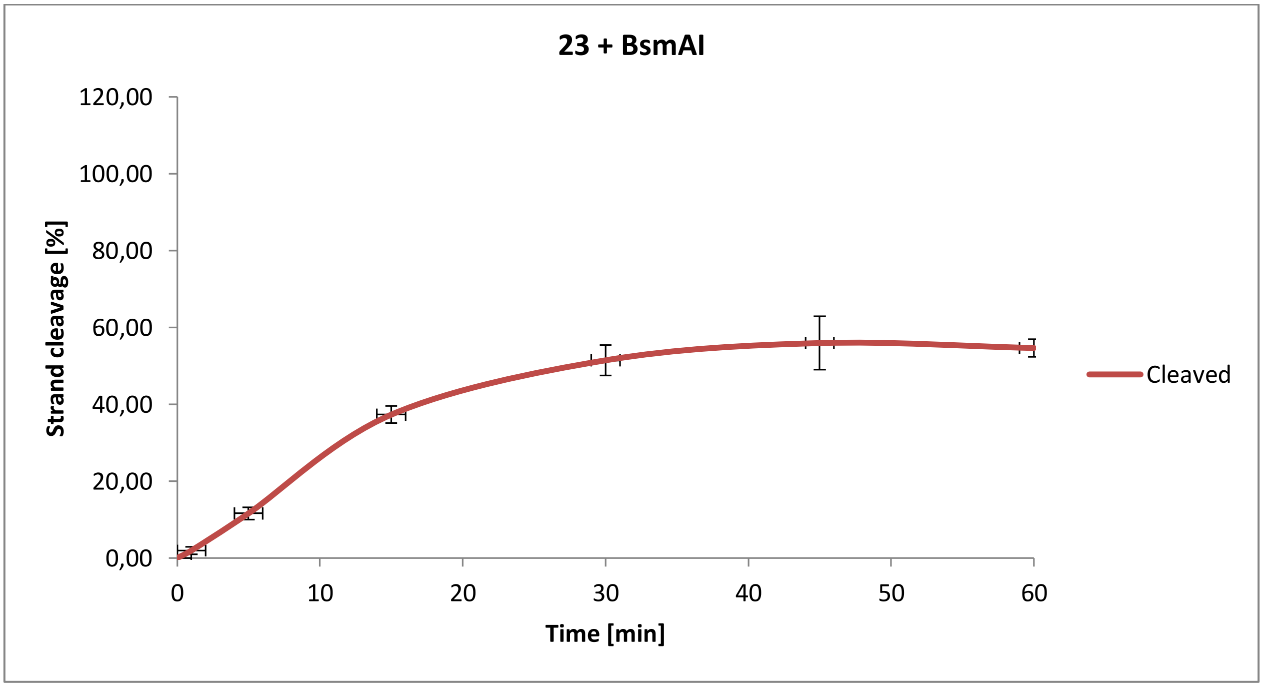

Figure S53. Cleavage of dsDNA (duplex L) by 0.5 U BsmAI. Quantity increase of cleaved ssDNA (strand 23) is shown.

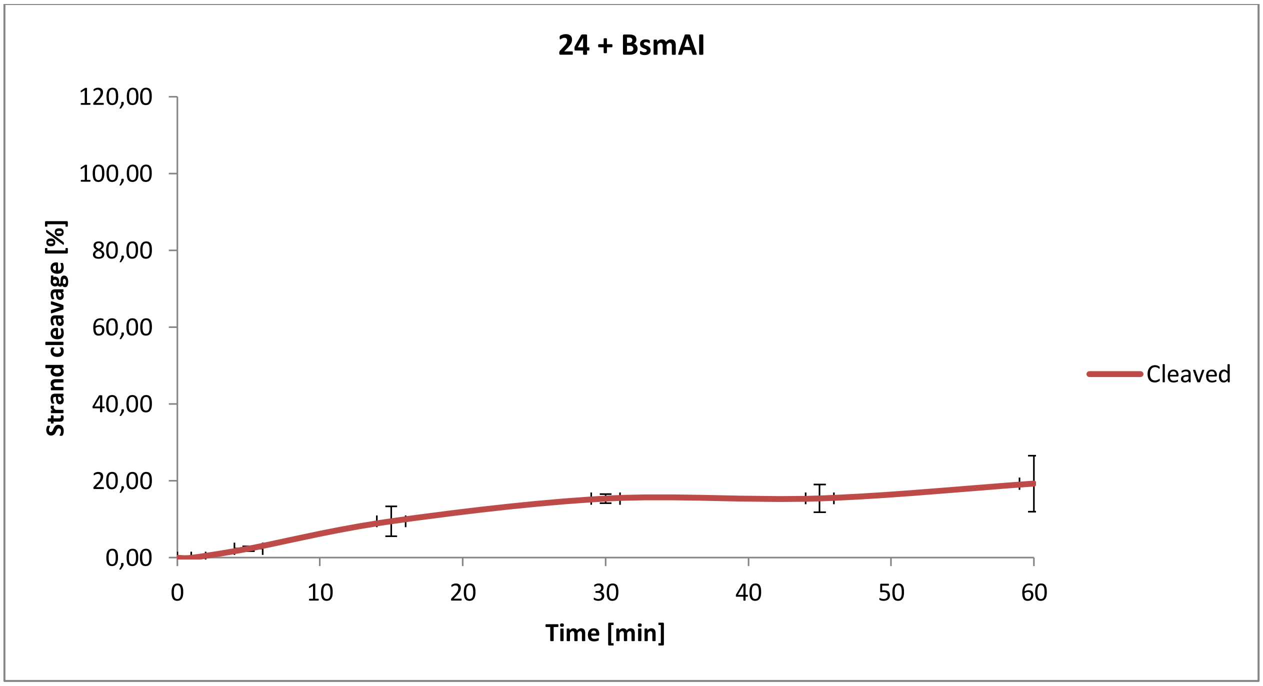

Figure S54. Cleavage of dsDNA (duplex L) by 0.5 U BsmAI. Quantity increase of cleaved ssDNA (strand 24) is shown.

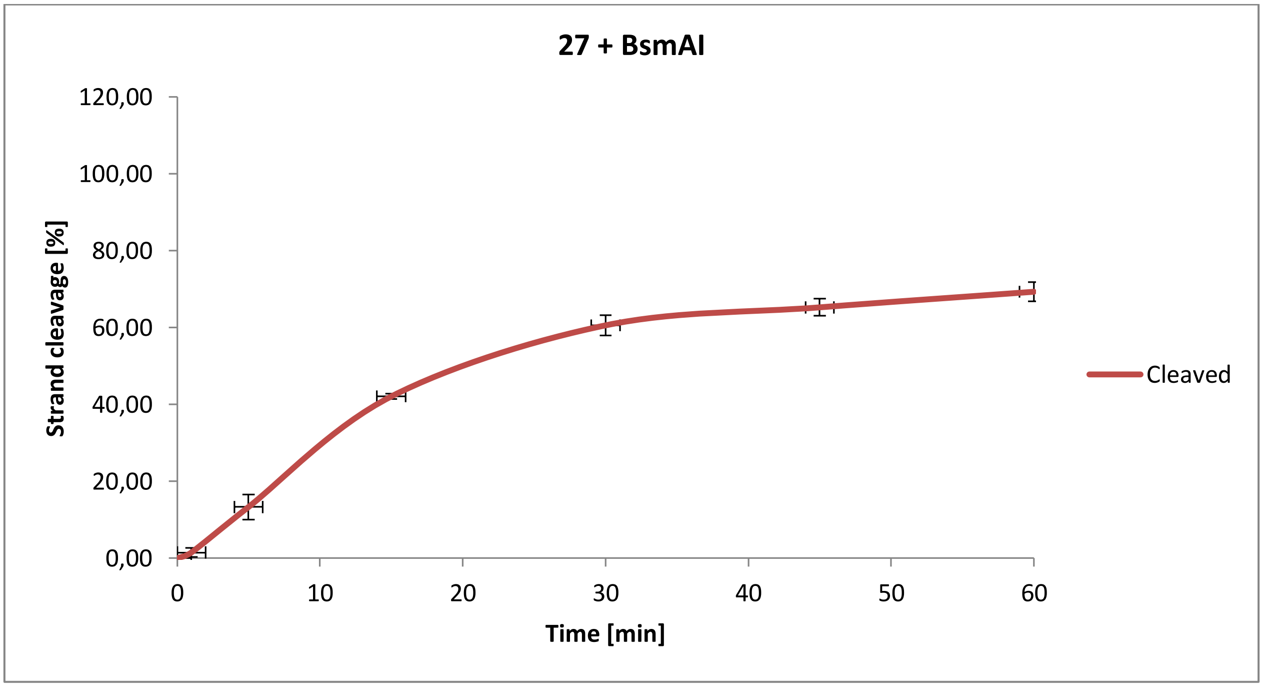

Figure S55. Cleavage of dsDNA (duplex N) by 0.5 U BsmAI. Quantity increase of cleaved ssDNA (strand 27) is shown.

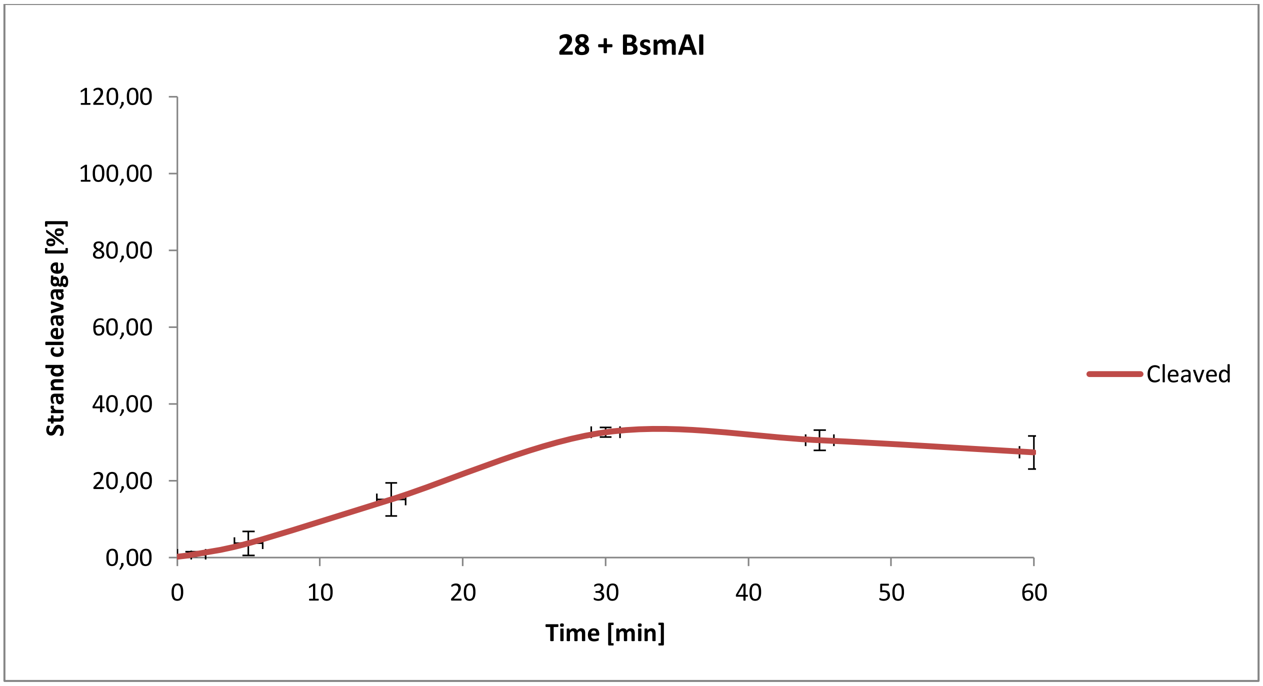

Figure S56. Cleavage of dsDNA (duplex N) by 0.5 U BsmAI. Quantity increase of cleaved ssDNA (strand 28) is shown.

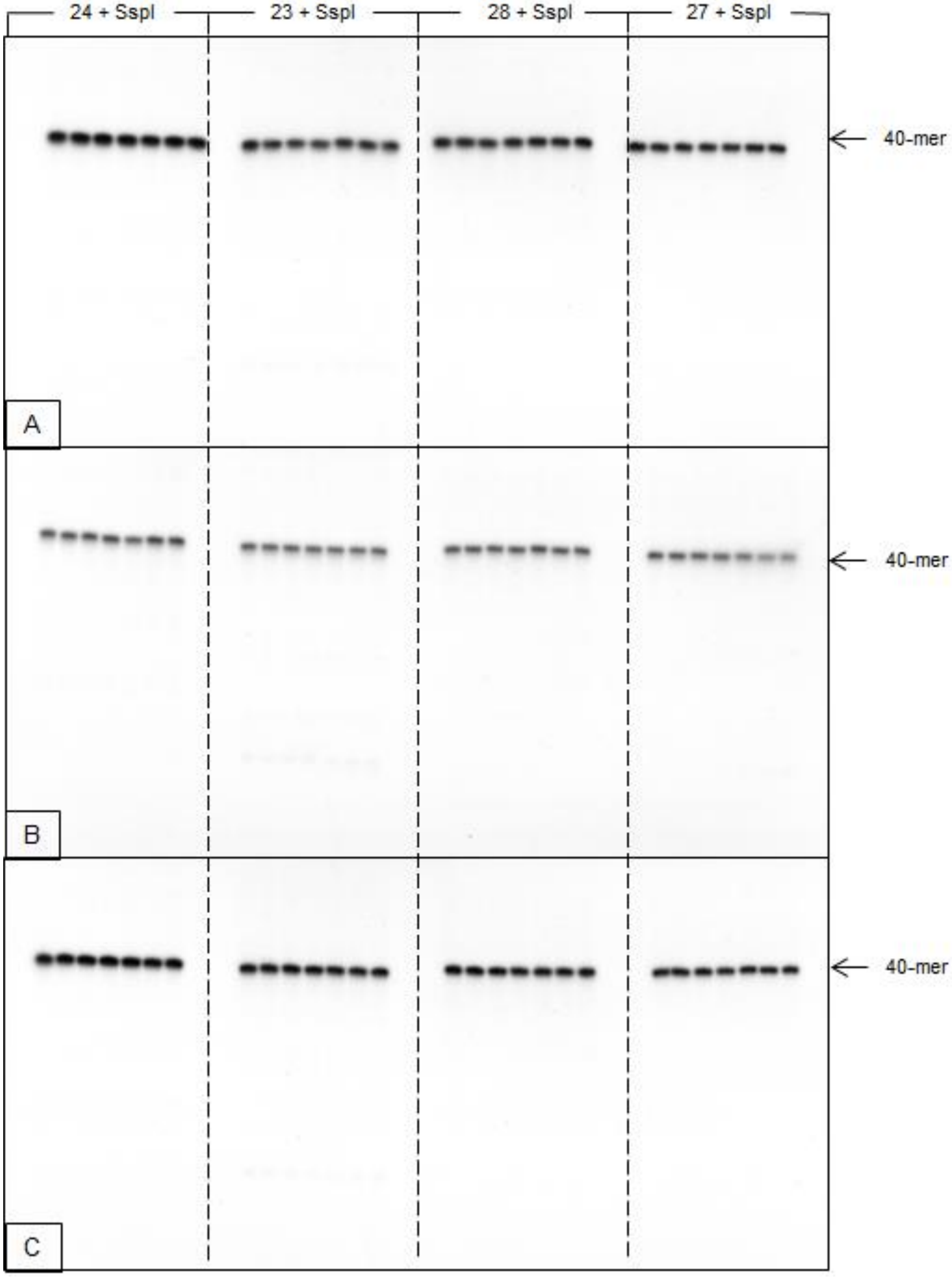

Figure S57. The autoradiograms of denaturing PAGE presenting cleavage of oligonucleotides by 1.5 U SspI – (A), (B) and (C) represent three repeats of assays. Lanes of bands represent following reaction times: 0, 1, 5, 15, 30, 45 and 60 min starting from the left.

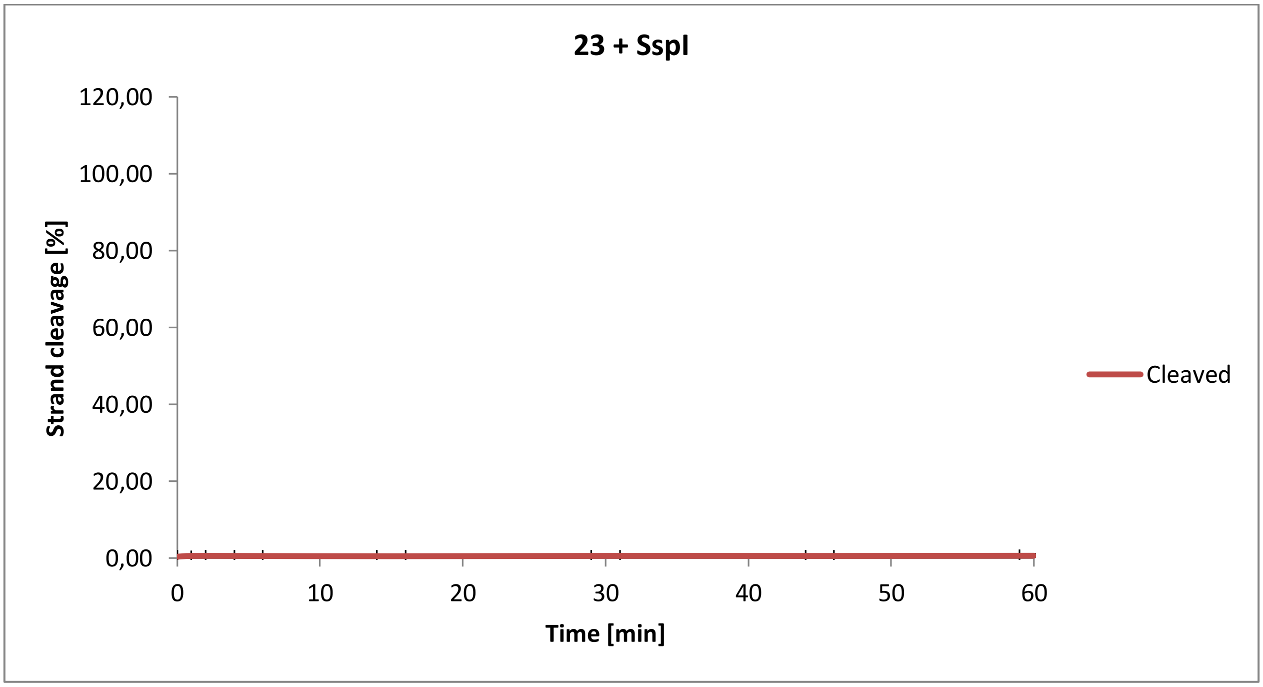

Figure S58. Cleavage of dsDNA (duplex L) by 1.5 U SspI. Quantity increase of cleaved ssDNA (strand 23) is shown.

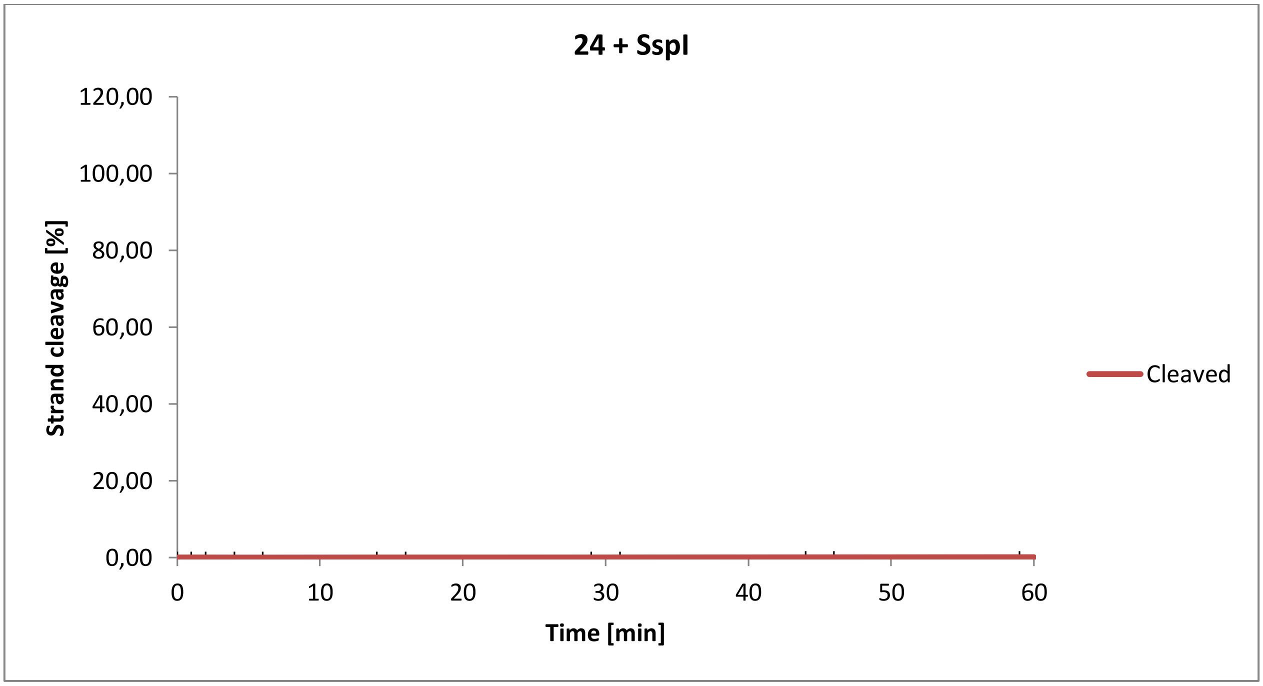

Figure S59. Cleavage of dsDNA (duplex L) by 1.5 U SspI. Quantity increase of cleaved ssDNA (strand 24) is shown.

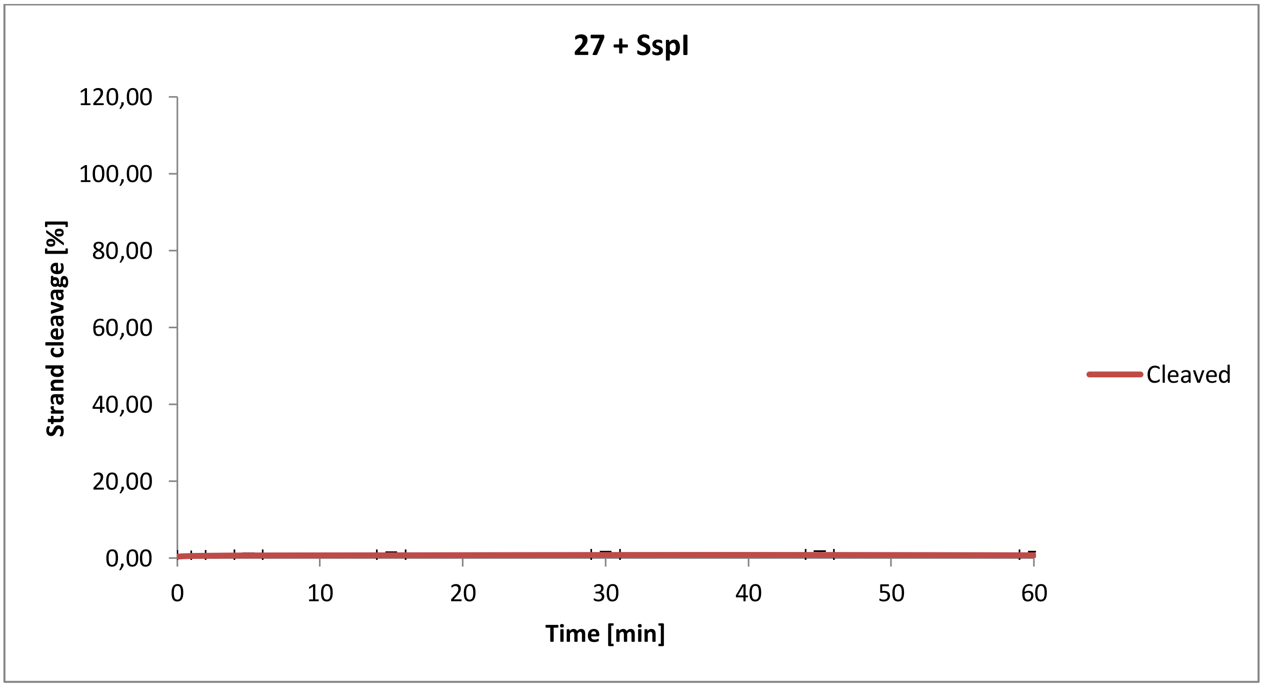

Figure S60. Cleavage of dsDNA (duplex N) by 1.5 U SspI. Quantity increase of cleaved ssDNA (strand 27) is shown.

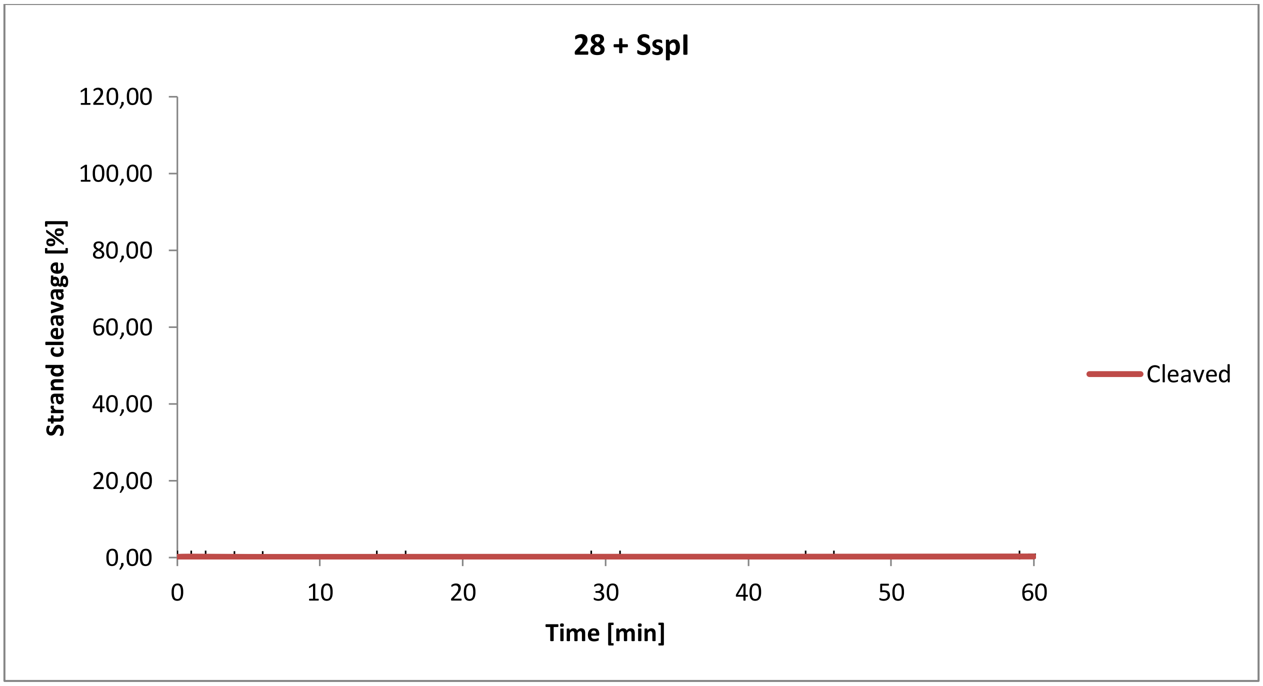

Figure S61. Cleavage of dsDNA (duplex N) by 1.5 U SspI. Quantity increase of cleaved ssDNA (strand 28) is shown.

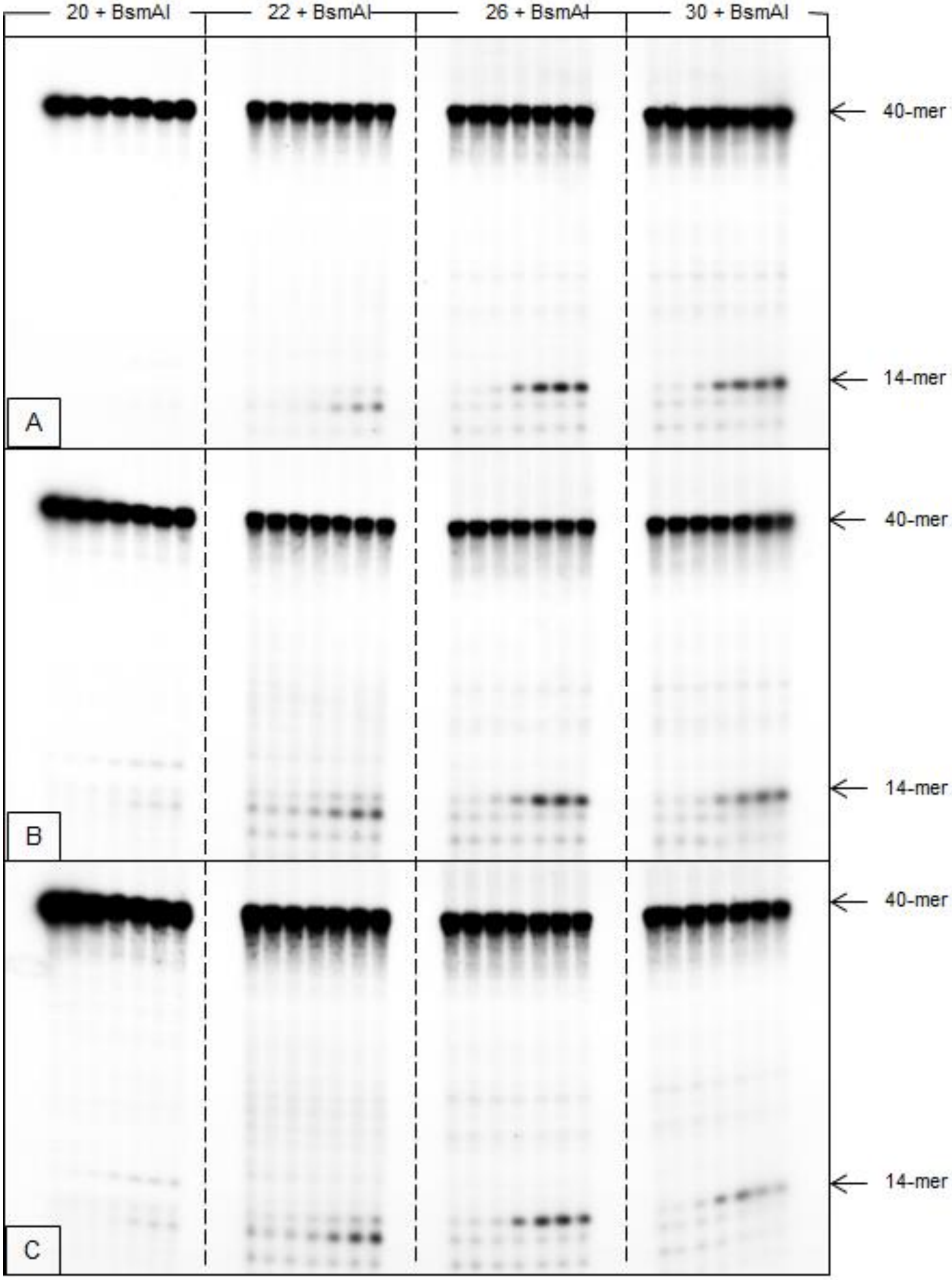

Figure S62. The autoradiograms of denaturing PAGE presenting cleavage of oligonucleotides by 0.5 U BsmAI – (A), (B) and (C) represent three repeats of assays. Lanes of bands represent following reaction times: 0, 1, 5, 15, 30, 45 and 60 min starting from the left.

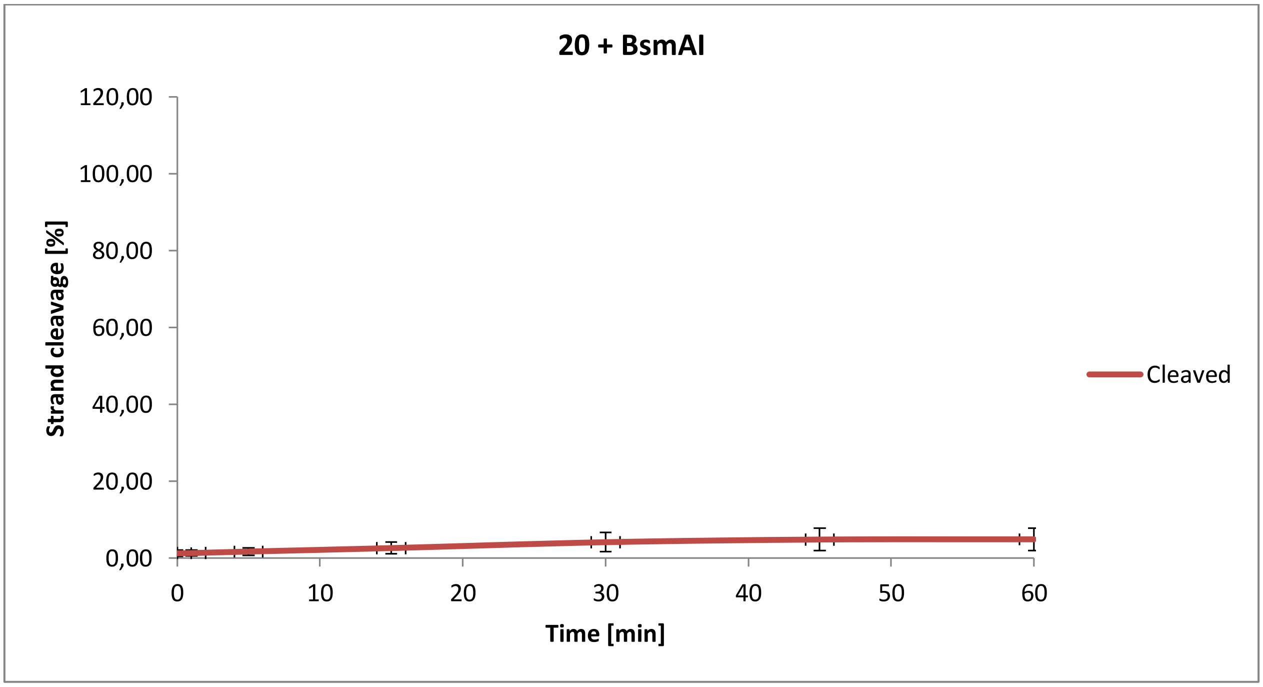

Figure S63. Cleavage of dsDNA (duplex J) by 0.5 U BsmAI. Quantity increase of cleaved ssDNA (strand 20) is shown.

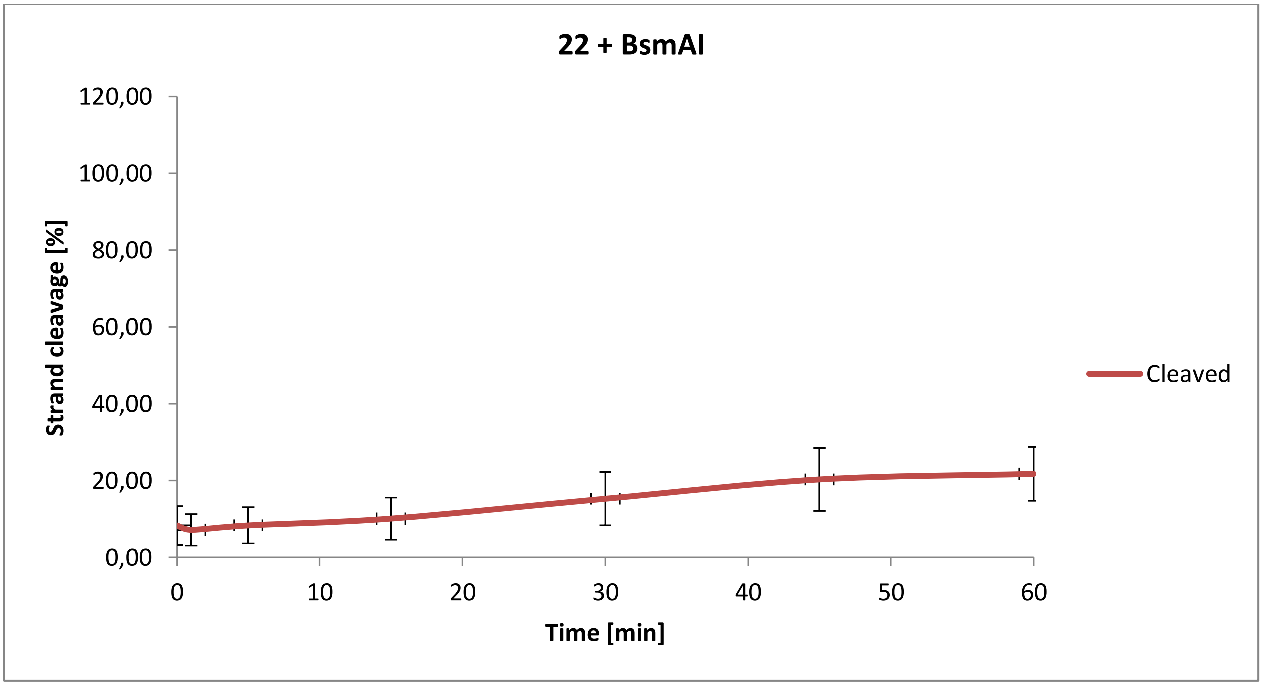

Figure S64. Cleavage of dsDNA (duplex K) by 0.5 U BsmAI. Quantity increase of cleaved ssDNA (strand 22) is shown.

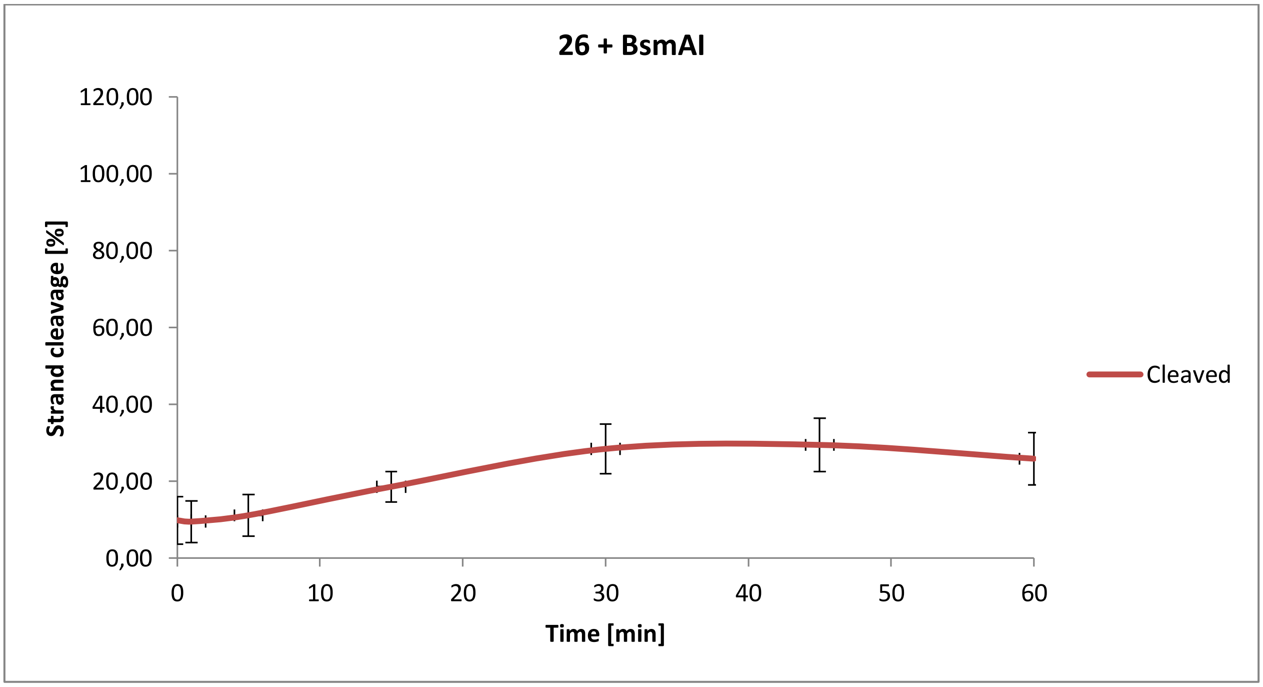

Figure S65. Cleavage of dsDNA (duplex M) by 0.5 U BsmAI. Quantity increase of cleaved ssDNA (strand 26) is shown.

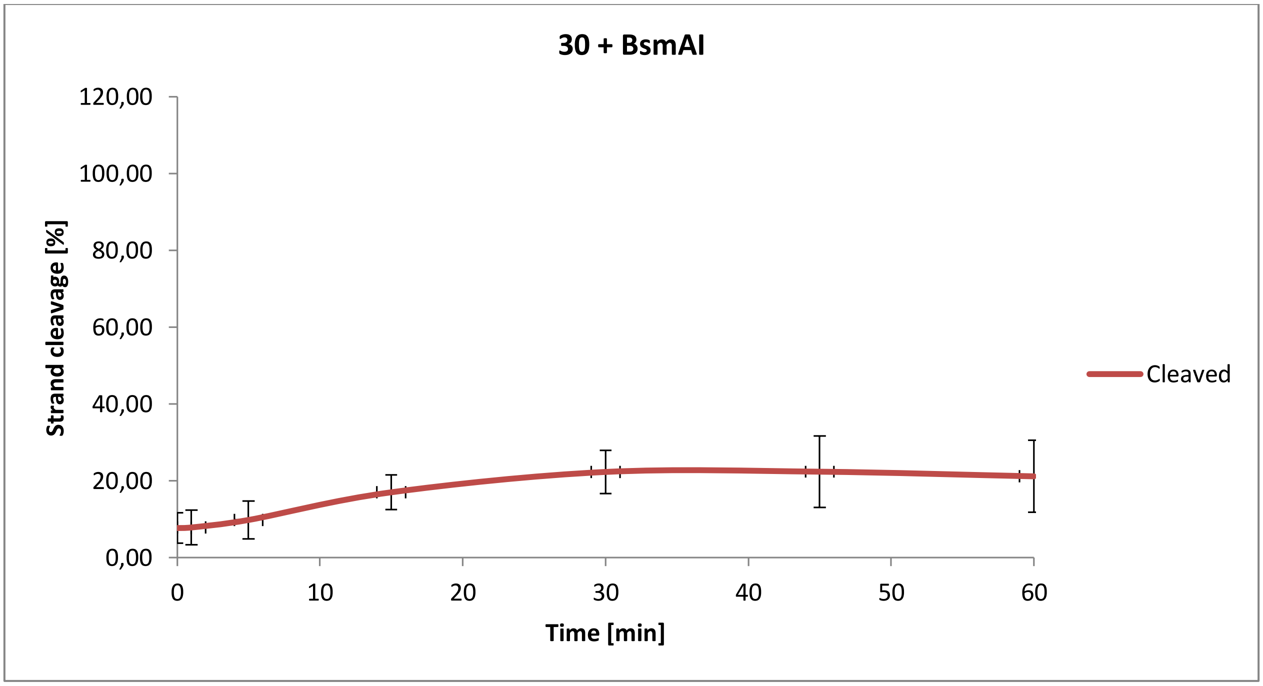

Figure S66. Cleavage of dsDNA (duplex O) by 0.5 U BsmAI. Quantity increase of cleaved ssDNA (strand 30) is shown.

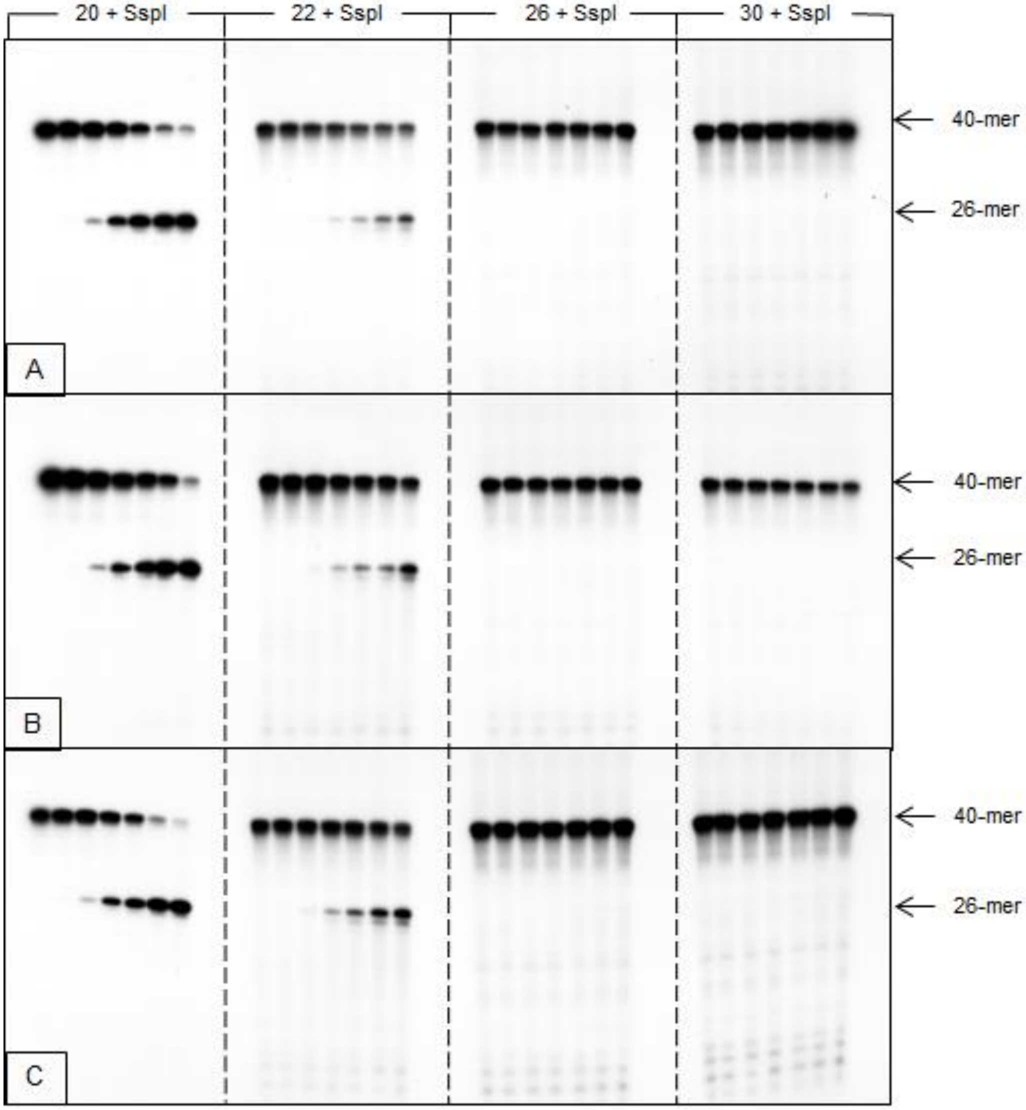

Figure S67. The autoradiograms of denaturing PAGE presenting cleavage of oligonucleotides by 1.5 U SspI – (A), (B) and (C) represent three repeats of assays. Lanes of bands represent following reaction times: 0, 1, 5, 15, 30, 45 and 60 min starting from the left.

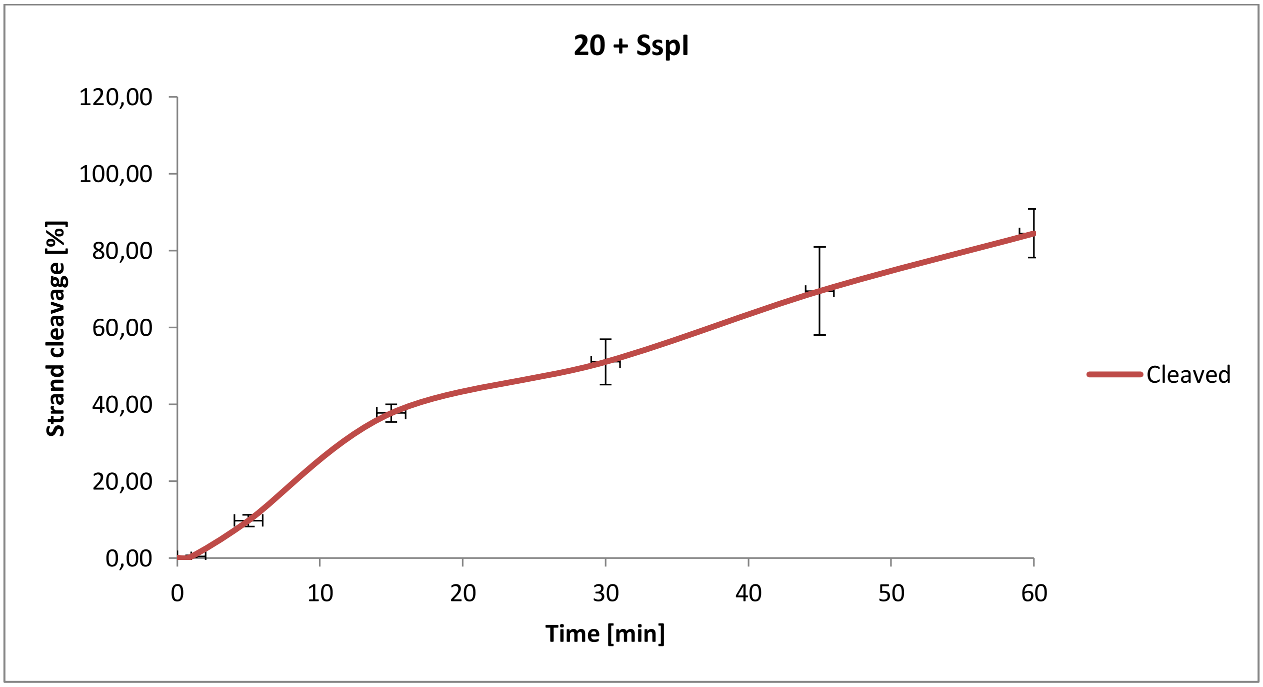

Figure S68. Cleavage of dsDNA (duplex J) by 1.5 U SspI. Quantity increase of cleaved ssDNA (strand 20) is shown.

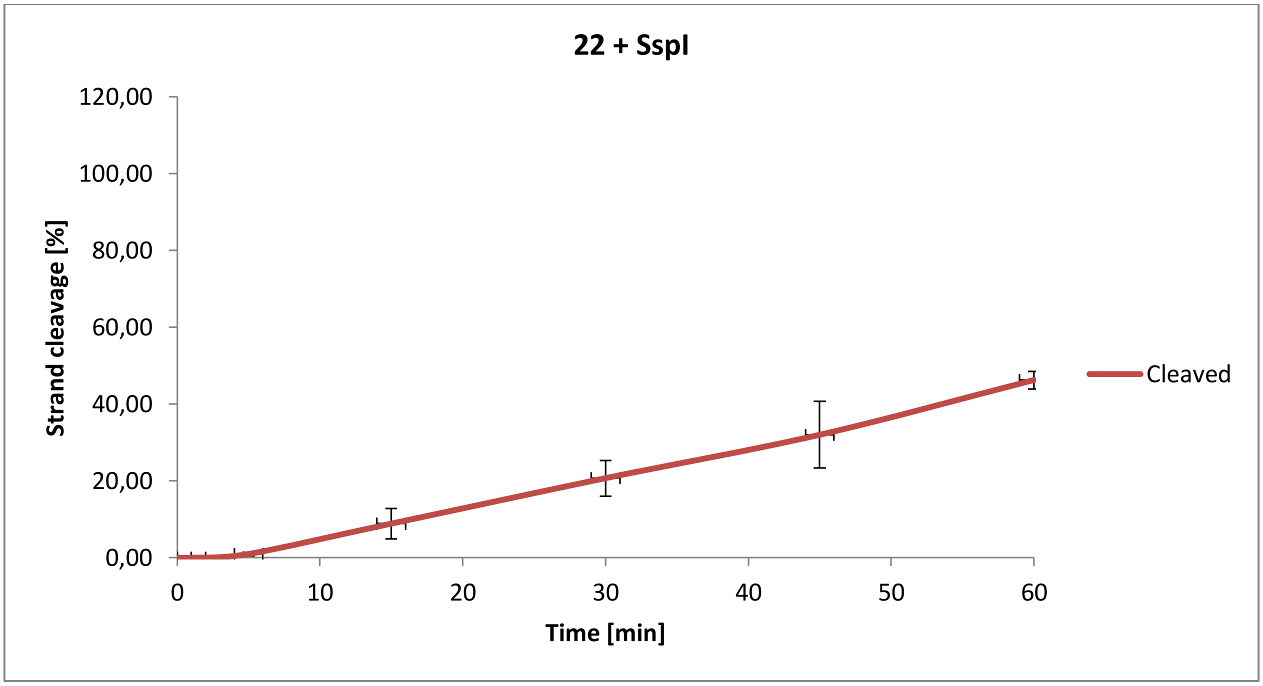

Figure S69. Cleavage of dsDNA (duplex K) by 1.5 U SspI. Quantity increase of cleaved ssDNA (strand 22) is shown.

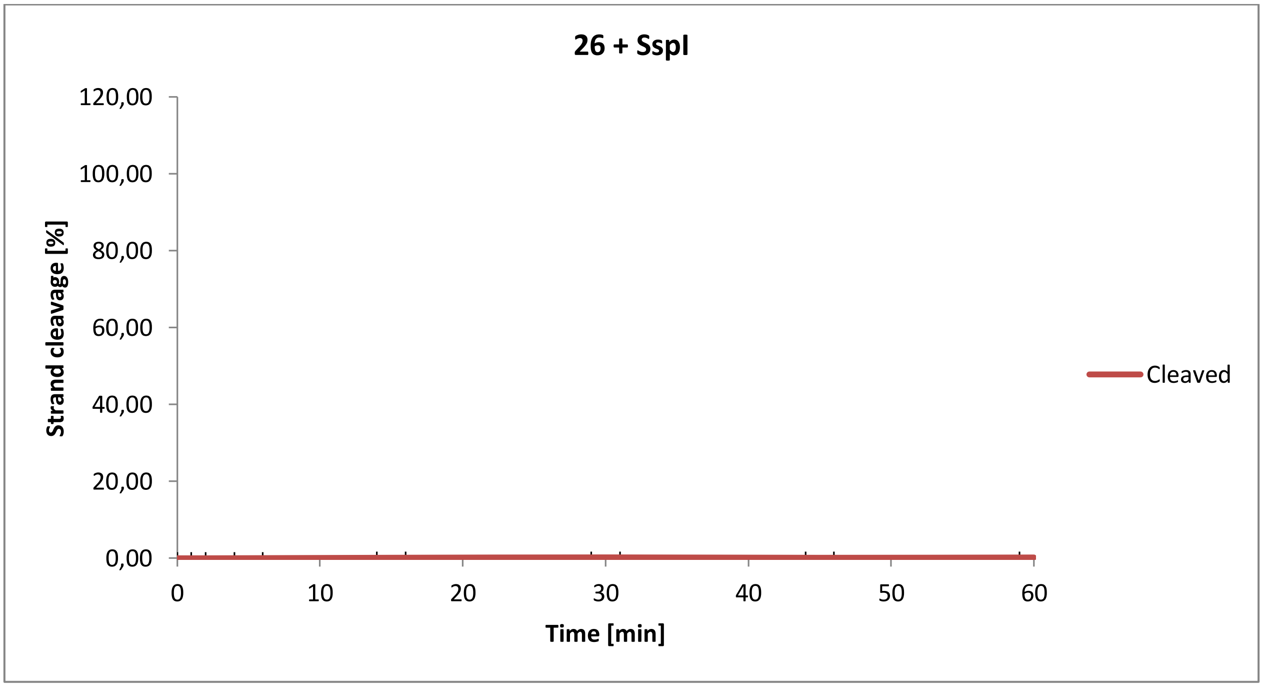

Figure S70. Cleavage of dsDNA (duplex M) by 1.5 U SspI. Quantity increase of cleaved ssDNA (strand 26) is shown.

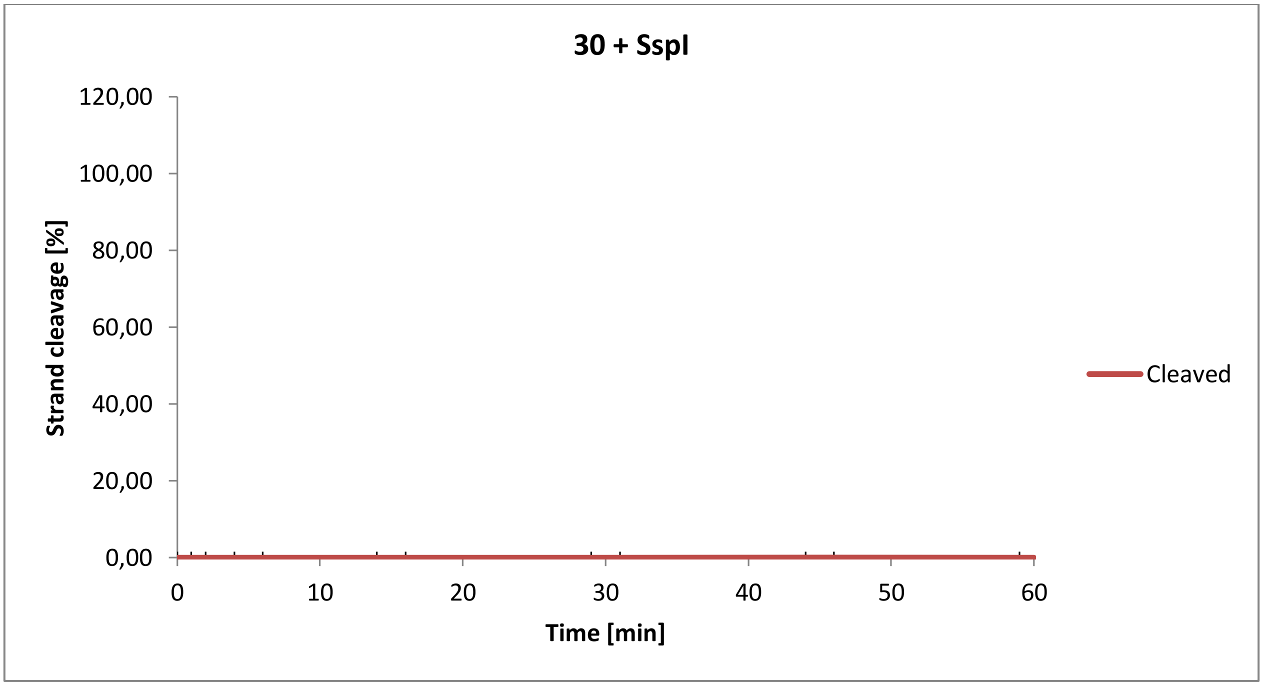

Figure S71. Cleavage of dsDNA (duplex O) by 1.5 U SspI. Quantity increase of cleaved ssDNA (strand 30) is shown.

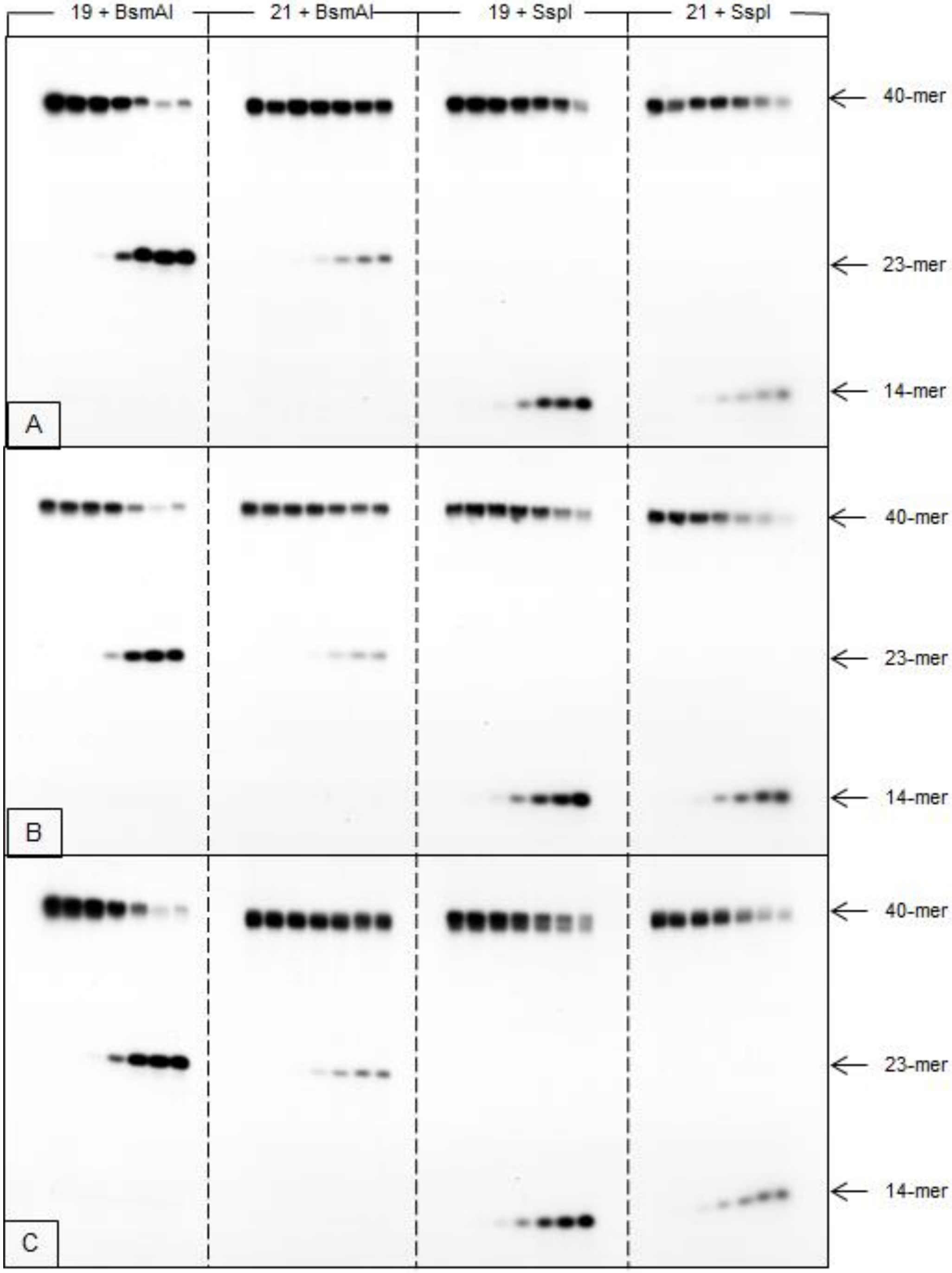

Figure S72. The autoradiograms of denaturing PAGE presenting cleavage of oligonucleotides by 0.5 U BsmAI or 1.5 U SspI – (A), (B) and (C) represent three repeats of assays. Lanes of bands represent following reaction times: 0, 1, 5, 15, 30, 45 and 60 min starting from the left.

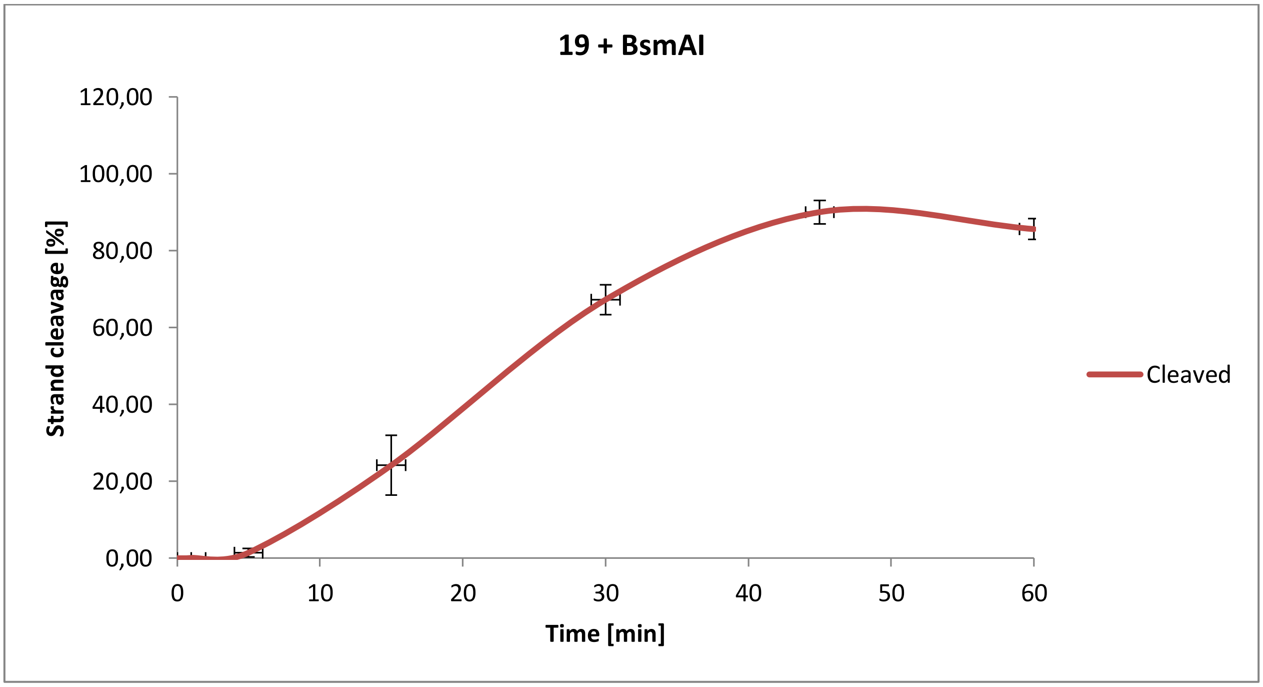

Figure S73. Cleavage of dsDNA (duplex J) by 0.5 U BsmAI. Quantity increase of cleaved ssDNA (strand 19) is shown.

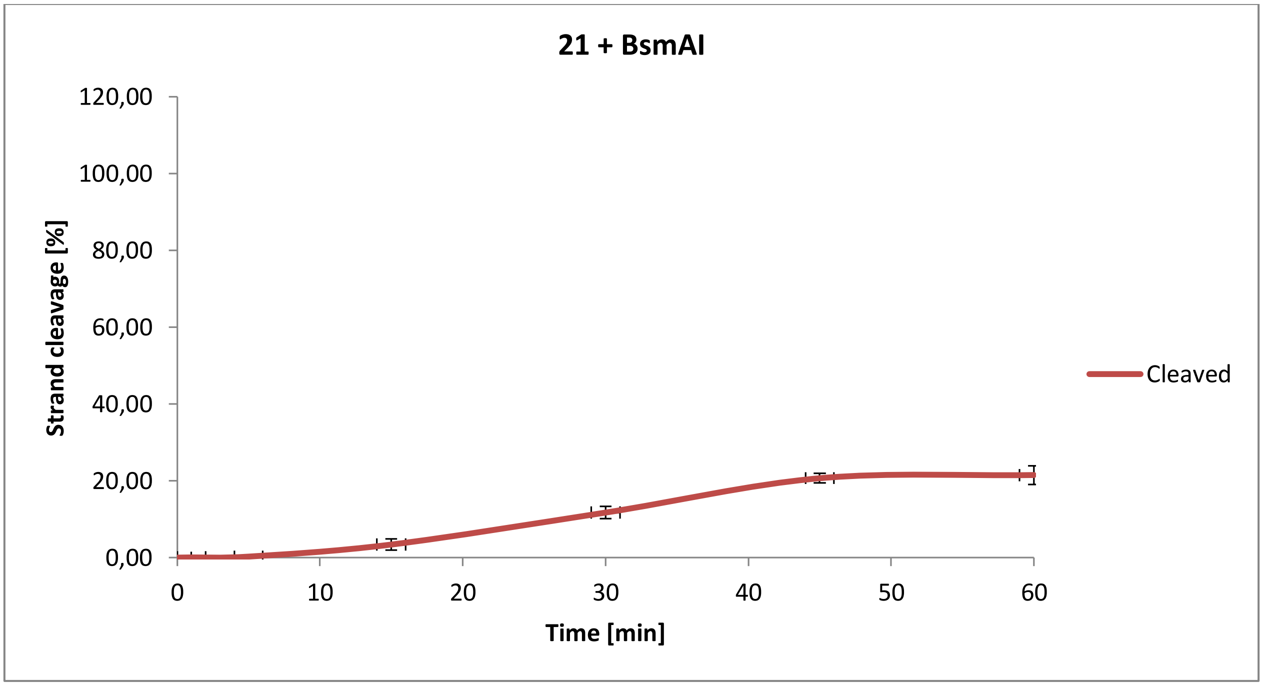

Figure S74. Cleavage of dsDNA (duplex K) by 0.5 U BsmAI. Quantity increase of cleaved ssDNA (strand 21) is shown.

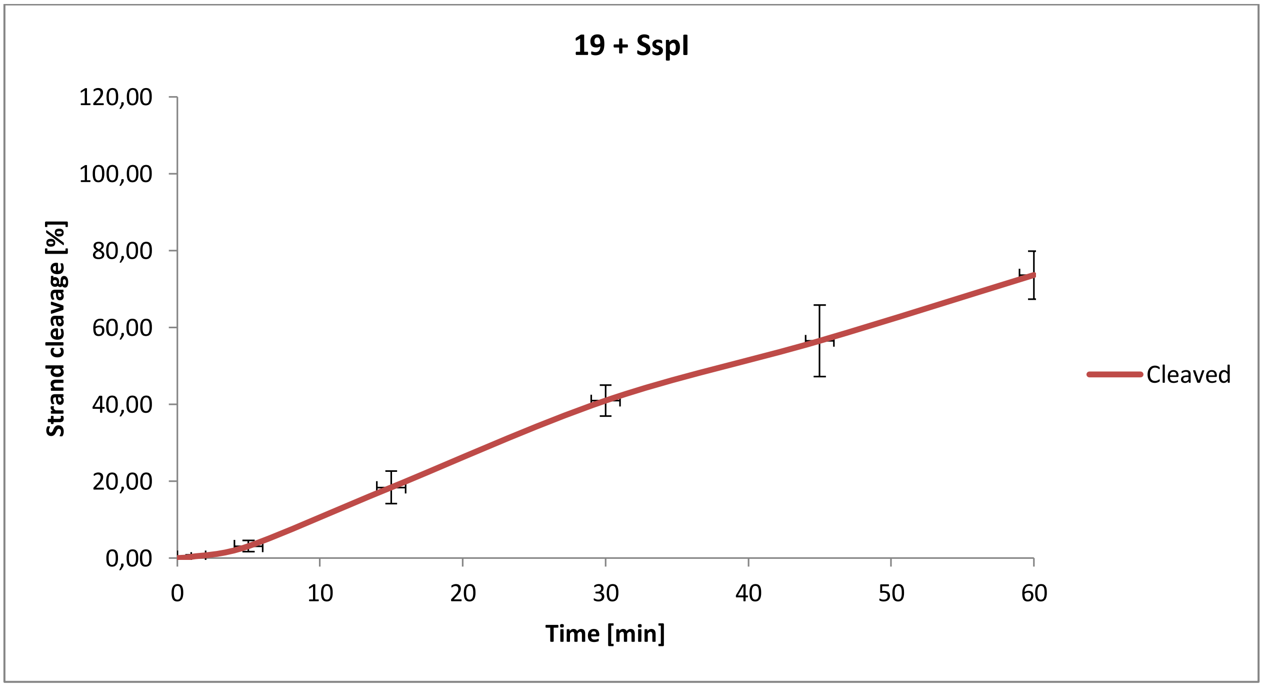

Figure S75. Cleavage of dsDNA (duplex J) by 1.5 U SspI. Quantity increase of cleaved ssDNA (strand 19) is shown.

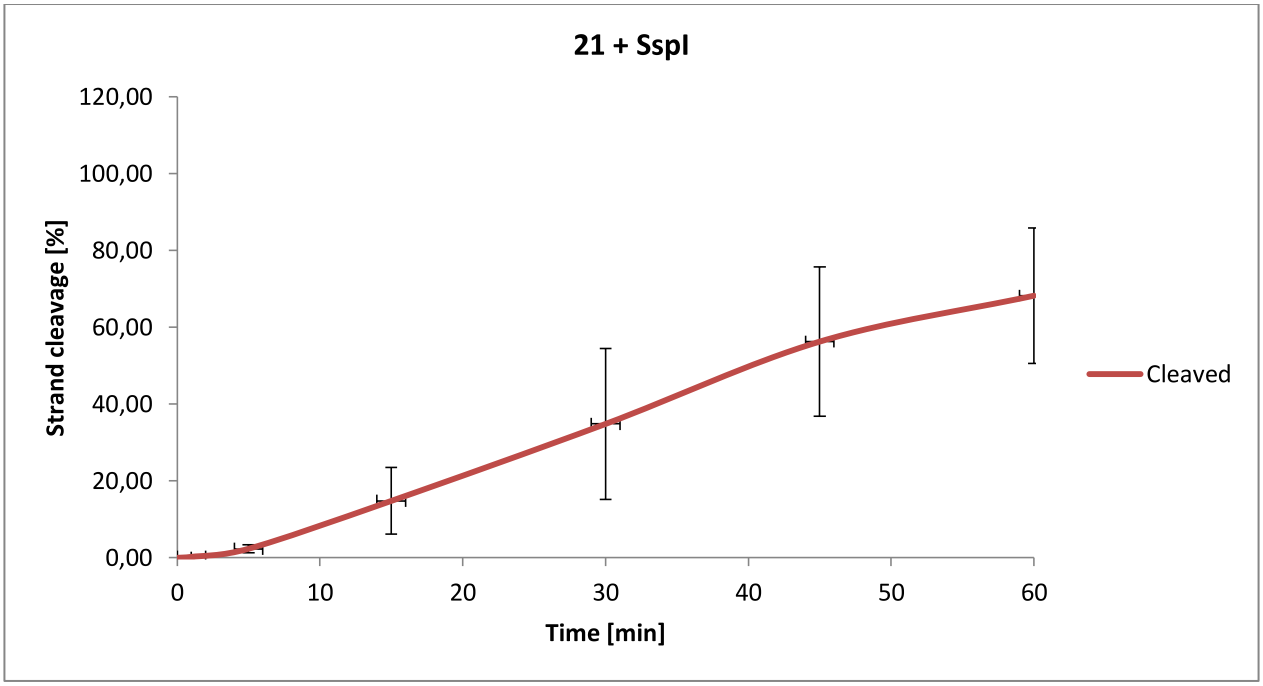

Figure S76. Cleavage of dsDNA (duplex K) by 1.5 U SspI. Quantity increase of cleaved ssDNA (strand 21) is shown.

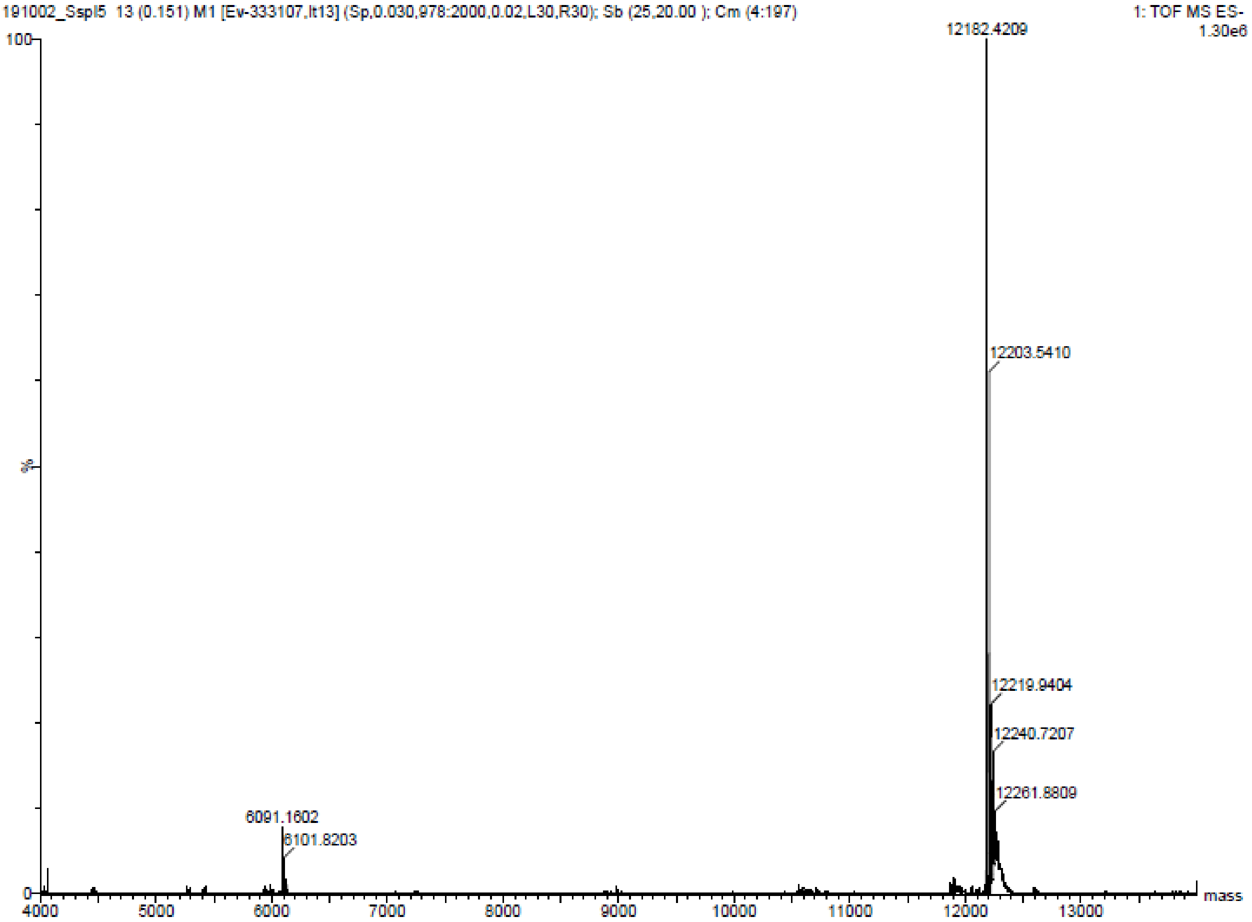

Figure S77. Mass spectrum of Matrix 5'.

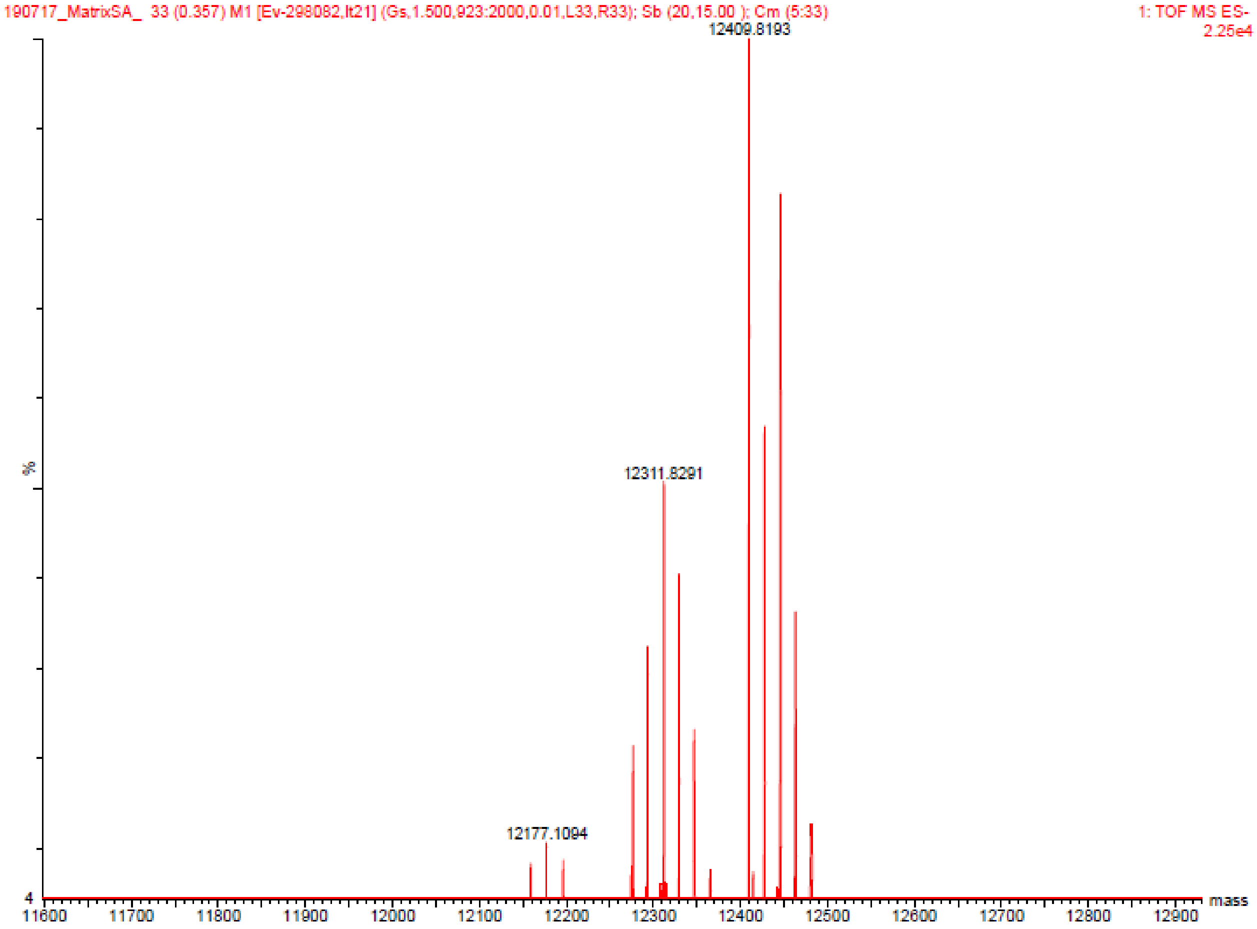

Figure S78. Mass spectrum of Matrix 3'.

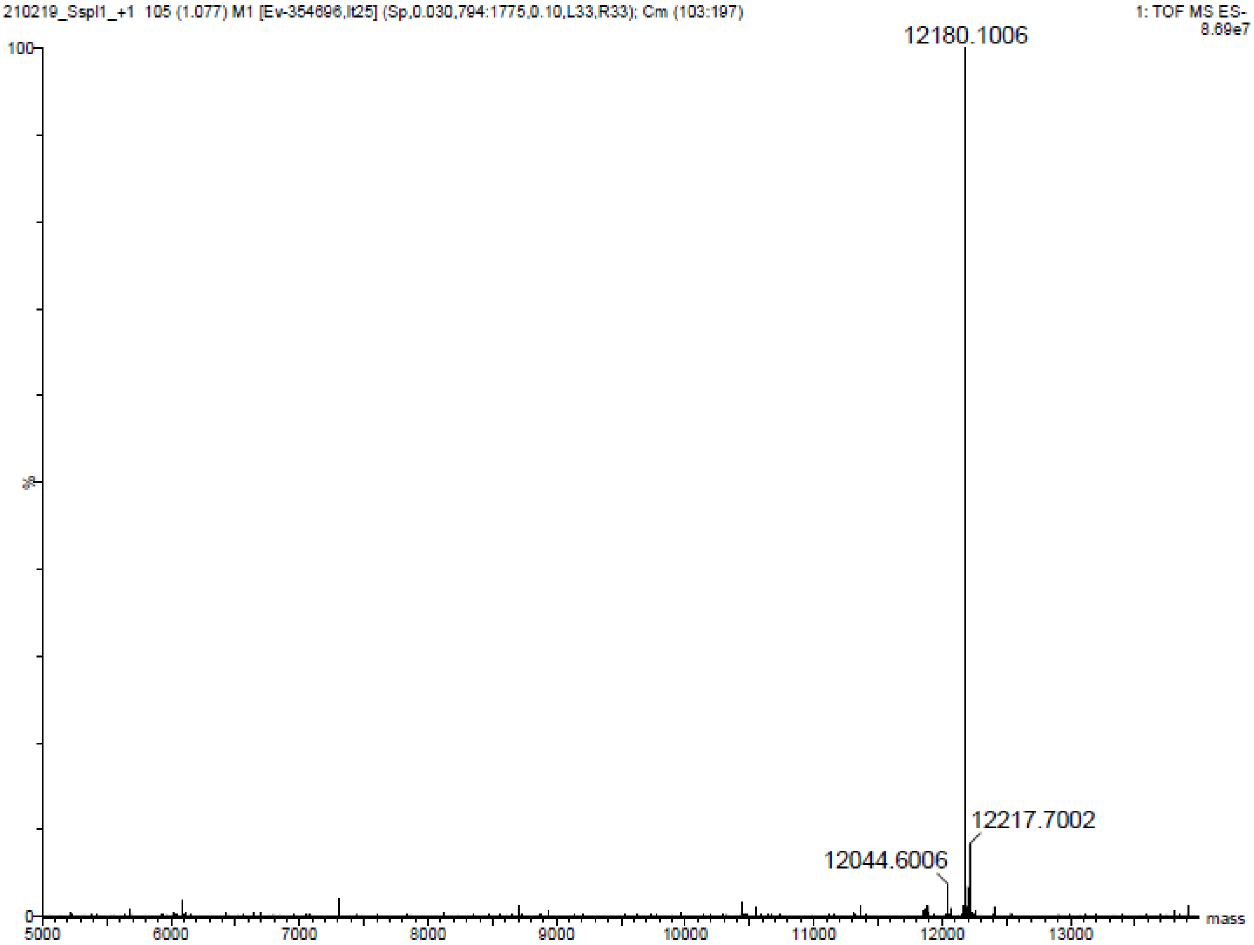

Figure S79. Mass spectrum of sequence 1.

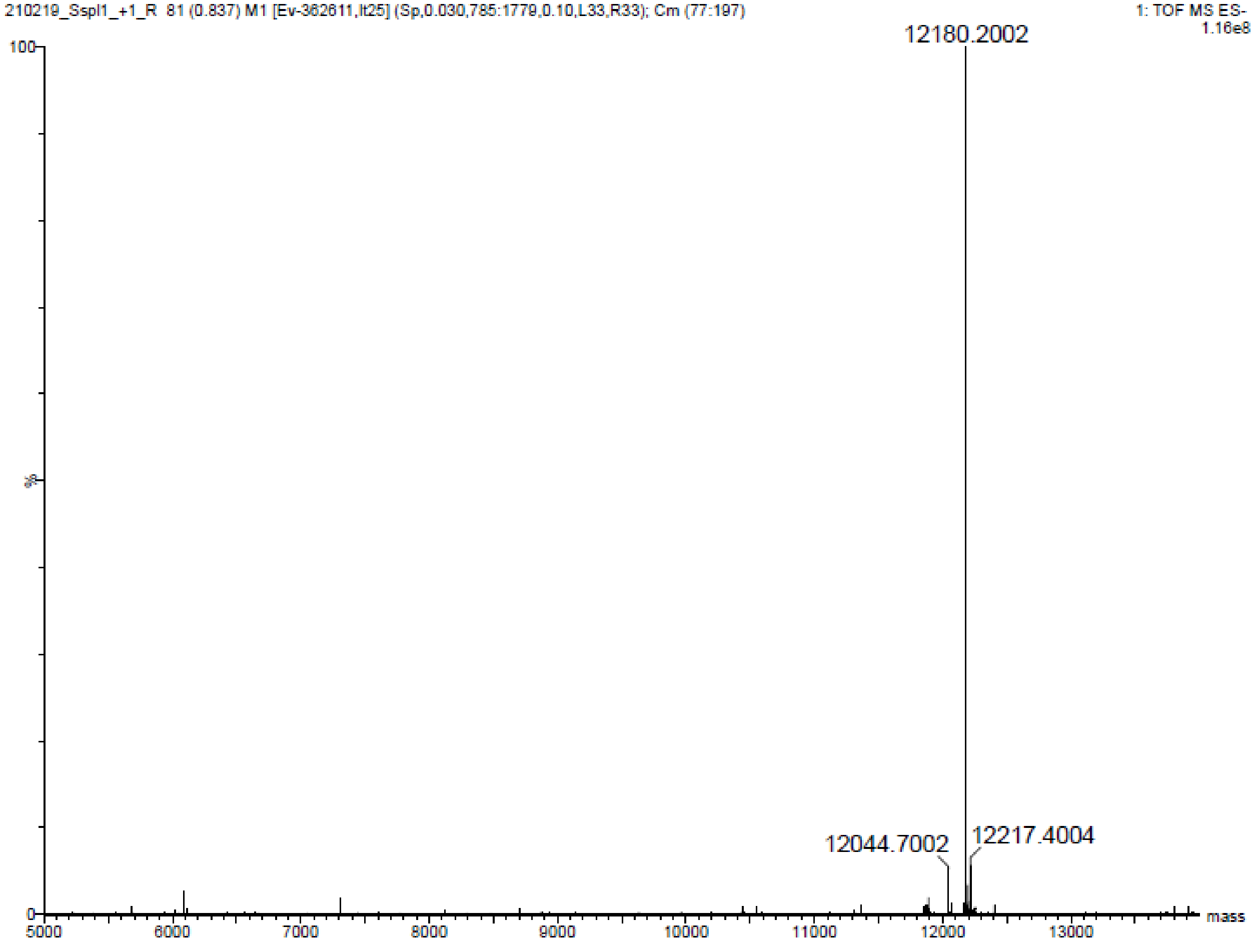

Figure S80. Mass spectrum of sequence 2.

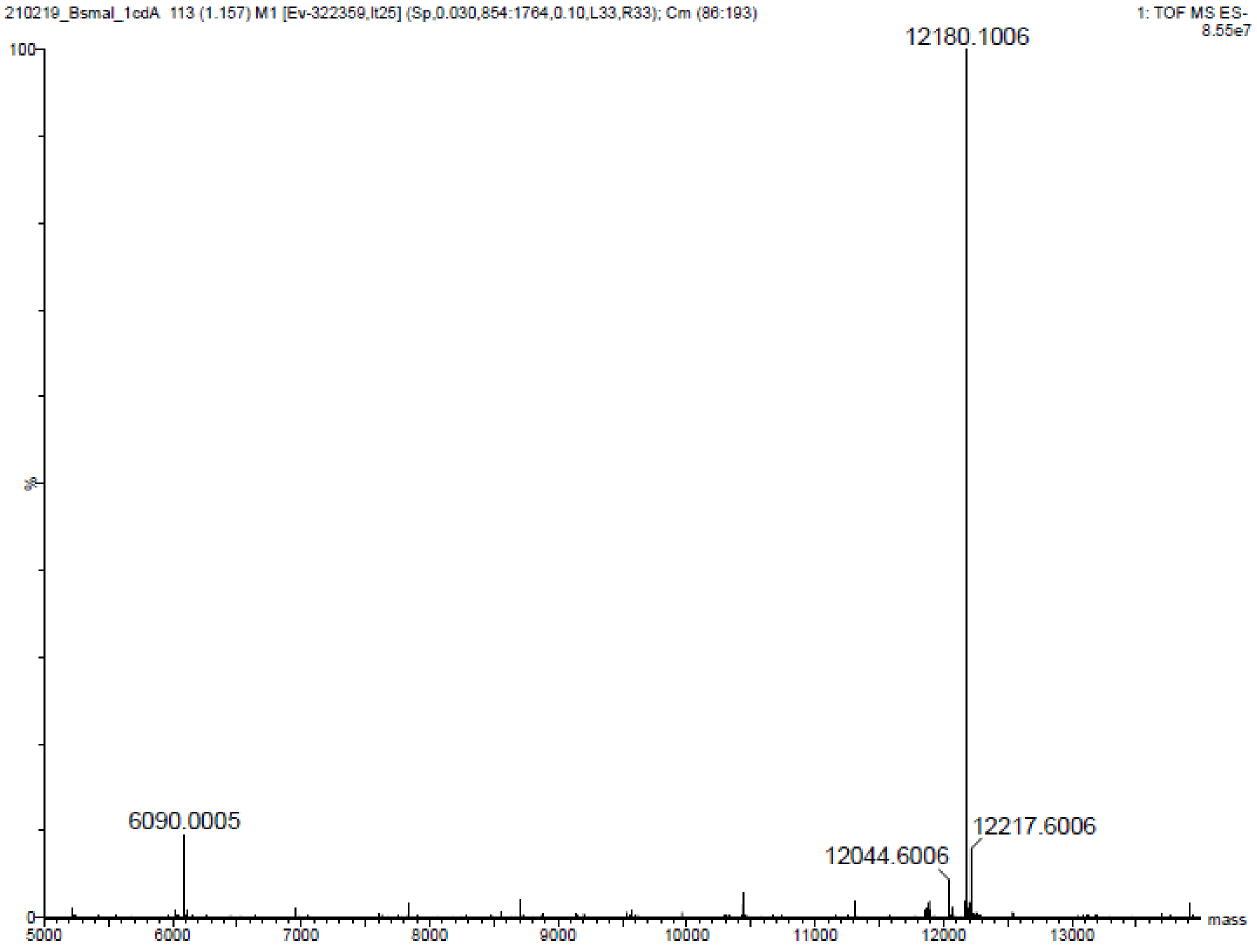

Figure S81. Mass spectrum of sequence 3.

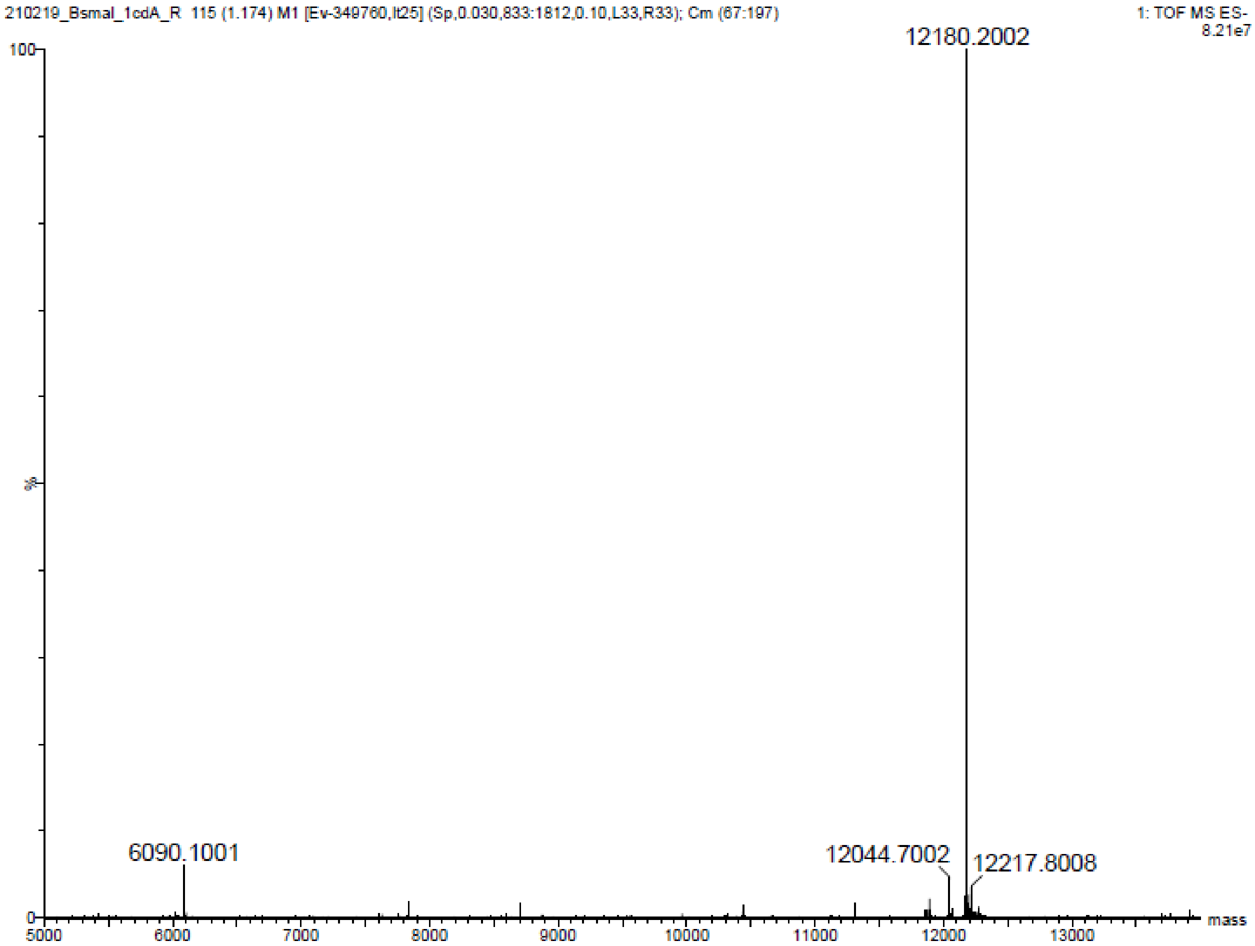

Figure S82. Mass spectrum of sequence 4.

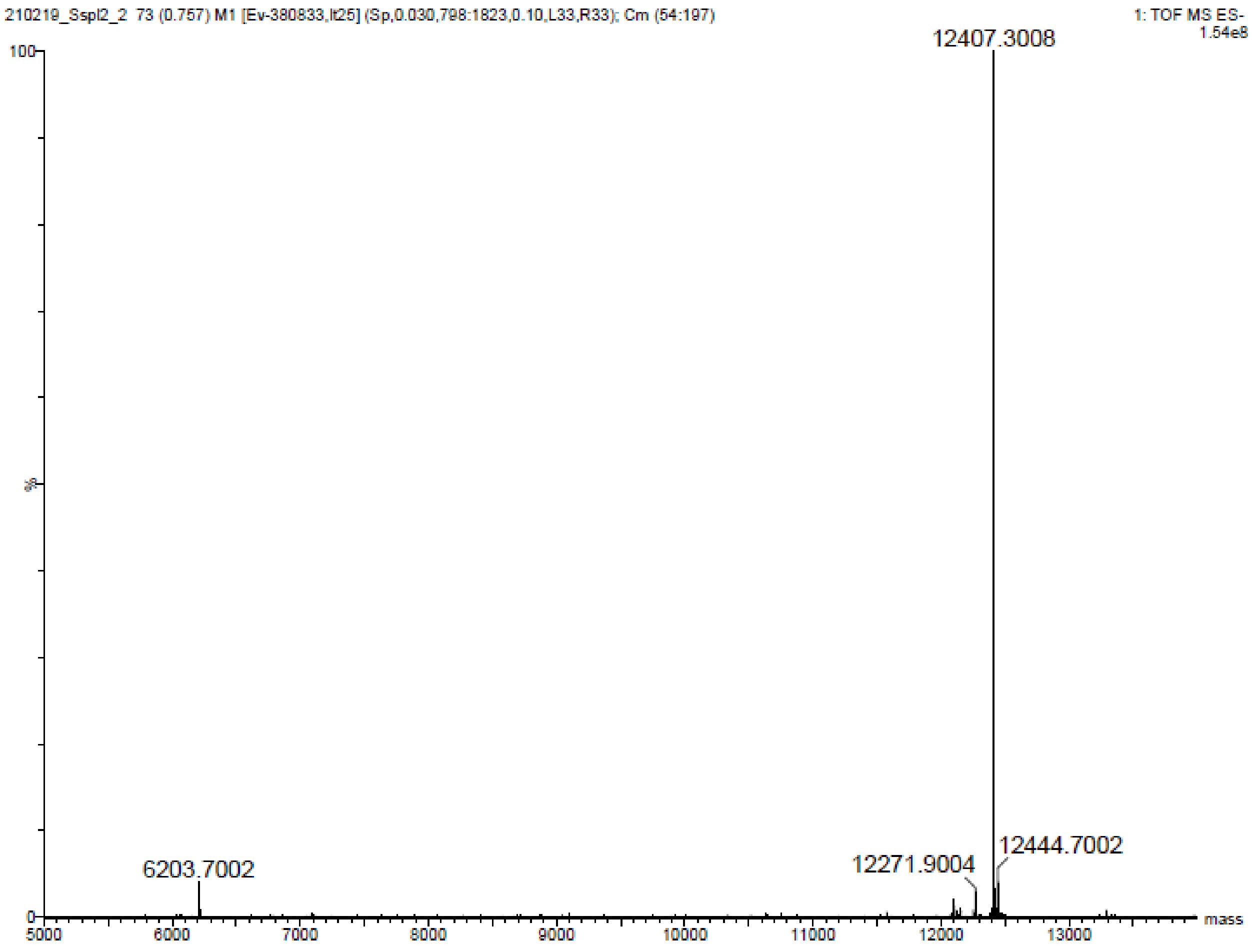

Figure S83. Mass spectrum of sequence 5.

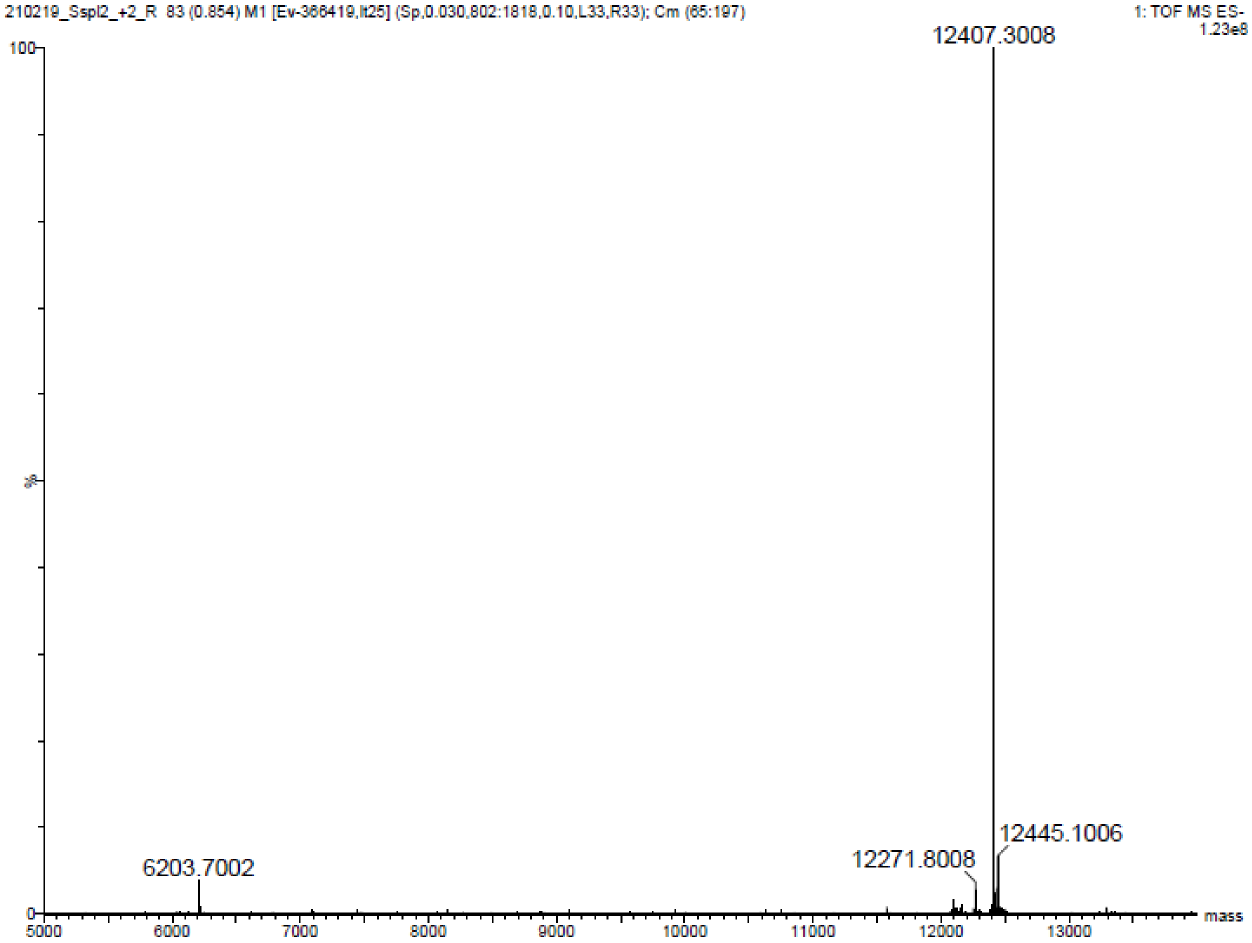

Figure S84. Mass spectrum of sequence 6.

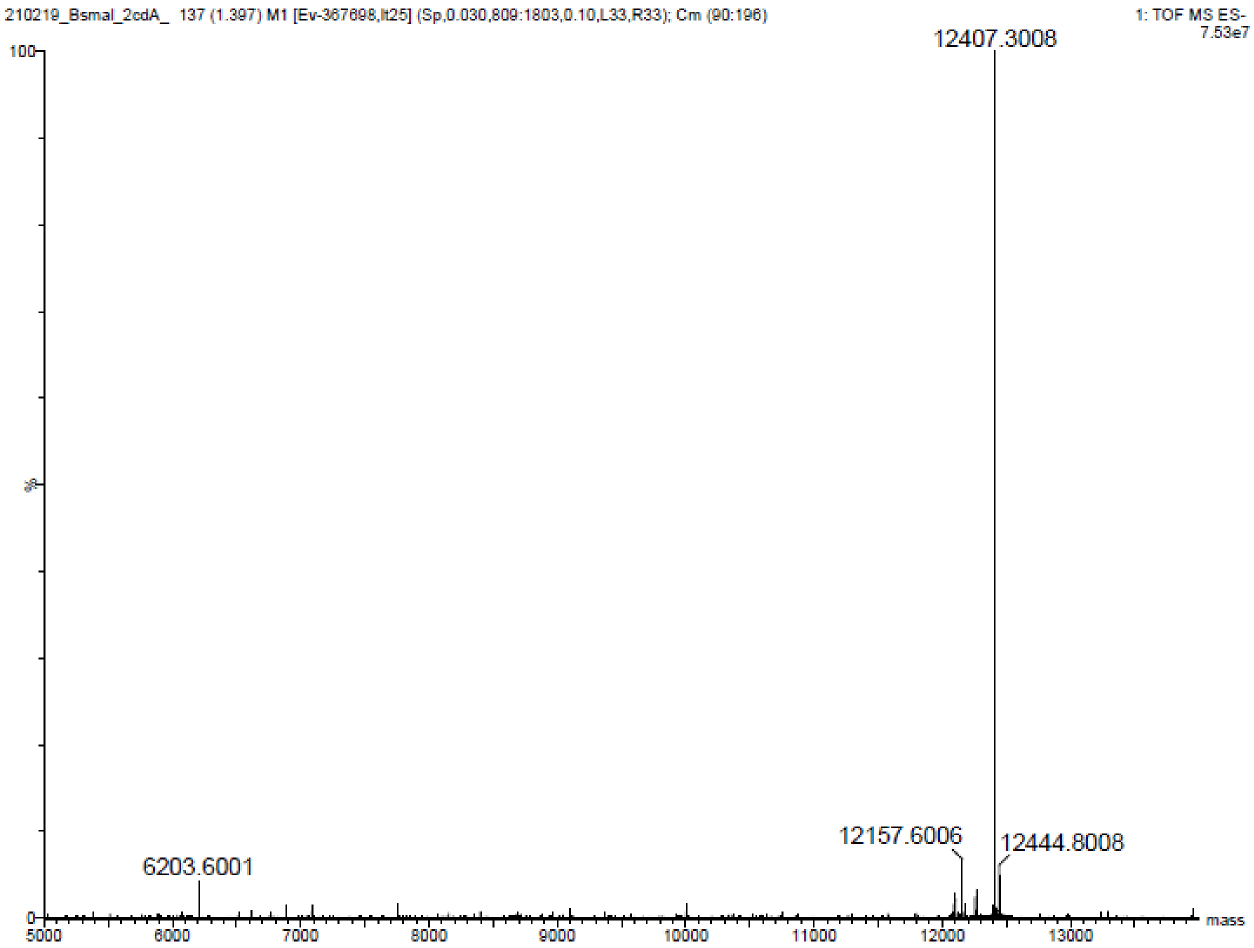

Figure S85. Mass spectrum of sequence 7.

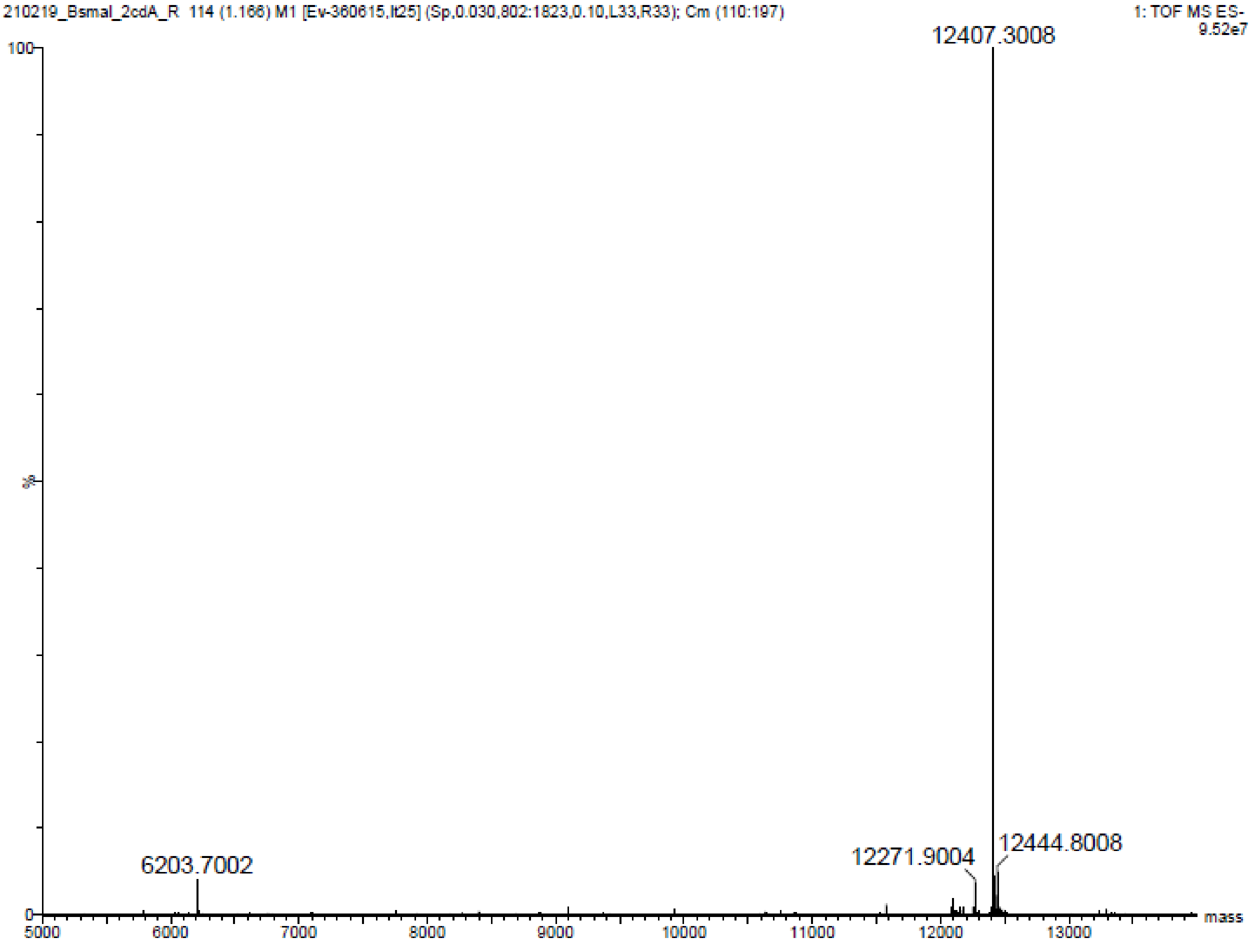

Figure S86. Mass spectrum of sequence 8.

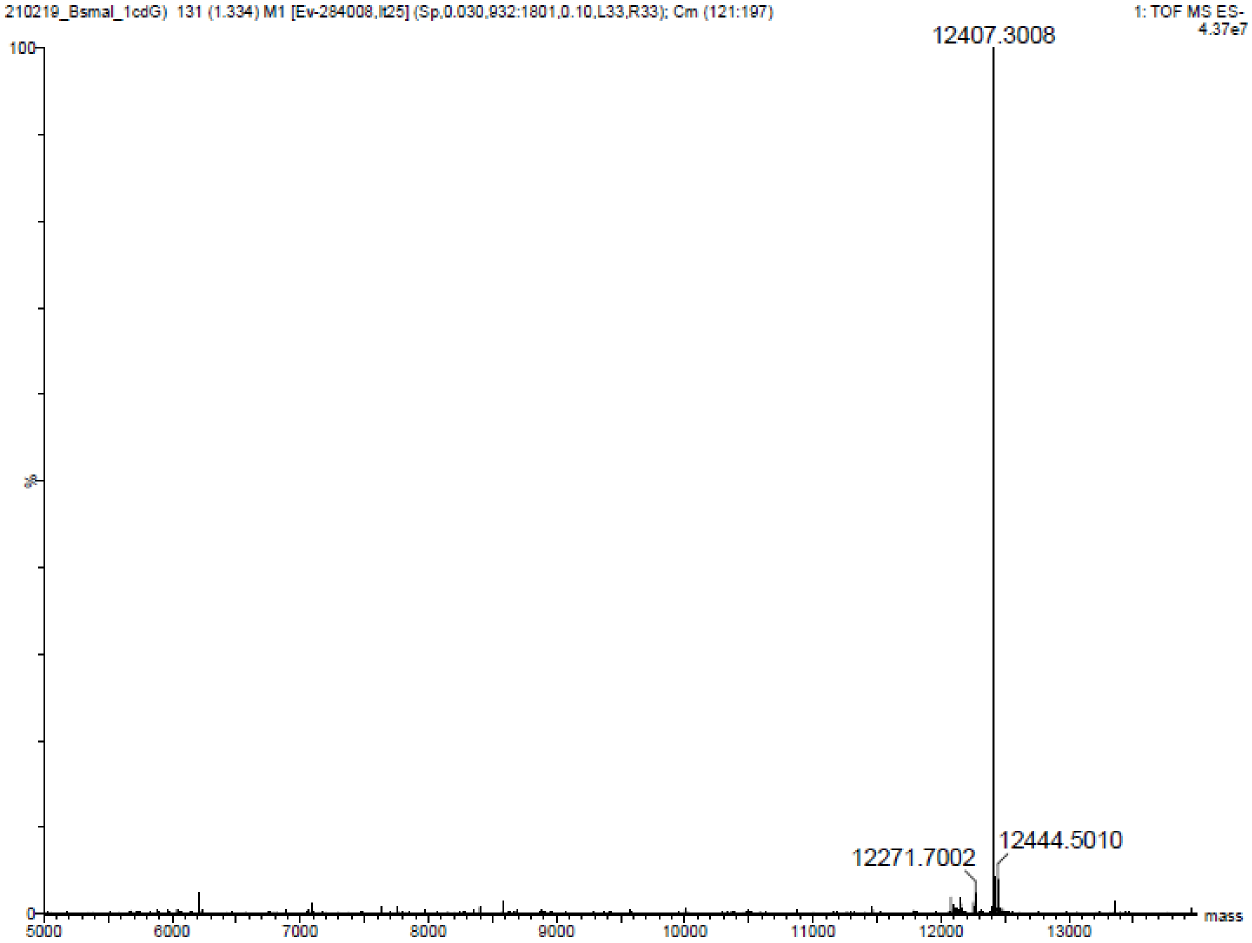

Figure S87. Mass spectrum of sequence 9.

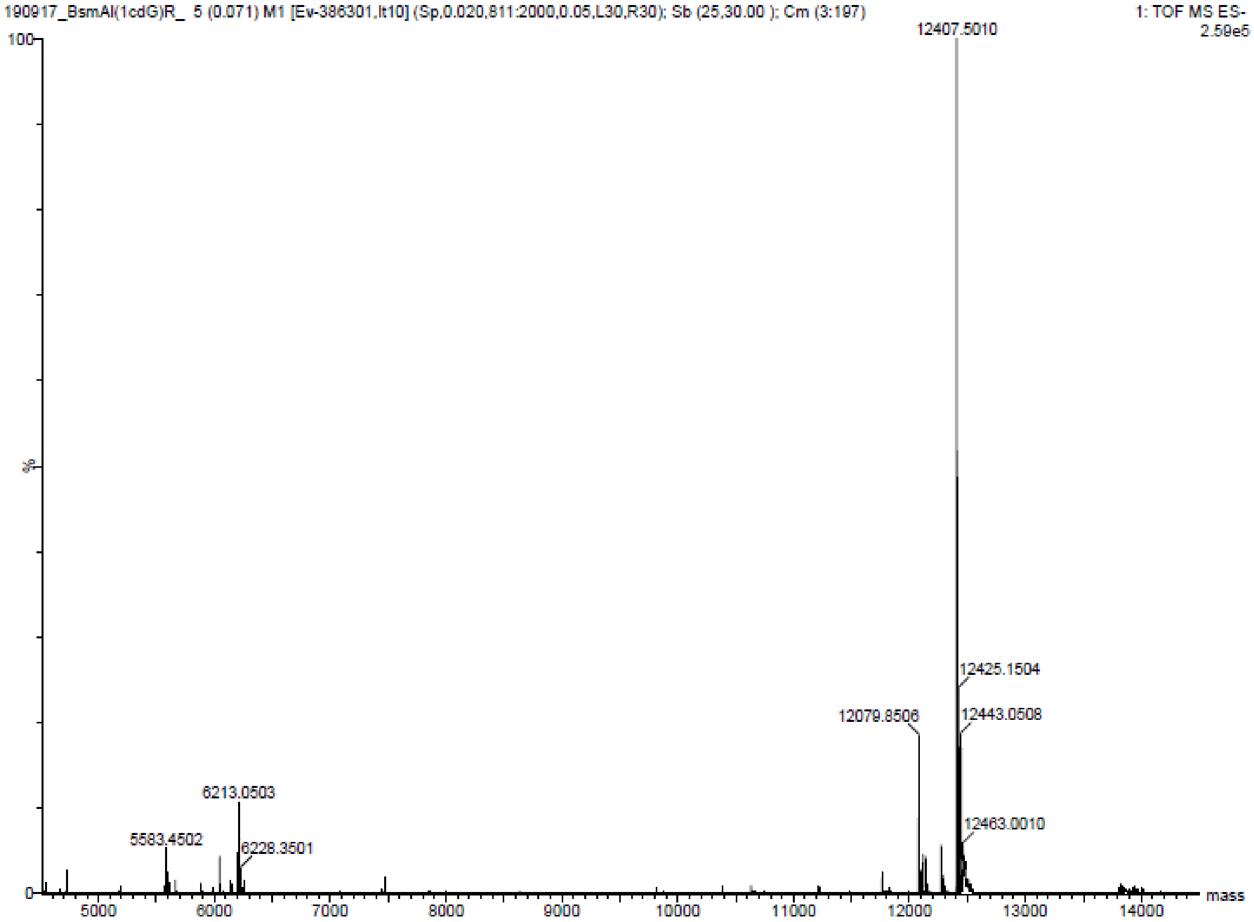

Figure S88. Mass spectrum of sequence 10.

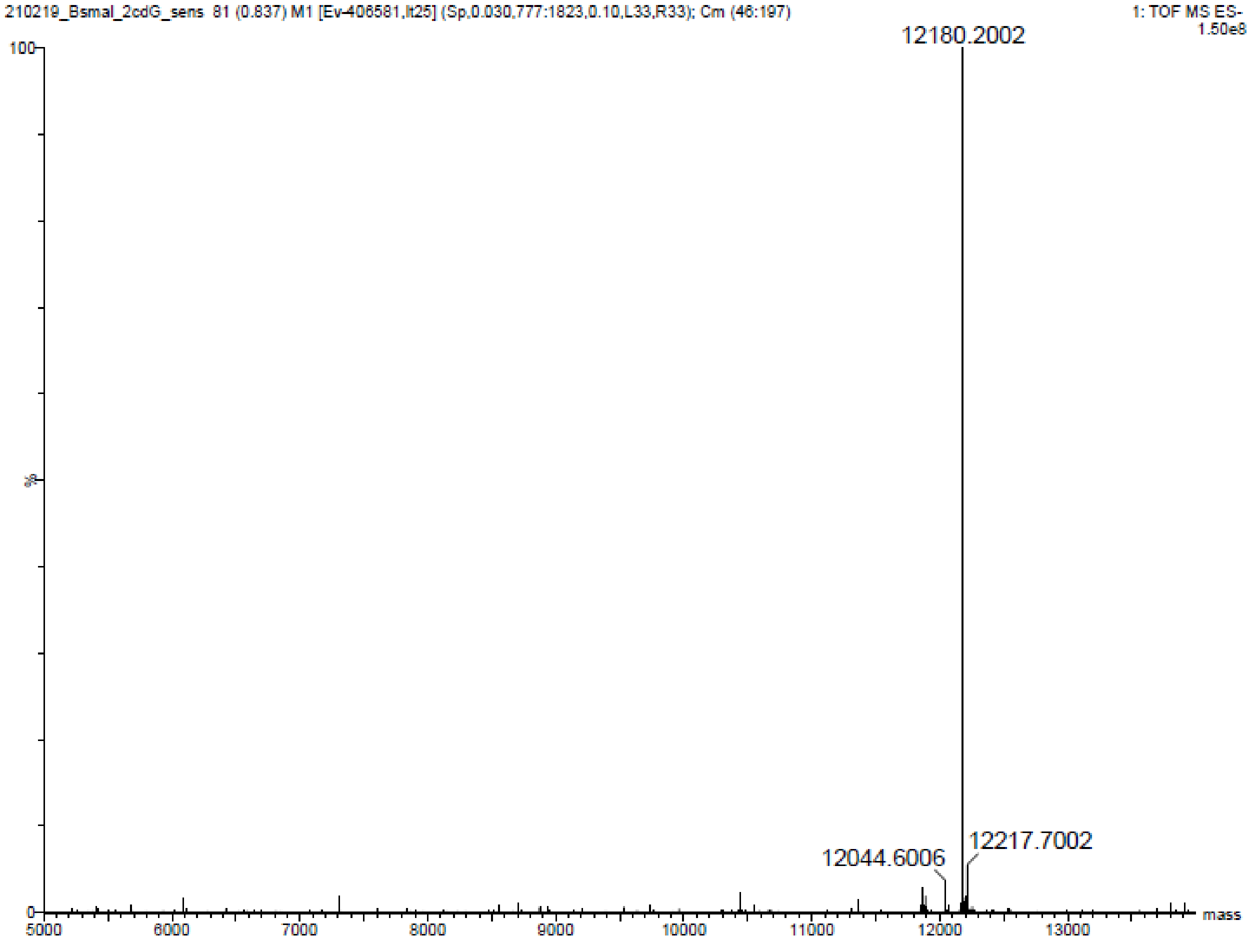

Figure S89. Mass spectrum of sequence 11.

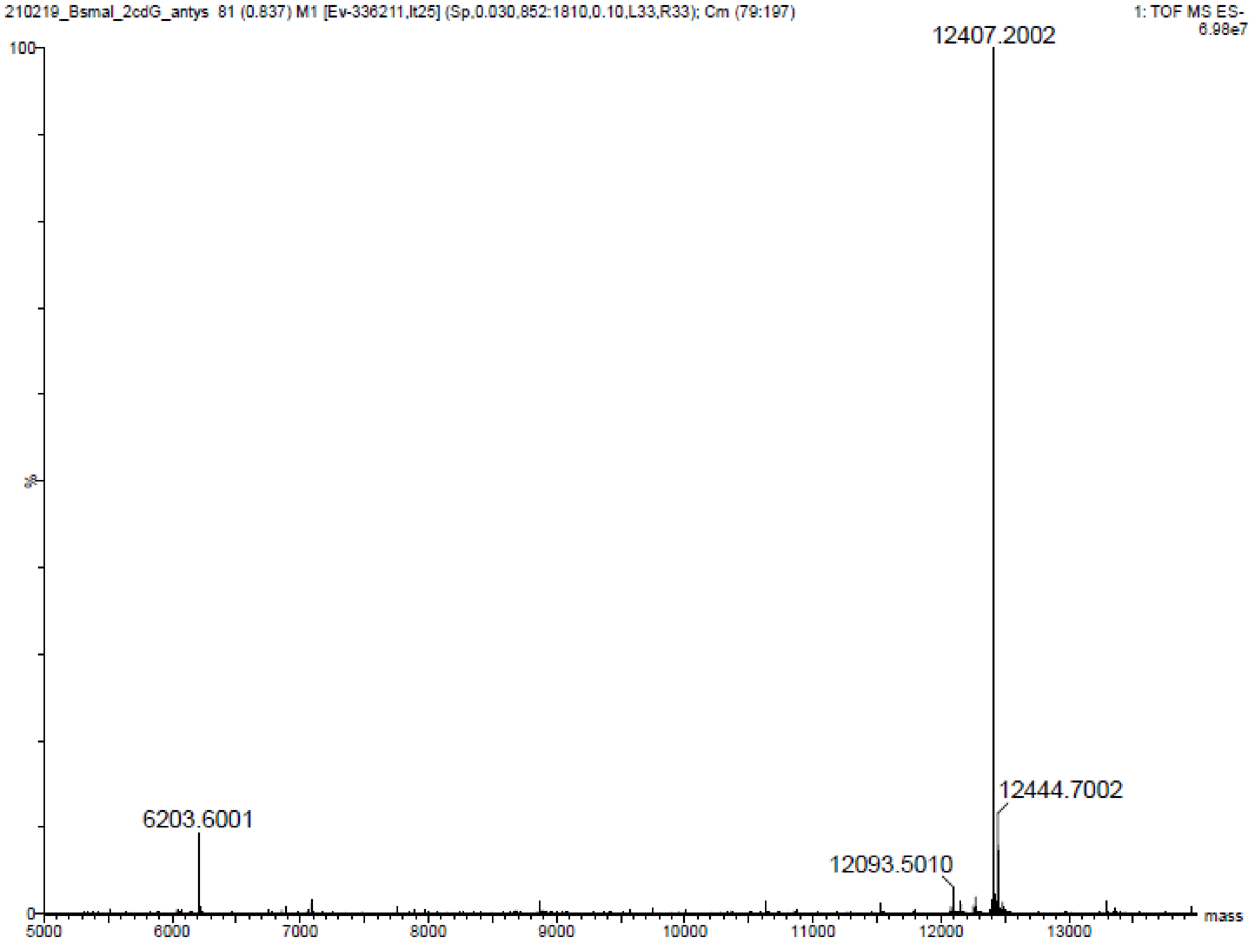

Figure S90. Mass spectrum of sequence 12.

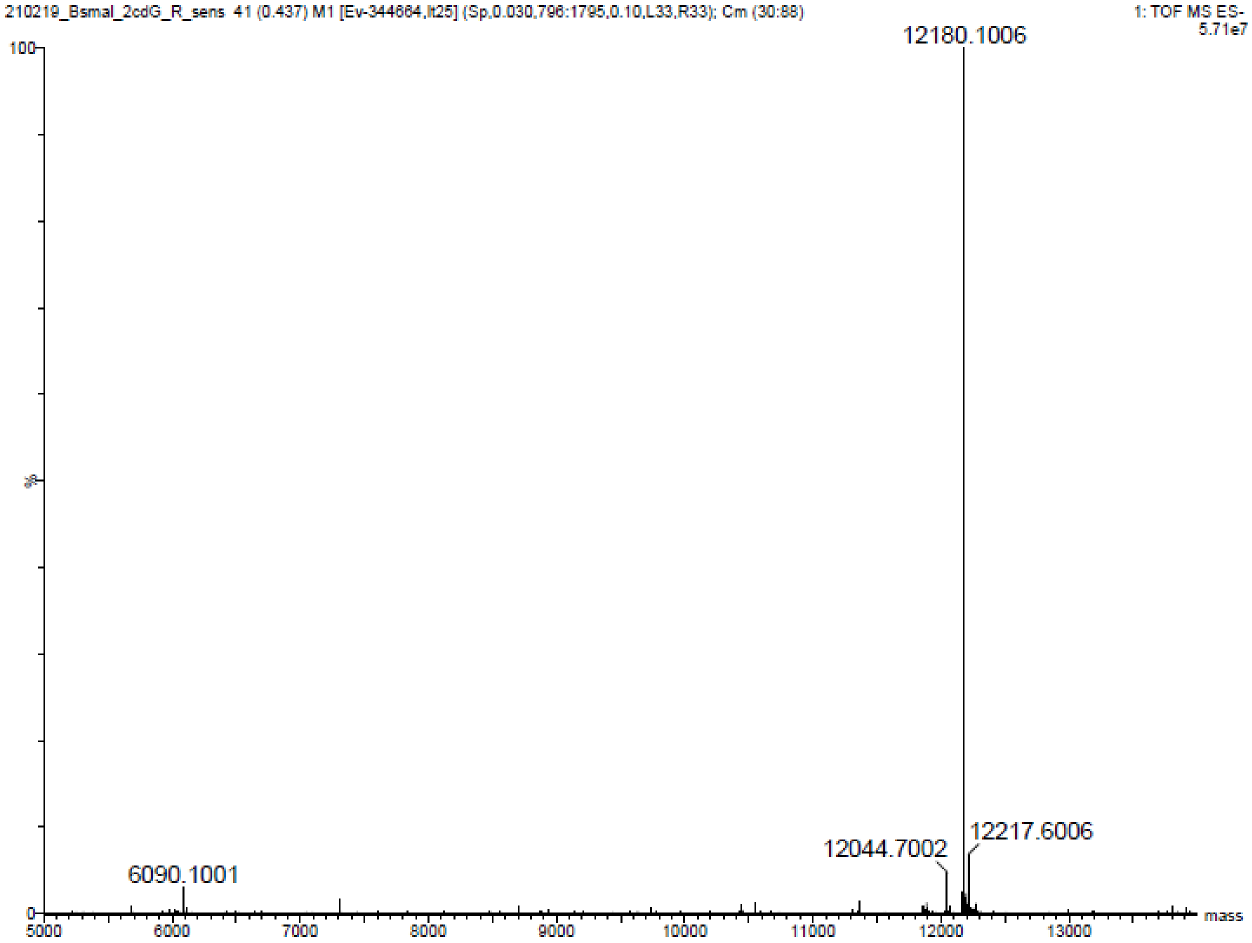

Figure S91. Mass spectrum of sequence 13.

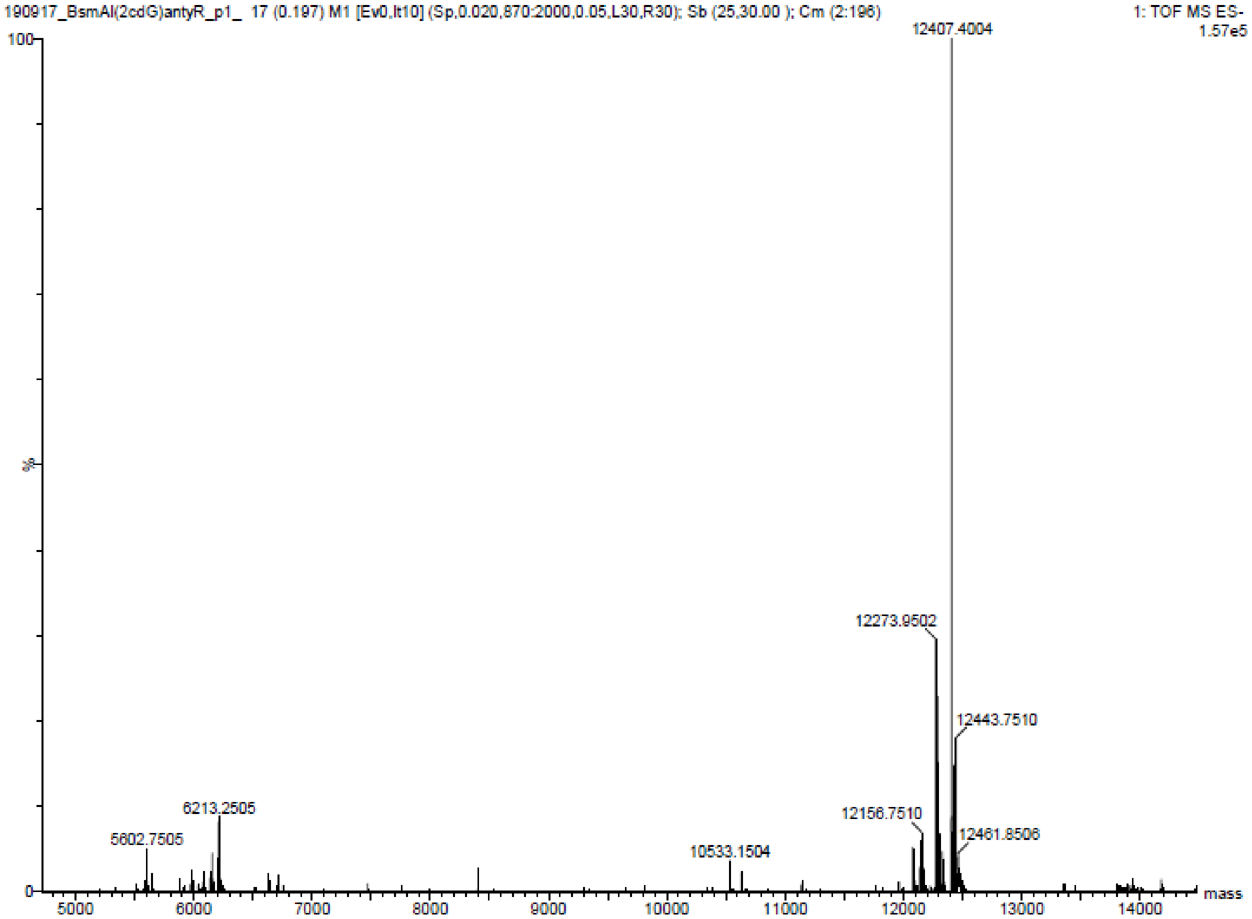

Figure S92. Mass spectrum of sequence 14.

Table S3. The influence of cdPus on the activity of BsmAI. Raw numerical data of bands intensity obtained from Quantity One software.

BsmAI			Time [min]						
			0	1	5	15	30	45	60
Duplex	Strand	Data set	Strand cleavage [%]						
A	1	1.	0,03	0,08	2,44	32,13	93,13		99,32
		2.	0,00	2,30	21,70	63,20	92,75		96,82
		3.	0,12	1,59	28,70	78,82	99,12		100,00
		Avg	0,05	1,32	17,62	58,05	95,00		98,71
		SD	0,06	1,13	13,60	23,77	3,57		1,68
	2	1.	0,00	0,00	8,61	68,13	98,67		99,38
		2.	0,97	4,33	39,65	82,72	95,06		95,07
		3.	0,00	2,71	42,67	94,24	98,86		99,26
		Avg	0,32	2,35	30,31	81,70	97,53		97,90
		SD	0,56	2,19	18,86	13,08	2,14		2,45
B	3	1.	0,08	1,03	10,18	72,40	99,78	99,70	99,44
		2.	0,00	0,70	13,01	69,39	98,91	99,53	90,07

		3.	0,00	1,89	17,12	75,81	99,50	99,38	96,15
		Avg	0,03	1,21	13,44	72,53	99,40	99,54	95,22
		SD	0,05	0,61	3,49	3,21	0,44	0,16	4,75
	4	1.	0,00	1,32	18,48	90,86	98,99	99,39	99,05
		2.	0,00	1,22	22,26	71,10	99,06	99,24	99,13
		3.	0,25	1,70	14,14	84,96	97,07	98,76	99,36
		Avg	0,08	1,41	18,30	82,31	98,37	99,13	99,18
		SD	0,14	0,25	4,06	10,15	1,13	0,33	0,16
C	5	1.	0,00	2,60	31,86	89,16	99,10	98,11	99,01
		2.	0,43	5,94	27,90	88,30	95,66	95,78	95,39
		3.	0,00	3,57	35,63	92,49	99,62	99,44	98,77
		Avg	0,14	4,04	31,80	89,99	98,12	97,78	97,72
		SD	0,25	1,72	3,87	2,21	2,15	1,85	2,03
	6	1.	0,00	2,39	26,36	85,04	98,57	98,14	96,86
		2.	0,13	3,33	22,23	79,32	94,42	92,81	92,42
		3.	0,20	5,12	42,24	91,17	98,88	97,80	97,67
		Avg	0,11	3,61	30,28	85,18	97,29	96,25	95,65
		SD	0,10	1,39	10,57	5,93	2,49	2,98	2,82
D	7	1.	0,01	0,00	0,00	0,15	0,29	0,46	0,54
		2.	0,00	0,01	0,00	0,00	0,00	0,00	0,00
		3.	0,00	0,00	0,00	0,00	0,00	0,00	0,00
		Avg	0,00	0,00	0,00	0,05	0,10	0,15	0,18
		SD	0,00	0,01	0,00	0,09	0,17	0,26	0,31
	8	1.	0,00	0,00	0,00	0,00	0,03	0,02	0,00
		2.	0,00	0,00	0,00	0,00	0,00	0,00	0,00
		3.	0,00	0,00	0,00	0,00	0,00	0,00	0,00
		Avg	0,00	0,00	0,00	0,00	0,01	0,01	0,00
		SD	0,00	0,00	0,00	0,00	0,02	0,01	0,00
E	9	1.	0,00	0,03	1,34	9,96	33,18	42,84	36,25
		2.	0,00	0,55	4,53	15,26	31,68	39,47	48,03
		3.	0,01	0,09	2,96	17,35	43,74	55,53	49,32
		Avg	0,00	0,22	2,94	14,19	36,20	45,95	44,53
		SD	0,01	0,28	1,59	3,81	6,57	8,47	7,20
	10	1.	0,00	0,00	1,83	7,41	22,47	44,16	41,10
		2.	0,52	1,18	3,89	13,26	23,83	32,71	37,64
		3.	0,63	1,53	6,72	26,26	49,73	52,33	54,74
		Avg	0,38	0,90	4,15	15,64	32,01	43,07	44,49
		SD	0,34	0,80	2,45	9,65	15,36	9,85	9,04
F	11	1.	0,92	1,46	19,19	89,49	99,70	99,84	99,84
		2.	0,09	1,03	17,08	73,97	99,02	98,66	98,35
		3.	0,00	0,19	26,48	94,16	99,96	99,97	99,92

		Avg	0,34	0,89	20,92	85,87	99,56	99,49	99,37
		SD	0,51	0,65	4,93	10,57	0,49	0,72	0,89
	12	1.	0,00	0,50	22,35	84,63	98,49	99,67	99,51
		2.	0,00	0,92	11,74	81,80	96,75	99,08	98,61
		3.	0,00	2,73	32,82	91,65	99,75	99,93	99,90
		Avg	0,00	1,38	22,31	86,02	98,33	99,56	99,34
		SD	0,00	1,18	10,54	5,07	1,51	0,43	0,66
G	13	1.	0,00	0,49	14,28	71,43	98,85	98,93	99,12
		2.	0,00	1,63	17,03	52,65	94,71	96,78	97,72
		3.	1,24	3,61	19,53	56,36	95,49	98,15	95,66
		Avg	0,41	1,91	16,94	60,15	96,35	97,95	97,50
		SD	0,72	1,58	2,63	9,95	2,20	1,09	1,74
	14	1.	0,40	4,67	23,92	64,85	95,12	97,15	94,58
		2.	0,52	5,87	30,11	64,77	92,19	97,03	96,25
		3.	0,02	4,14	19,79	69,41	96,16	97,40	96,64
		Avg	0,31	4,89	24,61	66,34	94,49	97,19	95,83
		SD	0,26	0,88	5,19	2,66	2,06	0,19	1,09
H	15	1.	0,13	0,77	18,92	61,18	93,62	96,78	94,55
		2.	0,56	1,86	16,68	70,88	93,74	93,17	94,02
		3.	0,00	0,13	20,23	72,77	97,30	99,58	99,04
		Avg	0,23	0,92	18,61	68,28	94,89	96,51	95,87
		SD	0,29	0,88	1,80	6,22	2,09	3,21	2,76
	16	1.	0,00	0,00	4,81	38,53	54,82	74,45	74,14
		2.	0,00	0,00	2,38	26,67	55,98	67,21	65,06
		3.	0,31	1,47	10,15	46,88	79,22	85,53	87,41
		Avg	0,10	0,49	5,78	37,36	63,34	75,73	75,54
		SD	0,18	0,85	3,97	10,15	13,76	9,23	11,24
I	17	1.	0,40	3,51	24,71	67,47	88,06	88,92	89,59
		2.	0,00	4,34	24,95	65,47	86,61	90,49	90,66
		3.	0,74	6,07	30,44	72,58	91,64	92,79	89,70
		Avg	0,38	4,64	26,70	68,51	88,77	90,73	89,99
		SD	0,37	1,31	3,24	3,67	2,59	1,94	0,59
	18	1.	0,82	3,77	16,63	43,86	63,25	68,22	73,44
		2.	1,49	4,45	16,54	40,31	74,52	71,87	71,29
		3.	0,00	3,11	14,94	45,21	83,58	79,55	76,87
		Avg	0,77	3,78	16,04	43,13	73,78	73,21	73,87
		SD	0,75	0,67	0,95	2,53	10,18	5,78	2,81
J	19	1.	0,00	0,12	2,48	31,98	69,29	87,67	82,97
		2.	0,00	0,00	0,35	16,44	69,66	93,46	85,55
		3.	0,00	0,14	1,52	24,17	62,86	89,04	88,41
		Avg	0,00	0,09	1,45	24,20	67,27	90,06	85,65

		SD	0,00	0,07	1,07	7,77	3,82	3,03	2,72
	20	1.	0,39	0,48	0,67	0,88	1,32	1,58	1,72
		2.	1,66	1,62	2,29	3,58	5,94	7,21	7,28
		3.	1,84	1,95	2,29	3,51	5,29	5,84	5,80
		Avg	1,30	1,35	1,75	2,65	4,18	4,88	4,93
		SD	0,79	0,77	0,94	1,54	2,50	2,94	2,88
K	21	1.	0,00	0,07	0,49	4,10	13,23	21,79	24,12
		2.	0,00	0,00	0,00	1,73	10,06	20,97	21,18
		3.	0,16	0,25	0,46	4,54	12,04	19,40	19,35
		Avg	0,05	0,11	0,32	3,46	11,78	20,72	21,55
		SD	0,09	0,13	0,28	1,51	1,60	1,22	2,40
	22	1.	2,72	2,81	3,38	4,38	8,24	11,77	14,29
		2.	12,54	10,78	12,75	15,29	22,03	28,11	28,14
		3.	9,81	8,06	8,96	10,78	15,67	21,13	22,93
		Avg	8,36	7,22	8,36	10,15	15,31	20,33	21,78
		SD	5,07	4,05	4,71	5,48	6,90	8,20	7,00
L	23	1.	0,08	1,01	9,91	39,00	49,55	47,99	52,19
		2.	0,14	2,85	12,13	38,25	48,85	59,89	56,63
		3.	0,00	2,22	13,03	34,87	56,11	60,09	55,30
		Avg	0,07	2,03	11,69	37,37	51,50	55,99	54,71
		SD	0,07	0,94	1,61	2,20	4,01	6,93	2,28
	24	1.	0,00	0,00	2,15	8,33	16,71	12,83	13,05
		2.	0,00	0,00	1,86	6,38	14,39	13,98	17,56
		3.	0,00	0,00	3,13	13,86	15,14	19,59	27,37
		Avg	0,00	0,00	2,38	9,52	15,41	15,47	19,33
		SD	0,00	0,00	0,67	3,88	1,19	3,62	7,32
M	26	1.	3,90	3,88	6,07	15,08	25,00	26,85	23,91
		2.	16,22	14,73	16,87	22,92	35,97	37,36	33,50
		3.	9,59	10,04	10,65	17,87	24,43	24,28	20,34
		Avg	9,90	9,55	11,20	18,62	28,47	29,50	25,92
		SD	6,17	5,44	5,42	3,97	6,51	6,93	6,81
N	27	1.	0,00	0,44	10,08	41,33	58,28	67,25	67,92
		2.	0,00	1,35	13,38	42,41	63,41	62,84	72,20
		3.	0,00	2,77	16,60	42,59	60,11	65,77	67,91
		Avg	0,00	1,52	13,35	42,11	60,60	65,29	69,34
		SD	0,00	1,18	3,26	0,68	2,60	2,25	2,48
	28	1.	0,00	0,72	3,12	12,91	32,35	29,83	29,70
		2.	0,00	0,00	1,06	12,50	31,67	28,39	22,48
		3.	0,84	1,65	7,22	20,21	34,03	33,62	30,20
		Avg	0,28	0,79	3,80	15,21	32,68	30,61	27,46
		SD	0,48	0,83	3,14	4,33	1,21	2,70	4,32

O	30	1.	4,02	4,43	6,37	16,48	22,70	22,61	23,20
		2.	11,90	13,03	15,48	21,79	27,81	31,66	29,41
		3.	7,32	6,25	7,71	12,91	16,57	12,99	11,03
		Avg	7,74	7,91	9,86	17,06	22,36	22,42	21,21
		SD	3,96	4,53	4,92	4,47	5,63	9,34	9,35

Table S4. The influence of cdPus on the activity of SspI. Raw numerical data of bands intensity obtained from Quantity One software.

SspI			Time [min]						
			0	1	5	15	30	45	60
Duplex	Strand	Data set	Strand cleavage [%]						
A	1	1.	0,08	0,36	2,73	14,48	46,87		83,75
		2.	0,37	1,17	4,79	16,21	37,64		85,93
		3.	0,00	0,00	2,84	24,16	49,31		87,62
		Avg	0,15	0,51	3,45	18,29	44,60		85,77
		SD	0,19	0,60	1,16	5,16	6,15		1,94
	2	1.	0,02	0,13	1,85	19,74	60,40		88,89
		2.	0,00	0,16	3,94	17,60	47,57		72,72
		3.	0,37	0,86	6,14	23,15	40,29		85,02
		Avg	0,13	0,39	3,97	20,17	49,42		82,21
		SD	0,21	0,41	2,15	2,80	10,18		8,44
B	3	1.	0,00	0,00	0,00	0,00	0,00	0,00	0,00
		2.	0,00	0,00	0,00	0,00	0,25	0,00	0,11
		3.	0,00	0,00	0,00	0,00	0,00	0,00	0,00
		Avg	0,00	0,00	0,00	0,00	0,08	0,00	0,04
		SD	0,00	0,00	0,00	0,00	0,15	0,00	0,06
	4	1.	0,00	0,00	0,13	0,00	0,00	0,00	0,00
		2.	0,07	0,00	0,00	0,00	0,00	0,00	0,00
		3.	0,00	0,00	0,00	0,00	0,00	0,02	0,02
		Avg	0,02	0,00	0,04	0,00	0,00	0,01	0,01
		SD	0,04	0,00	0,08	0,00	0,00	0,01	0,01
C	5	1.	0,00	0,00	0,00	0,00	0,00	0,00	0,00
		2.	0,00	0,00	0,00	0,00	0,00	0,00	0,00
		3.	0,37	0,26	0,28	0,11	0,04	0,12	0,35
		Avg	0,12	0,09	0,09	0,04	0,01	0,04	0,12
		SD	0,21	0,15	0,16	0,06	0,02	0,07	0,20
	6	1.	0,00	0,00	0,00	0,00	0,00	0,00	0,00
		2.	0,00	0,00	0,00	0,00	0,00	0,00	0,00
		3.	0,13	0,16	0,12	0,21	0,10	0,04	0,01
		Avg	0,04	0,05	0,04	0,07	0,03	0,01	0,00
		SD	0,08	0,09	0,07	0,12	0,06	0,02	0,00

D	7	1.	0,06	0,47	3,50	27,30	55,23	79,33	94,81
		2.	0,00	0,00	1,38	18,20	48,04	67,24	92,72
		3.	0,00	0,00	1,80	14,96	50,93	63,46	98,73
		Avg	0,02	0,16	2,22	20,15	51,40	70,01	95,42
		SD	0,04	0,27	1,12	6,40	3,62	8,29	3,05
	8	1.	0,01	0,16	2,62	33,42	54,89	66,74	80,17
		2.	0,07	0,47	2,17	13,71	47,75	74,59	92,86
		3.	0,00	0,00	0,49	15,44	44,40	84,93	94,09
		Avg	0,03	0,21	1,76	20,86	49,01	75,42	89,04
		SD	0,04	0,24	1,12	10,91	5,35	9,13	7,71
E	9	1.	0,00	0,63	5,99	32,31	71,12	91,25	97,23
		2.	0,74	1,37	4,07	21,52	56,38	87,71	94,68
		3.	0,00	0,59	4,86	28,14	62,66	79,19	94,94
		Avg	0,25	0,86	4,97	27,32	63,39	86,05	95,62
		SD	0,43	0,44	0,96	5,44	7,40	6,20	1,40
	10	1.	0,00	0,06	4,86	30,40	72,92	94,44	97,96
		2.	0,00	0,28	2,78	21,99	62,68	89,16	91,44
		3.	0,25	0,79	5,83	24,66	67,39	84,05	93,40
		Avg	0,08	0,37	4,49	25,68	67,66	89,22	94,27
		SD	0,14	0,37	1,56	4,30	5,13	5,19	3,34
F	11	1.	0,00	0,00	0,00	0,00	0,00	0,00	0,00
		2.	0,00	0,00	0,00	0,00	0,00	0,00	0,00
		3.	0,00	0,00	0,00	0,00	0,00	0,00	0,00
		Avg	0,00	0,00	0,00	0,00	0,00	0,00	0,00
		SD	0,00	0,00	0,00	0,00	0,00	0,00	0,00
	12	1.	0,00	0,00	0,00	0,00	0,00	0,00	0,00
		2.	0,00	0,00	0,00	0,00	0,00	0,00	0,00
		3.	0,00	0,00	0,00	0,00	0,00	0,00	0,00
		Avg	0,00	0,00	0,00	0,00	0,00	0,00	0,00
		SD	0,00	0,00	0,00	0,00	0,00	0,00	0,00
G	13	1.	0,09	0,11	0,12	1,01	0,35	0,69	1,81
		2.	0,14	0,00	0,00	0,13	0,06	0,00	0,00
		3.	0,00	0,00	0,00	0,00	0,00	0,00	0,00
		Avg	0,08	0,04	0,04	0,38	0,14	0,23	0,60
		SD	0,07	0,06	0,07	0,55	0,19	0,40	1,05
	14	1.	1,02	0,31	0,49	0,06	0,00	0,00	0,00
		2.	0,00	0,00	0,00	0,00	0,00	0,00	0,00
		3.	0,00	0,00	0,00	0,00	0,00	0,00	0,00
		Avg	0,34	0,10	0,16	0,02	0,00	0,00	0,00
		SD	0,59	0,18	0,28	0,03	0,00	0,00	0,00
H	15	1.	0,00	0,00	2,31	12,34	38,45	66,54	83,94

		2.	0,47	1,36	4,51	14,39	36,84	53,96	75,05
		3.	0,00	0,05	1,32	14,89	35,23	59,59	82,67
		Avg	0,16	0,47	2,71	13,87	36,84	60,03	80,55
		SD	0,27	0,77	1,63	1,35	1,61	6,30	4,81
	16	1.	0,00	0,00	1,72	13,34	42,17	77,84	93,28
		2.	0,00	0,00	2,17	15,22	32,27	53,84	60,49
		3.	0,00	0,00	2,33	15,43	38,80	50,35	82,63
		Avg	0,00	0,00	2,07	14,66	37,74	60,68	78,80
		SD	0,00	0,00	0,31	1,15	5,04	14,96	16,73
I	17	1.	0,00	0,82	2,92	17,79	37,52	57,45	73,24
		2.	2,24	3,28	6,20	22,34	43,38	65,53	73,41
		3.	0,00	0,93	6,68	25,21	54,69	74,13	87,41
		Avg	0,75	1,68	5,26	21,78	45,20	65,70	78,02
		SD	1,29	1,39	2,05	3,74	8,73	8,34	8,14
	18	1.	0,00	0,03	1,79	19,21	38,94	65,55	85,84
		2.	0,00	0,75	7,30	28,89	51,60	66,14	82,84
		3.	0,00	0,00	4,08	20,79	49,54	83,06	91,21
		Avg	0,00	0,26	4,39	22,96	46,70	71,58	86,63
		SD	0,00	0,42	2,77	5,19	6,79	9,94	4,24
J	19	1.	0,00	0,20	2,42	16,63	39,34	47,82	73,51
		2.	0,14	0,96	4,82	23,30	45,63	66,37	80,01
		3.	0,00	0,00	2,26	15,45	38,10	55,56	67,57
		Avg	0,05	0,39	3,17	18,46	41,02	56,58	73,70
		SD	0,08	0,51	1,44	4,23	4,04	9,32	6,22
	20	1.	0,04	0,46	11,22	39,73	56,74	73,61	83,45
		2.	0,00	0,17	10,08	35,21	44,93	56,57	78,79
		3.	0,30	0,73	8,18	38,37	51,62	78,38	91,28
		Avg	0,11	0,45	9,83	37,77	51,10	69,52	84,51
		SD	0,16	0,28	1,53	2,32	5,92	11,46	6,31
K	21	1.	0,00	0,00	1,13	7,87	17,34	36,06	55,52
		2.	0,00	0,43	3,05	24,52	56,18	74,87	88,33
		3.	0,00	0,34	2,88	12,07	31,04	57,92	60,86
		Avg	0,00	0,26	2,35	14,82	34,85	56,29	68,24
		SD	0,00	0,23	1,06	8,66	19,69	19,46	17,61
	22	1.	0,10	0,04	0,57	4,78	17,09	33,38	44,22
		2.	0,00	0,00	0,86	9,35	19,13	22,79	45,87
		3.	0,00	0,00	1,56	12,57	25,99	39,94	48,76
		Avg	0,03	0,01	1,00	8,90	20,74	32,04	46,28
		SD	0,06	0,02	0,51	3,91	4,66	8,66	2,30
L	23	1.	0,52	0,60	0,67	0,64	0,60	0,59	0,52
		2.	0,20	0,72	0,39	0,64	0,93	0,93	1,28

		3.	0,51	0,55	0,76	0,34	0,35	0,33	0,18
		Avg	0,41	0,62	0,61	0,54	0,63	0,62	0,66
		SD	0,18	0,08	0,20	0,17	0,29	0,30	0,56
	24	1.	0,19	0,20	0,16	0,22	0,26	0,38	0,41
		2.	0,00	0,00	0,00	0,00	0,00	0,00	0,00
		3.	0,03	0,06	0,02	0,01	0,00	0,00	0,06
		Avg	0,08	0,09	0,06	0,08	0,09	0,13	0,15
		SD	0,10	0,10	0,09	0,12	0,15	0,22	0,22
M	26	1.	0,00	0,00	0,00	0,00	0,00	0,04	0,11
		2.	0,00	0,00	0,09	0,41	0,53	0,35	0,27
		3.	0,00	0,00	0,00	0,00	0,18	0,02	0,24
		Avg	0,00	0,00	0,03	0,14	0,24	0,14	0,21
		SD	0,00	0,00	0,05	0,24	0,27	0,18	0,08
N	27	1.	0,64	0,68	0,70	0,64	0,59	0,37	0,54
		2.	0,46	0,93	1,29	1,61	1,82	2,00	1,81
		3.	0,18	0,20	0,21	0,09	0,18	0,25	0,02
		Avg	0,43	0,60	0,74	0,78	0,86	0,87	0,79
		SD	0,23	0,37	0,54	0,77	0,85	0,98	0,92
	28	1.	0,54	0,75	0,71	0,75	0,83	0,88	1,05
		2.	0,00	0,00	0,00	0,00	0,00	0,00	0,00
		3.	0,20	0,19	0,00	0,00	0,00	0,00	0,00
		Avg	0,25	0,32	0,24	0,25	0,28	0,29	0,35
		SD	0,27	0,39	0,41	0,43	0,48	0,51	0,61
O	30	1.	0,00	0,00	0,00	0,00	0,00	0,13	0,00
		2.	0,00	0,00	0,00	0,00	0,00	0,00	0,00
		3.	0,00	0,00	0,00	0,00	0,00	0,00	0,00
		Avg	0,00	0,00	0,00	0,00	0,00	0,04	0,00
		SD	0,00	0,00	0,00	0,00	0,00	0,07	0,00

		End	1	2	3	4	5	6	7	8	9	10	11	12	13	14	15	16	17	18	19	20	21	22	23	24	25	26	27	28	29	30	31	32	33	34	35	36	37	38	39	40	End	Cleavage (60min) [%]	
																																												BsmAI	SspI
A	1	5'	C	T	C	T	T	G	T	C	A	G	G	A	A	T	A	T	T	G	T	C	T	C	T	A	T	G	C	T	C	C	C	A	C	C	A	A	A	G	G	C	3'	98,71	85,71
	2	3'	G	A	G	A	A	C	A	G	T	C	C	T	T	A	T	A	A	C	A	G	A	G	A	T	A	C	G	A	G	G	G	T	G	G	T	T	T	C	C	G	5'	97,90	82,21
B	3	5'	C	T	C	T	T	G	T	C	A	G	G	A	A	T	SA	T	T	G	T	C	T	C	T	A	T	G	C	T	C	C	C	A	C	C	A	A	A	G	G	C	3'	95,22	0,04
	4	3'	G	A	G	A	A	C	A	G	T	C	C	T	T	A	T	A	A	C	A	G	A	G	A	T	A	C	G	A	G	G	G	T	G	G	T	T	T	C	C	G	5'	99,18	0,01
C	5	5'	C	T	C	T	T	G	T	C	A	G	G	A	A	T	RA	T	T	G	T	C	T	C	T	A	T	G	C	T	C	C	C	A	C	C	A	A	A	G	G	C	3'	97,72	0,12
	6	3'	G	A	G	A	A	C	A	G	T	C	C	T	T	A	T	A	A	C	A	G	A	G	A	T	A	C	G	A	G	G	G	T	G	G	T	T	T	C	C	G	5'	95,65	0,00
D	7	5'	C	T	C	T	T	G	T	C	A	G	G	A	A	T	A	T	T	G	T	C	T	C	T	SA	T	G	C	T	C	C	C	A	C	C	A	A	A	G	G	C	3'	0,18	95,42
	8	3'	G	A	G	A	A	C	A	G	T	C	C	T	T	A	T	A	A	C	A	G	A	G	A	T	A	C	G	A	G	G	G	T	G	G	T	T	T	C	C	G	5'	0,00	89,04
E	9	5'	C	T	C	T	T	G	T	C	A	G	G	A	A	T	A	T	T	G	T	C	T	C	T	RA	T	G	C	T	C	C	C	A	C	C	A	A	A	G	G	C	3'	44,53	95,62
	10	3'	G	A	G	A	A	C	A	G	T	C	C	T	T	A	T	A	A	C	A	G	A	G	A	T	A	C	G	A	G	G	G	T	G	G	T	T	T	C	C	G	5'	44,49	94,27
F	11	5'	C	T	C	T	T	G	T	C	A	G	G	A	A	T	A	T	T	G	T	C	T	C	T	A	T	G	C	T	C	C	C	A	C	C	A	A	A	G	G	C	3'	99,37	0,00
	12	3'	G	A	G	A	A	C	A	G	T	C	C	T	T	SA	T	A	A	C	A	G	A	G	A	T	A	C	G	A	G	G	G	T	G	G	T	T	T	C	C	G	5'	99,34	0,00
G	13	5'	C	T	C	T	T	G	T	C	A	G	G	A	A	T	A	T	T	G	T	C	T	C	T	A	T	G	C	T	C	C	C	A	C	C	A	A	A	G	G	C	3'	97,50	0,60
	14	3'	G	A	G	A	A	C	A	G	T	C	C	T	T	RA	T	A	A	C	A	G	A	G	A	T	A	C	G	A	G	G	G	T	G	G	T	T	T	C	C	G	5'	95,83	0,00
H	15	5'	C	T	C	T	T	G	T	C	A	G	G	A	A	T	A	T	T	G	T	C	T	C	T	A	T	G	C	T	C	C	C	A	C	C	A	A	A	G	G	C	3'	95,87	80,55
	16	3'	G	A	G	A	A	C	A	G	T	C	C	T	T	A	T	A	A	C	A	G	A	G	SA	T	A	C	G	A	G	G	G	T	G	G	T	T	T	C	C	G	5'	75,54	78,80
I	17	5'	C	T	C	T	T	G	T	C	A	G	G	A	A	T	A	T	T	G	T	C	T	C	T	A	T	G	C	T	C	C	C	A	C	C	A	A	A	G	G	C	3'	89,99	78,02
	18	3'	G	A	G	A	A	C	A	G	T	C	C	T	T	A	T	A	A	C	A	G	A	G	RA	T	A	C	G	A	G	G	G	T	G	G	T	T	T	C	C	G	5'	73,87	86,63
J	19	5'	C	T	C	T	T	G	T	C	A	G	G	A	A	T	A	T	T	G	T	C	T	C	T	A	T	G	C	T	C	C	C	A	C	C	A	A	A	G	G	C	3'	85,65	73,70
	20	3'	G	A	G	A	A	C	A	G	T	C	C	T	T	A	T	A	A	C	A	G	A	G	A	T	A	C	SG	A	G	G	G	T	G	G	T	T	T	C	C	G	5'	4,93	84,51
K	21	5'	C	T	C	T	T	G	T	C	A	G	G	A	A	T	A	T	T	G	T	C	T	C	T	A	T	G	C	T	C	C	C	A	C	C	A	A	A	G	G	C	3'	21,55	68,24
	22	3'	G	A	G	A	A	C	A	G	T	C	C	T	T	A	T	A	A	C	A	G	A	G	A	T	A	C	RG	A	G	G	G	T	G	G	T	T	T	C	C	G	5'	21,78	46,28
L	23	5'	C	T	C	T	T	G	T	C	A	G	G	A	SA	T	A	T	T	G	T	C	T	C	T	A	T	G	C	T	C	C	C	A	C	C	A	A	A	G	G	C	3'	54,71	0,66
	24	3'	G	A	G	A	A	C	A	G	T	C	C	T	T	A	T	A	A	C	A	G	A	SG	A	T	A	C	G	A	G	G	G	T	G	G	T	T	T	C	C	G	5'	19,33	0,15
M	25	5'	C	T	C	T	T	G	T	C	A	G	G	A	RA	T	A	T	T	G	T	C	T	C	T	A	T	G	C	T	C	C	C	A	C	C	A	A	A	G	G	C	3'	N/A	
	26	3'	G	A	G	A	A	C	A	G	T	C	C	T	T	A	T	A	A	C	A	G	A	RG	A	T	A	C	G	A	G	G	G	T	G	G	T	T	T	C	C	G	5'	25,92	0,21
N	27	5'	C	T	C	T	T	G	T	C	A	G	G	A	RA	T	A	T	T	G	T	C	T	C	T	A	T	G	C	T	C	C	C	A	C	C	A	A	A	G	G	C	3'	69,34	0,79
	28	3'	G	A	G	A	A	C	A	G	T	C	C	T	T	A	T	A	A	C	A	G	A	SG	A	T	A	C	G	A	G	G	G	T	G	G	T	T	T	C	C	G	5'	27,46	0,35
O	29	5'	C	T	C	T	T	G	T	C	A	G	G	A	SA	T	A	T	T	G	T	C	T	C	T	A	T	G	C	T	C	C	C	A	C	C	A	A	A	G	G	C	3'	N/A	
	30	3'	G	A	G	A	A	C	A	G	T	C	C	T	T	A	T	A	A	C	A	G	A	RG	A	T	A	C	G	A	G	G	G	T	G	G	T	T	T	C	C	G	5'	21,21	0,00

Figure S93. The sequence of substrate oligonucleotides containing 5',8-cyclo-2'-deoxypurines (cdPus). SX - (5'S)-5'8-cyclo-2'-deoxyadenosine; RX - (5'R)-5'8-cyclo-2'-deoxyadenosine; SY - (5'S)-5'8-cyclo-2'-deoxyguanosine; RY - (5'R)-5'8-cyclo-2'-deoxyguanosine. The cleavage value of each strand after 60 min by 0.5 U BsmAI and 1.5 U SspI is shown.
